# Supplementary material for: Unravelling the complex causal effects of substance use behaviours on common diseases
Source: Commun Med (Lond). 2024 Mar 12;4:43. doi: 10.1038/s43856-024-00473-3 (PMC10933313; doi:10.1038/s43856-024-00473-3)
Supplement: Supplementary file 2 — Supplementary Information [file 43856_2024_473_MOESM2_ESM.pdf]

# Unravelling the complex causal effects of substance use behaviours on common diseases

Angli Xue[1,2,3], Zhihong Zhu[1,4], Huanwei Wang[1], Longda Jiang[1], Peter M. Visscher[1], Jian Zeng[1], Jian Yang[1,5,6,*]

[1] Institute for Molecular Bioscience, The University of Queensland, Brisbane, Queensland, 4072, Australia
[2] Garvan-Weizmann Centre for Cellular Genomics, Garvan Institute of Medical Research, Sydney, NSW, 2010, Australia
[3] School of Biomedical Sciences, University of New South Wales, Sydney, NSW, 2052, Australia
[4] National Centre for Register-Based Research, Aarhus University, Aarhus V, 8210, Denmark
[5] School of Life Sciences, Westlake University, Hangzhou, Zhejiang 310024, China
[6] Westlake Laboratory of Life Sciences and Biomedicine, Hangzhou, Zhejiang 310024, China

[*]Correspondence: J.Y. (jian.yang@westlake.edu.cn)

**Contents**

**Supplementary Notes 1 to 4**
**Supplementary Tables 1 to 5**
**Supplementary Figures 1 to 24**

## Supplementary Notes

### Supplementary Note 1 Improving the GSMR method (GSMR2)

We have previously introduced the GSMR method along with the HEIDI-outlier filtering approach[29] that utilizes multiple independent genome-wide significant SNPs to test for a causal association between two traits, accounting for residual LD between SNPs. The HEIDI-outlier filtering method was designed to remove invalid IVs with horizontal pleiotropic effects. However, since the core assumptions of MR framework[54] are not always satisfied in the real data, there remain challenges when applying the HEIDI-outlier filtering method. First, the original HEIDI-outlier filtering algorithm assumes that the causal effect ($\hat{b}_{xy}$) estimated at the focal IV (the SNP with the lowest association *P*-value in the third quintile of the distribution of $\hat{b}_{xy}$) is an unbiased estimate of the true causal effect. Thus, the accuracy of the estimation would be largely dependent on the focal IV. Second, when there is strong directional pleiotropy, selecting the top SNP within a specific quantile of the distribution of $\hat{b}_{xy}$ as the focal IV could be problematic. To overcome these limitations, we propose a global heterogeneity test (called the global HEIDI-outlier test) and improve the focal IV selection algorithm by excluding the pleiotropic IVs iteratively rather than fixing a specific IV as the focal IV. Compared to the previous algorithm, the improved algorithm is more conservative and spontaneous but without sacrificing the power.

The global HEIDI-outlier filtering algorithm runs as follows:

(1) Selecting each clumped genome-wide significant SNP as a focal IV, and testing the heterogeneity between the focal IV and all the other IVs by calculating a global $p_{\text{HEIDI}}$ using "*pchisum*" function in the "*survey*" R package;

(2) If the global $p_{\text{HEIDI}}$ of any IV is smaller than 0.01, the IV with the smallest global $p_{\text{HEIDI}}$ will be removed;

(3) Repeating (1-2) until none of the IVs have a global $p_{\text{HEIDI}} < 0.01$.

In our simulation study, the new GSMR method (named GSMR2) outperformed the original version in most scenarios (**Supplementary Figures 1-3**). Thus, we used GSMR2 in real data analysis in lieu of the original version.

### Supplementary Note 2 Comparison of different MR methods by simulation

We ran simulation to demonstrate the statistical power and false positive rate (FPR) for 11 commonly used MR methods. The equations to generate the exposure and outcome phenotypes are presented below.

$$x = \sum_{i}^{m_x} z_{x(i)}\, b_{zx(i)} + \sum_{j}^{m_p} z_{p(j)}\, b_{zpx(j)} + cb_{cx} + e_x \qquad (1)$$

$$y = xb_{xy} + \sum_{j}^{m_p} z_{p(j)}\, b_{zpy(j)} + \sum_{k}^{m_y} z_{y(k)}\, b_{zy(k)} + cb_{cy} + e_y \qquad (2)$$

*x*: exposure phenotype with $var(x) = 1$.

*y*: outcome phenotype with $var(y) = 1$.

*z*: standardized genotype for $m = 300$ SNPs ($m = m_x + m_p + m_y$), $z = \frac{\tilde{z} - f}{\sqrt{2f(1-f)}}$ with genotype $\tilde{z}$ generated from $B(2, f)$ and the allele frequency *f* generated from $U(0.01, 0.5)$.

$z_x$: standardized genotype for $m_x$ SNPs with an effect on *x* only;

$z_p$: standardized genotype for $m_p$ SNPs with a pleiotropic effect on both *x* and *y*;

$z_y$: standardized genotype for $m_y$ SNPs with an effect on *y* only;

$(m_x, m_p, m_y) \in \{(200,0,100), (180,20,100), (160,40,100), (140,60,100), (100,100,100)\}$.

*b*: $b_{zx}, b_{zy}, b_{zpx}, b_{zpy}$ are generated from $N(0,1)$. The causal effect $b_{xy}$ is set as {0, 0.1, -0.1}.

*c*: non-genetic confounder, generated from $N(0,1)$. The $b_{cx}$ is set as 0.3.

*e*: residuals, generated from $N(0,1)$.

The total phenotypic variance explained by the causal variants is 0.1 ($h_{zx}^2 = h_{zy}^2 = 0.1$). The phenotypic variance explained by the pleiotropic causal variants ranges from 0.01 to 0.05 ($h_{zp}^2 \in \{0.01, 0.02, 0.03, 0.04, 0.05\}$).

We also simulated directional pleiotropy by setting covariance between the effects of pleiotropic variants on *x* and *y*:

$$\begin{bmatrix} b_{zpx} \\ b_{zpy} \end{bmatrix} \sim N(\mu, \Sigma)\ ,\ with\ \mu = \begin{bmatrix} 0 \\ 0 \end{bmatrix}\ and\ \Sigma = \begin{bmatrix} 1 & cov(b_{zpx}, b_{zpy}) \\ cov(b_{zpx}, b_{zpy}) & 1 \end{bmatrix}, \qquad (3)$$

with $cov(b_{zpx}, b_{zpy}) \in \{-0.3, -0.1, -0.05, 0.05, 0.1, 0.3\}$.

Besides the simulations based on a polygenic model above, we also performed simulations based on a more sparse genetic architecture by setting a small number (*k*) of causal variants whose average variance explained is *T* times larger than the mean for the other causal variants, with $T \in c(10, 30, 50, 100)$ and *k* drawn from a Poisson distribution ($\lambda = 1$). If $k \leq 1$, then we redrew it until $k > 1$. We ran GWAS on the simulated genotype and phenotype and selected the IVs with *p*-values < 5E-8 for the subsequent MR analysis. This means that not all the IVs in the simulated genotype were used in the causal effect estimation. For example, under the scenario of 100-100-100, where there were 100 independent causal SNPs for the simulated exposure, only 5~21 SNPs passed the genome-wide significant threshold and were selected as IVs for MR analysis.

The simulation results were presented in **Supplementary Figures 1-3**. Under the null model and balanced pleiotropy ($b_{xy} = 0$ and $cov(b_{zpx}, b_{zpy}) = 0$) and when the pleiotropic effects were modest ($h^2_{zp} \in \{0.01, 0.02, 0.03, 0.04\}$), the FPR of all the methods were well controlled (<0.05) except for Con-Mix. When the pleiotropic effects were strong ($h^2_{zp} \in \{0.05,\}$), only two methods were well controlled: Median and Mode methods. The FPR increased when the proportion of pleiotropic variants (i.e., invalid IVs) became larger. When comparing GSMR and GSMR2, the FPR was almost the same when the pleiotropic effects are modest, but the FPR of GSMR2 was lower than GSMR in the most cases when the directional pleiotropic effects were strong, indicating the improvement of the new HEIDI-outlier test. All methods produced unbiased estimates for $b_{xy}$ when pleiotropic effects were modest (**Supplementary Figure 2**). Under the most extreme scenarios where 50% IVs were invalid and the pleiotropic effects were strongly negatively correlated between the exposure and outcome ($h^2_{zp} \in \{0.05\}$ and $cov(b_{zpx}, b_{zpy}) = -0.2$), most methods showed biased estimates for $b_{xy}$, with GSMR2, Mode, and MRMix tending to be more robust than the other methods (top-left panel of **Supplementary Figure 2**).

Under the causal model and balanced pleiotropy ($b_{xy} = 0.1$ and $cov(b_{zpx}, b_{zpy}) = 0$), when the pleiotropic effects were modest, MR-Egger, Mode, and MRMix showed substantially lower power than the rest of methods (**Supplementary Figure 3**). With the increase of the proportion of invalid IVs and variance explained by pleiotropic IVs, the power of IVW, RAPS, and Robust slightly decreased. When the directional pleiotropy was in the opposite direction of the causal effect, the power decreased when the strength of the directional pleiotropy increased (from -0.05 to -0.2).

**Supplementary Note 3 Dosage-dependent regression between coffee/tea intake and diseases**

To investigate the dose-dependent effects of coffee and tea intake on the 18 common diseases, we divided the intake levels into 10 groups and formed 10 new intake phenotypes by contracting the intake levels in each of the 10 groups to the non-drinkers. The 10 groups were defined by the daily intake from 1 to 9 cups, plus a group with intake > 10 cups. We then performed logistic regression of each disease against each of the new intake phenotype with sex, age, and the first 10 PCs fitted as fixed covariates. The results showed that the odds ratio (OR) changed the direction for some diseases and the turning point also varied for different diseases (**Supplementary Figures 11-12**). A special case was the association between TI and type 2 diabetes, where all 10 tea intake groups showed lower disease risk than the non-drinkers.

**Supplementary Note 4 Acknowledgement to UK Biobank**

This study has been conducted using UK Biobank resource under Application Number 12514. UK Biobank was established by the Wellcome Trust medical charity, Medical Research Council,

Department of Health, Scottish Government and the Northwest Regional Development Agency. It has also had funding from the Welsh Assembly Government, British Heart Foundation and Diabetes UK.

**Supplementary Table 1** Recommendation of MR methods under different scenarios

| **Causal model** | **Directional pleiotropy** | **Recommended methods** |
|---|---|---|
| Causal | No | GSMR2, IVW, Lasso, RAPS, Robust, PRESSO, Con-Mix |
| Causal | Low | Except Egger, Mode, MRMix |
| Causal | Strong | GSMR2 |
| Null | No | All |
| Null | Low | Median, Mode, RAPS |
| Null | Strong | Median, Mode |

Notes: These recommendations are made based on the simulation results from the current study. We acknowledge that the simulation settings may not cover all the scenarios in the real data, such as number of IVs.

**Supplementary Table 2** Description of substance use behaviours in UK Biobank

| **Trait name** | ***abbr.*** | **binary/continuous** | **sample size** | **cases** | **controls** | **prevalence** |
|---|---|---|---|---|---|---|
| Smoking initiation | SI | binary | 453,693 | 208,988 | 244,705 | 0.461 |
| Former smoking | FS | binary | 406,274 | 161,569 | 244,705 | 0.398 |
| Current smoking | CS | binary | 292,124 | 47,419 | 244,705 | 0.162 |
| Smoking cessation | SC | binary | 208,988 | 161,569 | 47,419 | 0.773 |
| Alcohol consumption | AC | continuous | 454,648 | / | / | / |
| Tea intake | TI | continuous | 440,094 | / | / | / |
| Coffee intake | CI | continuous | 421,947 | / | / | / |

**Supplementary Table 3** Description for 18 common disease traits in UKB

| Trait name | *abbr.* | Data type | N | Cases | Controls | Prevalence |
|---|---|---|---|---|---|---|
| asthma | ASTHMA | binary | 455,607 | 58,892 | 396,715 | 0.129 |
| allergic rhinitis | ALLERGIC_RHINITIS | binary | 455,607 | 28,244 | 427,363 | 0.062 |
| cardiovascular disease | CARD | binary | 455,607 | 74,403 | 381,204 | 0.163 |
| cancer | CANCER | binary | 455,607 | 66,005 | 389,602 | 0.145 |
| major depressive disorder | DEPRESS | binary | 455,607 | 35,310 | 420,297 | 0.078 |
| type 2 diabetes | DIA2 | binary | 455,607 | 27,290 | 428,317 | 0.060 |
| dyslipidemia | DYSLIPID | binary | 455,607 | 79,482 | 376,125 | 0.175 |
| hypertensive disease | HYPER | binary | 455,607 | 88,316 | 367,291 | 0.194 |
| hemorrhoids | HEMORRHOIDS | binary | 455,607 | 27,148 | 428,459 | 0.060 |
| hernia abdominopelvic cavity | HERNIA_ABDOMINOPELVIC | binary | 455,607 | 36,013 | 419,594 | 0.079 |
| iron deficiency anemias | IRON_DEFICIENCY | binary | 455,607 | 10,706 | 444,901 | 0.024 |
| irritable bowel syndrome | IRRITABLE_BOWEL | binary | 455,607 | 15,577 | 440,030 | 0.034 |
| osteoarthritis | OSTIOA | binary | 455,607 | 51,924 | 403,683 | 0.114 |
| osteoporosis | OSTIOP | binary | 455,607 | 11,936 | 443,671 | 0.026 |
| peripheral vascular disease | PVD | binary | 455,607 | 10,370 | 445,237 | 0.023 |
| peptic ulcers | PEPTIC_ULCERS | binary | 455,607 | 12,388 | 443,219 | 0.027 |
| psychiatric disorder | PSYCHIATRIC | binary | 455,607 | 15,321 | 440,286 | 0.034 |
| varicose veins of lower extremities | VARICOSE_VEINS | binary | 455,607 | 13,339 | 442,268 | 0.029 |
| disease count | SUM_OF_CASES | continuous | 455,607 | / | / | / |

**Supplementary Table 4** Description for 12 disease traits from publicly available data sets

| **Trait name** | ***abbr.*** | **sample size** | **cases** | **controls** | **prevalence** | **PUBMED ID** |
|---|---|---|---|---|---|---|
| Coronary Artery Disease | CAD | 547,261 | 122,733 | 424,528 | 0.224 | 29212778 |
| Type 2 diabetes | T2D | 159,208 | 26,676 | 132,532 | 0.168 | 30297969 |
| Crohn's disease | CD | 20,883 | 5,956 | 14,927 | 0.285 | 26192919 |
| Ulcerative colitis | UC | 27,432 | 6,968 | 20,464 | 0.254 | 26192919 |
| Rheumatoid arthritis | RA | 58,284 | 14,361 | 43,923 | 0.246 | 24390342 |
| Schizophrenia | SCZ | 150,064 | 36,989 | 113,075 | 0.246 | 25056061 |
| Bipolar disorder | BIP | 16,731 | 7,481 | 9,250 | 0.447 | 29906448 |
| Major depression disorder | MDD | 71,000 | 60,000 | 11,000 | 0.845 | 29700475 |
| Alzheimer’s disease | AD | 54,162 | 17,008 | 37,154 | 0.314 | 24162737 |
| Ovarian cancer | / | 66,450 | 25,509 | 40,941 | 0.384 | 28346442 |
| Breast cancer | / | 228,951 | 122,977 | 105,974 | 0.537 | 29059683 |
| Prostate cancer | / | 74,849 | 46,939 | 27,910 | 0.627 | 29892016 |

**Supplementary Table 5** Health-related outcomes in different coffee intake and sugar/sweetener use groups in 24h diet recall

| Coffee intake in 24h | Sugar added | Artificial sweetener added | N | BMI | Obesity rate | Disease count |
|---|---|---|---|---|---|---|
| No | No | No | 18820 | 27.13 | 0.233 | 1.33 |
| Yes | No | No | 31394 | 26.99 | 0.214 | 1.22 |
| Yes | No | Yes | 3844 | 28.85 | 0.345 | 1.71 |
| Yes | Yes | No | 9238 | 26.83 | 0.192 | 1.32 |
| Yes | Yes | Yes | 490 | 28.53 | 0.315 | 1.82 |

Note: 105 individuals with incomplete sugar/sweetener records were excluded.

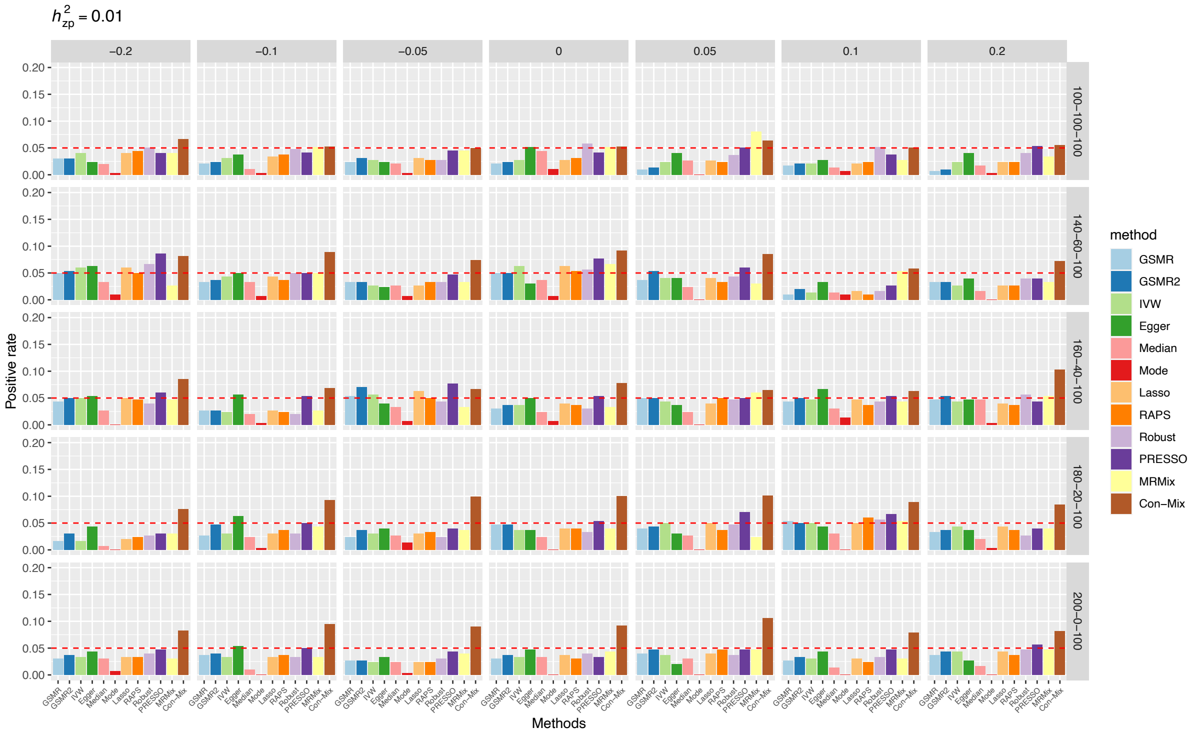
$h^2_{zp} = 0.01$
−0.2
−0.1
−0.05
0
0.05
0.1
0.2
100−100−100
140−60−100
160−40−100
180−20−100
200−0−100
Positive rate
Methods
method
GSMR
GSMR2
IVW
Egger
Median
Mode
Lasso
RAPS
Robust
PRESSO
MRMix
Con−Mix

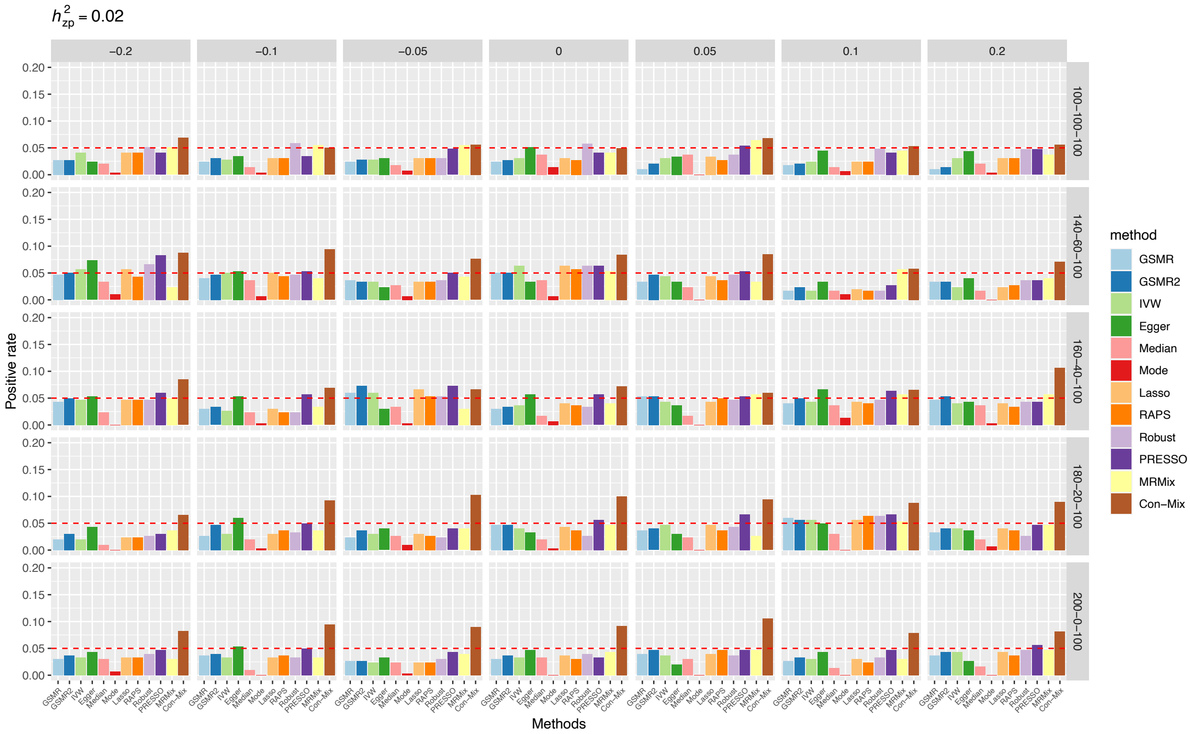
$h^2_{zp} = 0.02$
−0.2
−0.1
−0.05
0
0.05
0.1
0.2
100−100−100
140−60−100
160−40−100
180−20−100
200−0−100
Positive rate
Methods
method
GSMR
GSMR2
IVW
Egger
Median
Mode
Lasso
RAPS
Robust
PRESSO
MRMix
Con−Mix

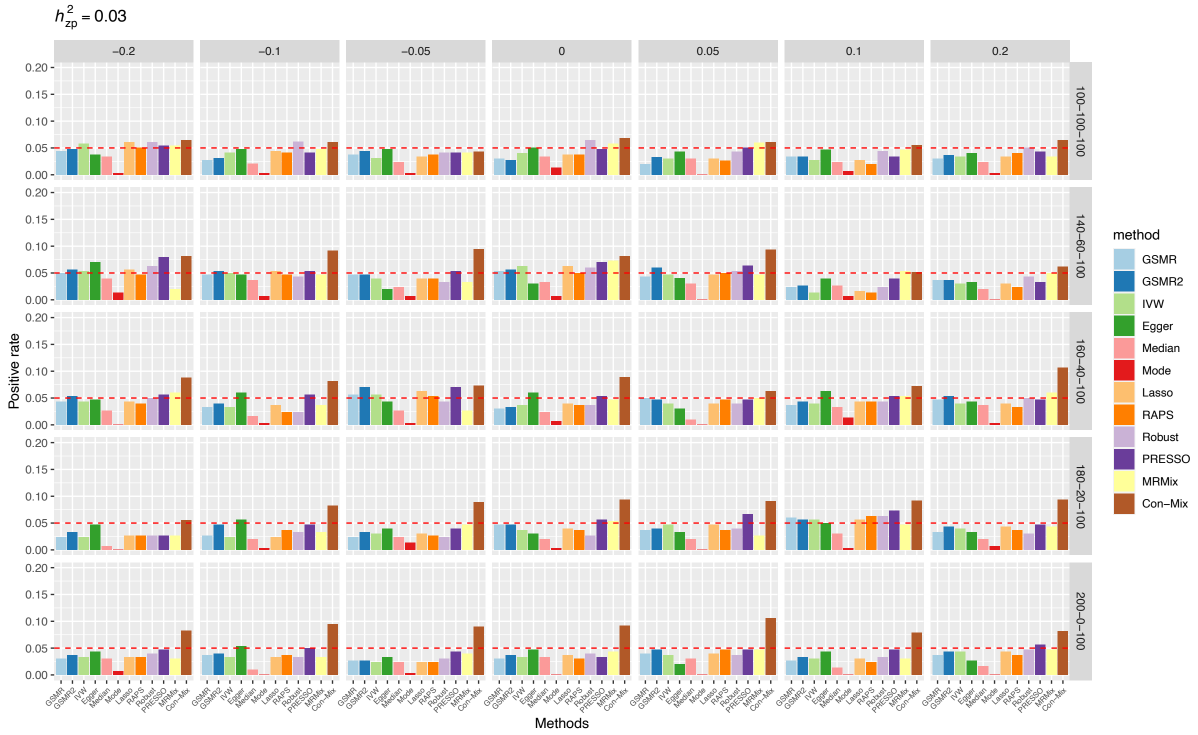
$h_{zp}^2 = 0.03$
−0.2
−0.1
−0.05
0
0.05
0.1
0.2
100−100−100
140−60−100
160−40−100
180−20−100
200−0−100
Positive rate
Methods
method
GSMR
GSMR2
IVW
Egger
Median
Mode
Lasso
RAPS
Robust
PRESSO
MRMix
Con−Mix

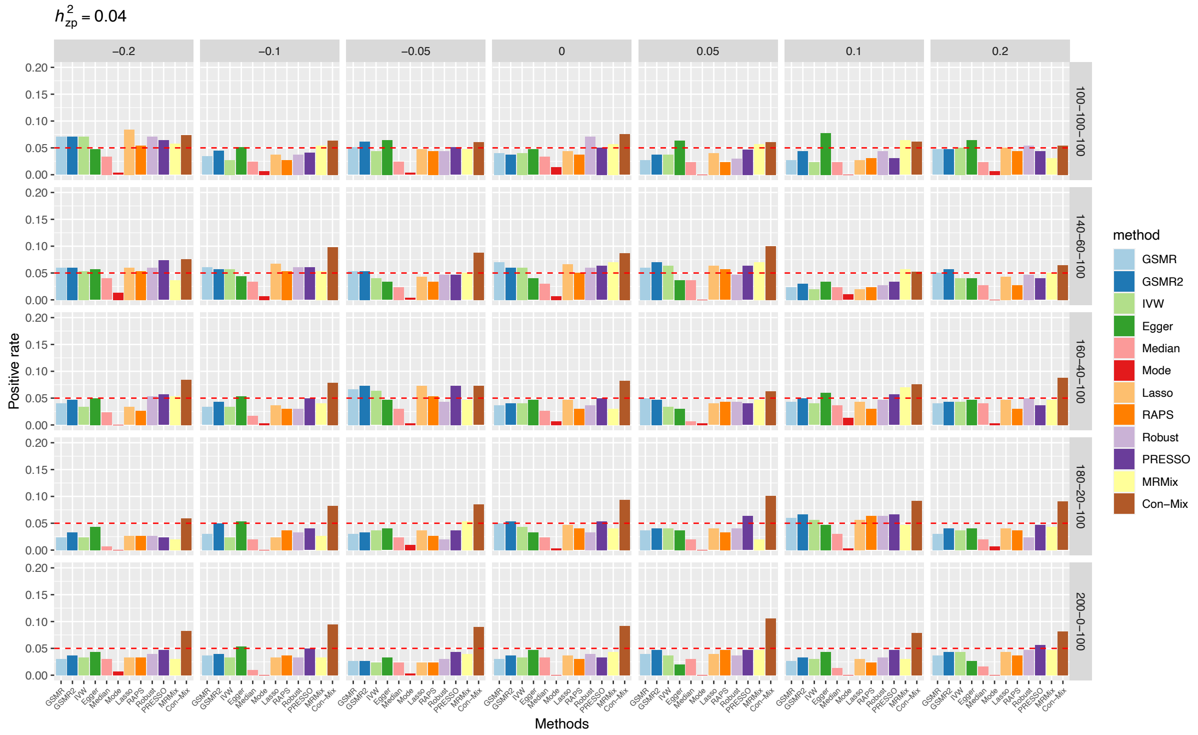
$h_{zp}^2 = 0.04$
−0.2
−0.1
−0.05
0
0.05
0.1
0.2
100−100−100
140−60−100
160−40−100
180−20−100
200−0−100
Positive rate
Methods
method
GSMR
GSMR2
IVW
Egger
Median
Mode
Lasso
RAPS
Robust
PRESSO
MRMix
Con−Mix

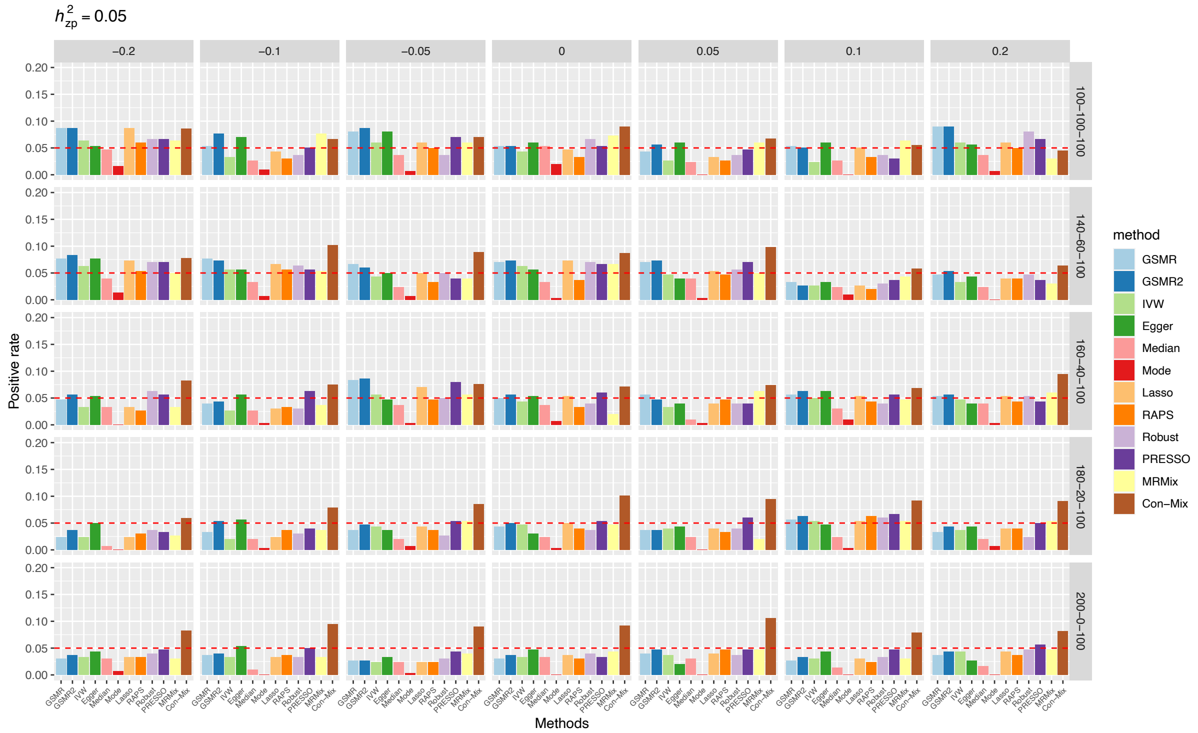


**Supplementary Figure 1. Comparison of false positive rate between the MR methods by simulation under the null model**. There are five panels in total, each of which indicates a different level of variance explained by the pleiotropic variants (i.e., $h_{zp}^2 = 0.01, 0.02, 0.03, 0.04, 0.05$) annotated on the top left of each plot. In each panel, there are 35 facets of bar plots matching with different settings. The column facets indicate 7 different parameters for balanced or directional pleiotropy ($cov(b_{zpx}, b_{zpy})$). The row facets indicate 5 different combinations of numbers of causal and pleiotropic variants ($m_x - m_p - m_y$). The x-axis and colour of the bar denote different MR methods. The y-axis indicates the positive rate from 0 to 1. The red dashed line indicates positive rate equals to 0.05.

$h^2_{zp} = 0.01$

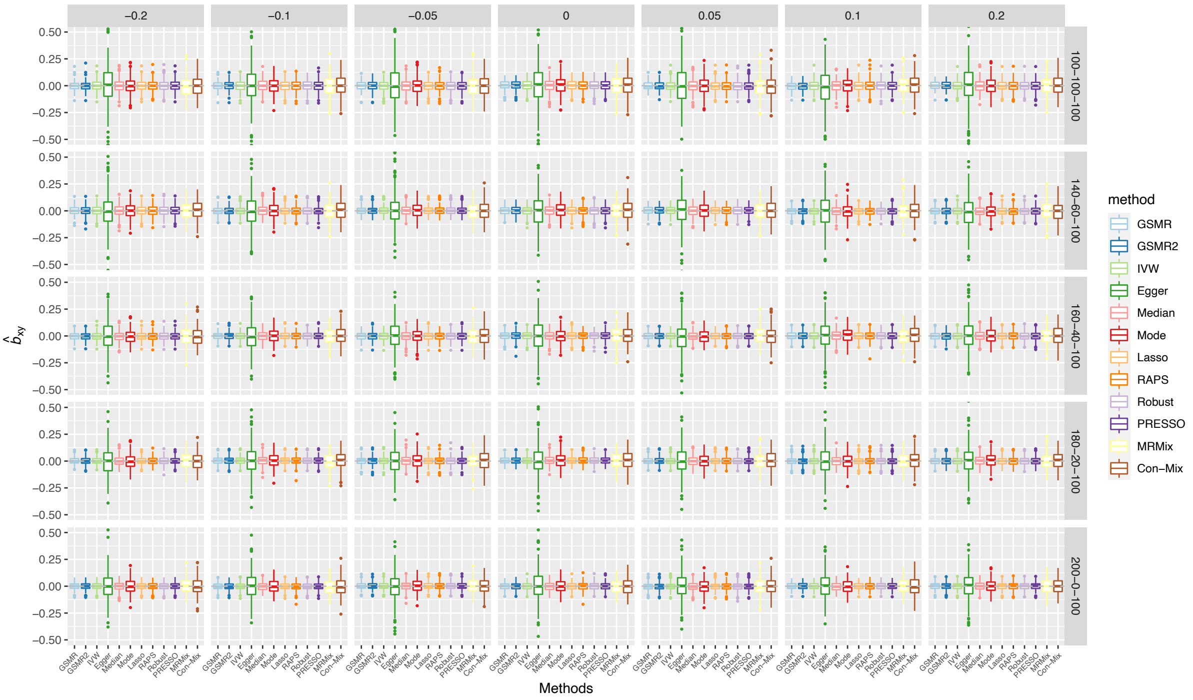


167

$h^2_{zp} = 0.02$

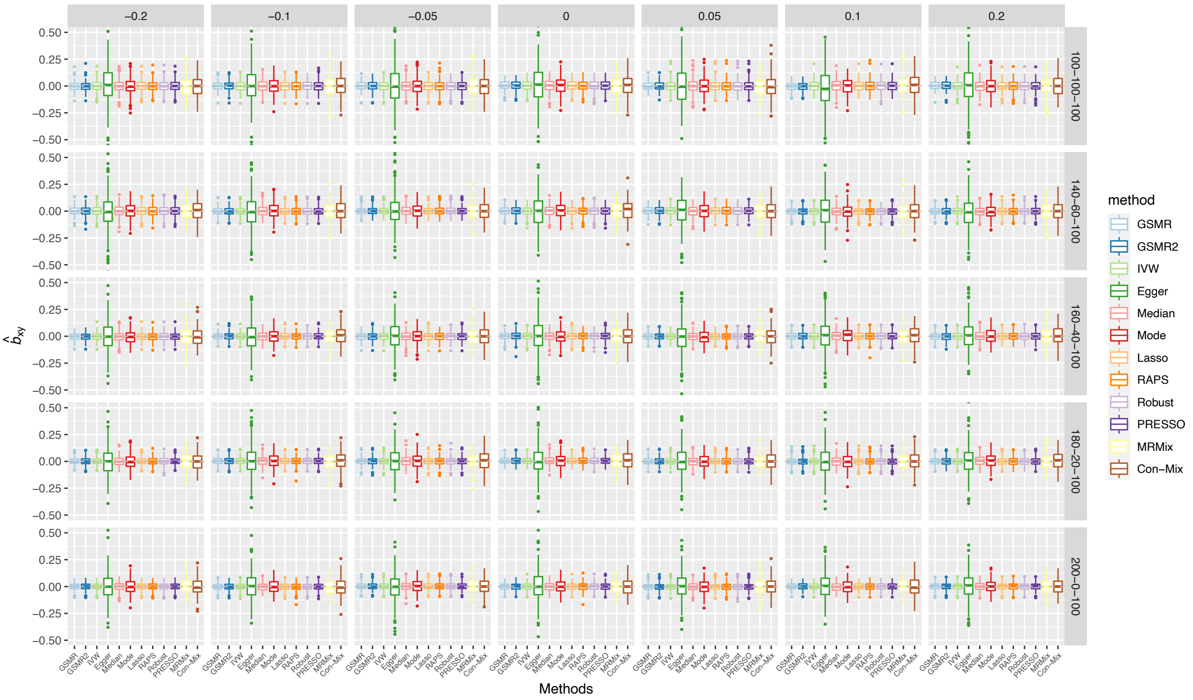


168

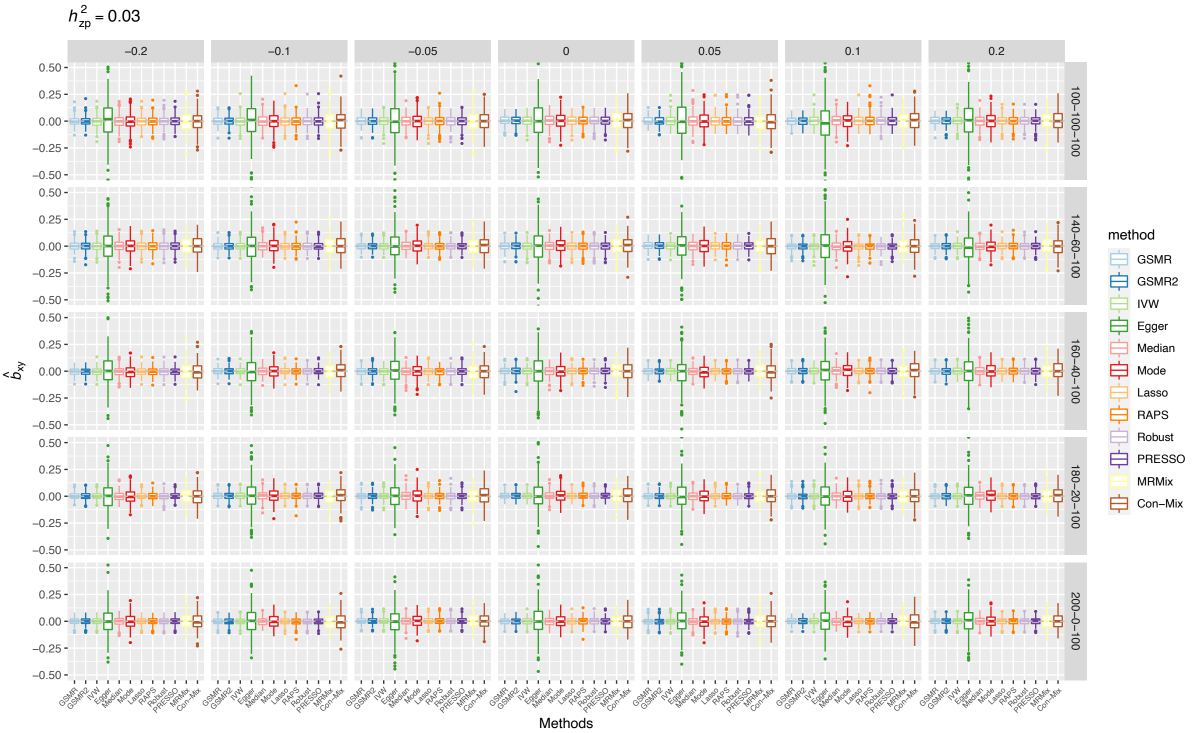

$h^2_{zp} = 0.03$
−0.2
−0.1
−0.05
0
0.05
0.1
0.2
100−100−100
140−60−100
160−40−100
180−20−100
200−0−100
$\hat{b}_{xy}$
Methods
method
GSMR
GSMR2
IVW
Egger
Median
Mode
Lasso
RAPS
Robust
PRESSO
MRMix
Con−Mix


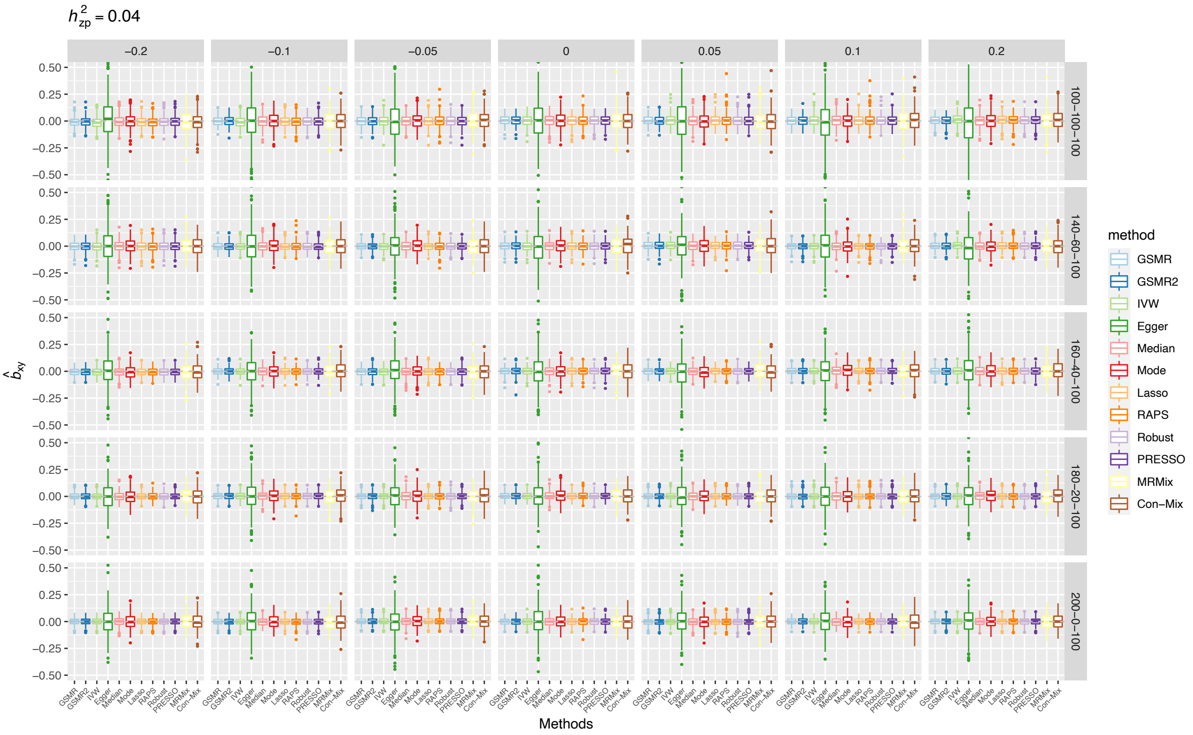

$h^2_{zp} = 0.04$
−0.2
−0.1
−0.05
0
0.05
0.1
0.2
100−100−100
140−60−100
160−40−100
180−20−100
200−0−100
$\hat{b}_{xy}$
Methods
method
GSMR
GSMR2
IVW
Egger
Median
Mode
Lasso
RAPS
Robust
PRESSO
MRMix
Con−Mix

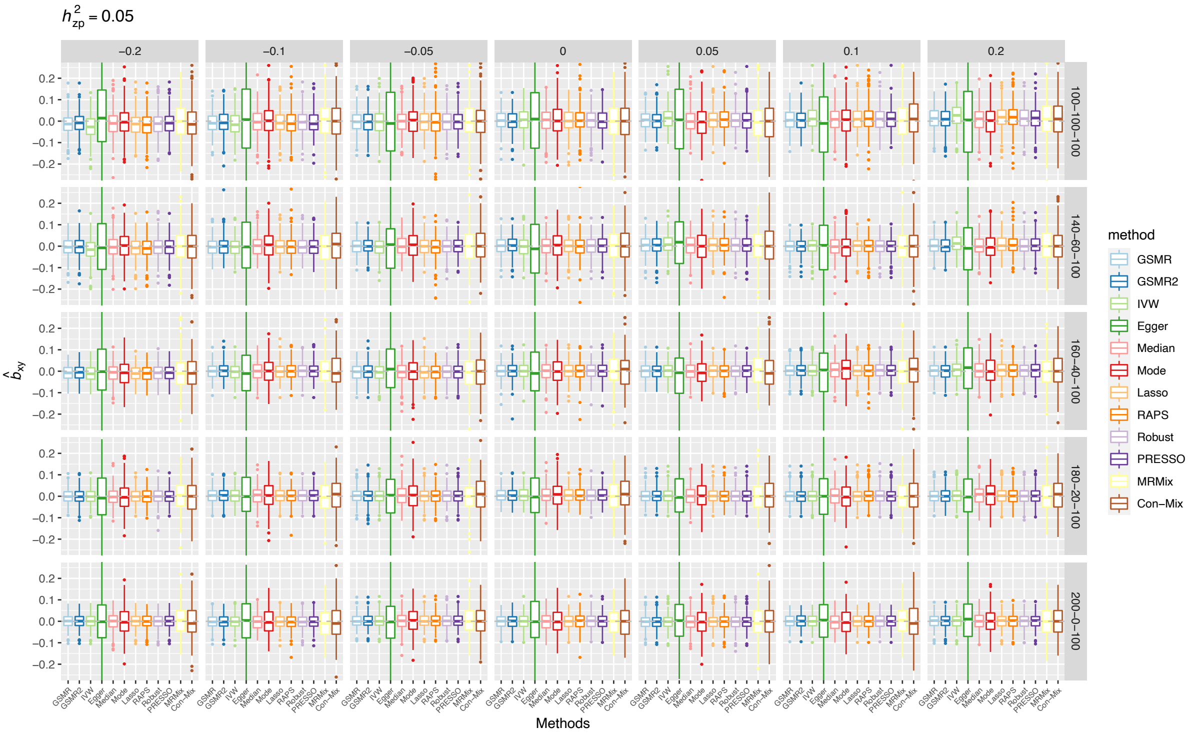


**Supplementary Figure 2. Comparison of the estimate of causal effect between the MR methods in simulations under the null model**. There are five panels in total, each of which indicates a different level of variance explained by the pleiotropic variants (i.e., $h_{zp}^2 \in \{0.01, 0.02, 0.03, 0.04, 0.05\}$) annotated on the top left of each plot. In each panel, there are 35 facets of box plots matching with different levels of pleiotropy and number of causal variants. The column facets indicate 7 different parameters for balanced or directional pleiotropy ($cov(b_{zpx}, b_{zpy})$). The row facets indicate 5 different combinations of numbers of causal and pleiotropic variants ($m_x - m_p - m_y$). The x-axis and colour of the bar denote different MR methods. The y-axis indicates the $b_{\mathrm{xy}}$ estimates. The grey dashed line indicates $b_{\mathrm{xy}}$ equals to 0.

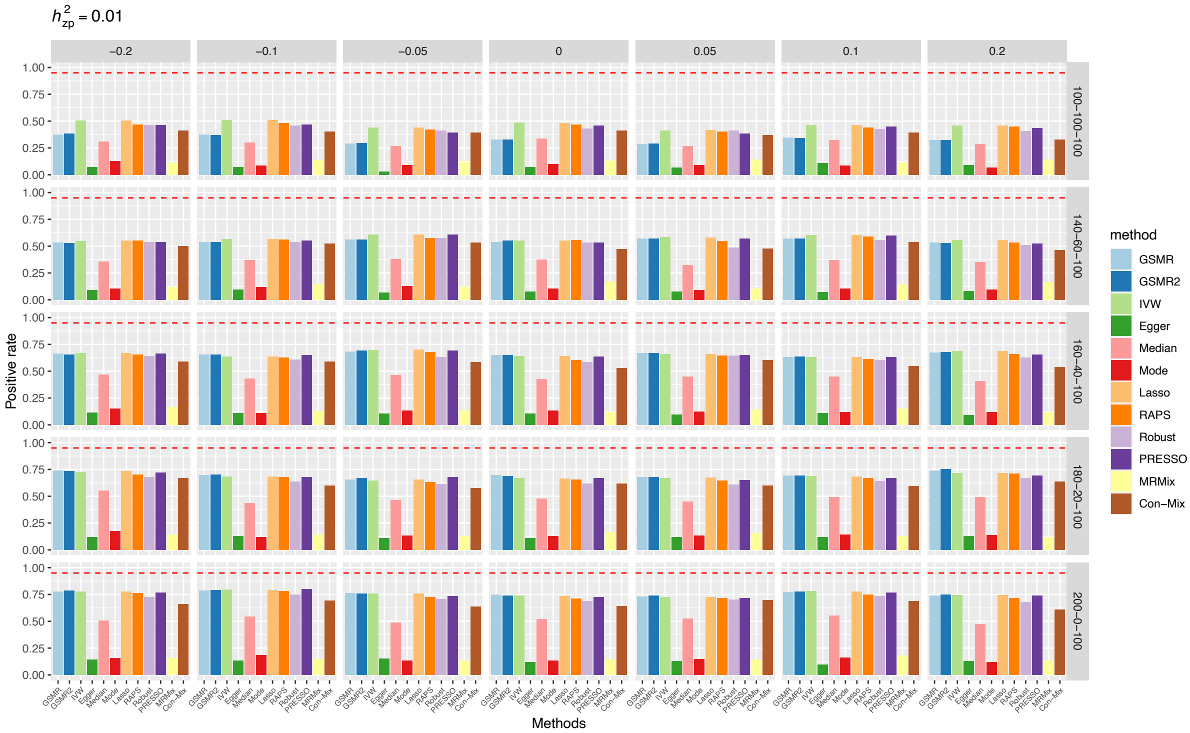
$h^2_{zp} = 0.01$
−0.2
−0.1
−0.05
0
0.05
0.1
0.2
100−100−100
140−60−100
160−40−100
180−20−100
200−0−100
Positive rate
Methods
method
GSMR
GSMR2
IVW
Egger
Median
Mode
Lasso
RAPS
Robust
PRESSO
MRMix
Con−Mix

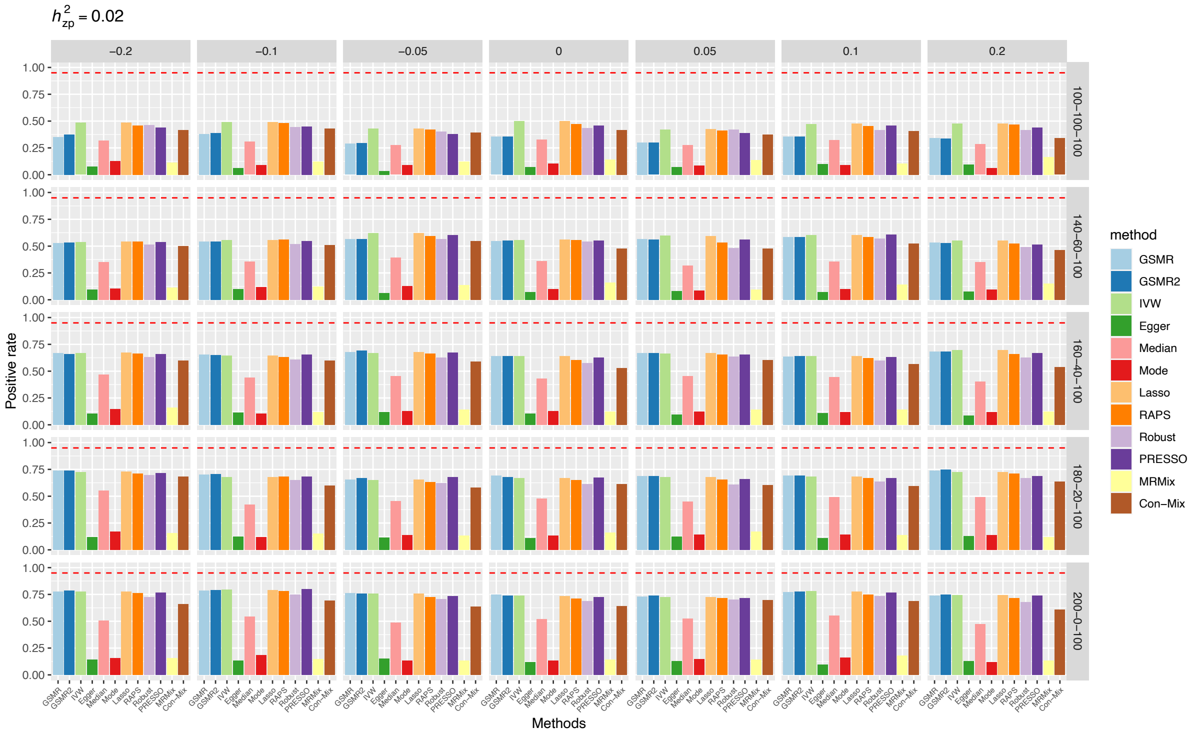
$h^2_{zp} = 0.02$
−0.2
−0.1
−0.05
0
0.05
0.1
0.2
100−100−100
140−60−100
160−40−100
180−20−100
200−0−100
Positive rate
Methods
method
GSMR
GSMR2
IVW
Egger
Median
Mode
Lasso
RAPS
Robust
PRESSO
MRMix
Con−Mix

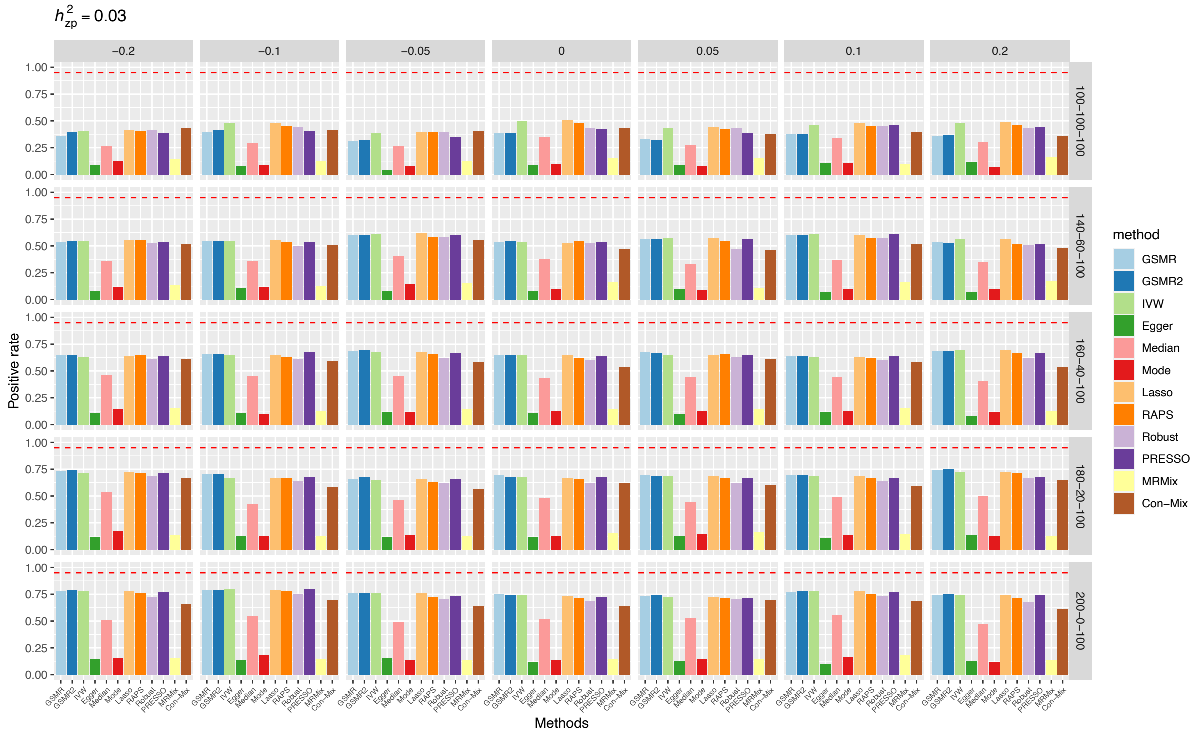
$h^2_{zp} = 0.03$
−0.2
−0.1
−0.05
0
0.05
0.1
0.2
100−100−100
140−60−100
160−40−100
180−20−100
200−0−100
Positive rate
Methods
GSMR
GSMR2
IVW
Egger
Median
Mode
Lasso
RAPS
Robust
PRESSO
MRMix
Con−Mix
method

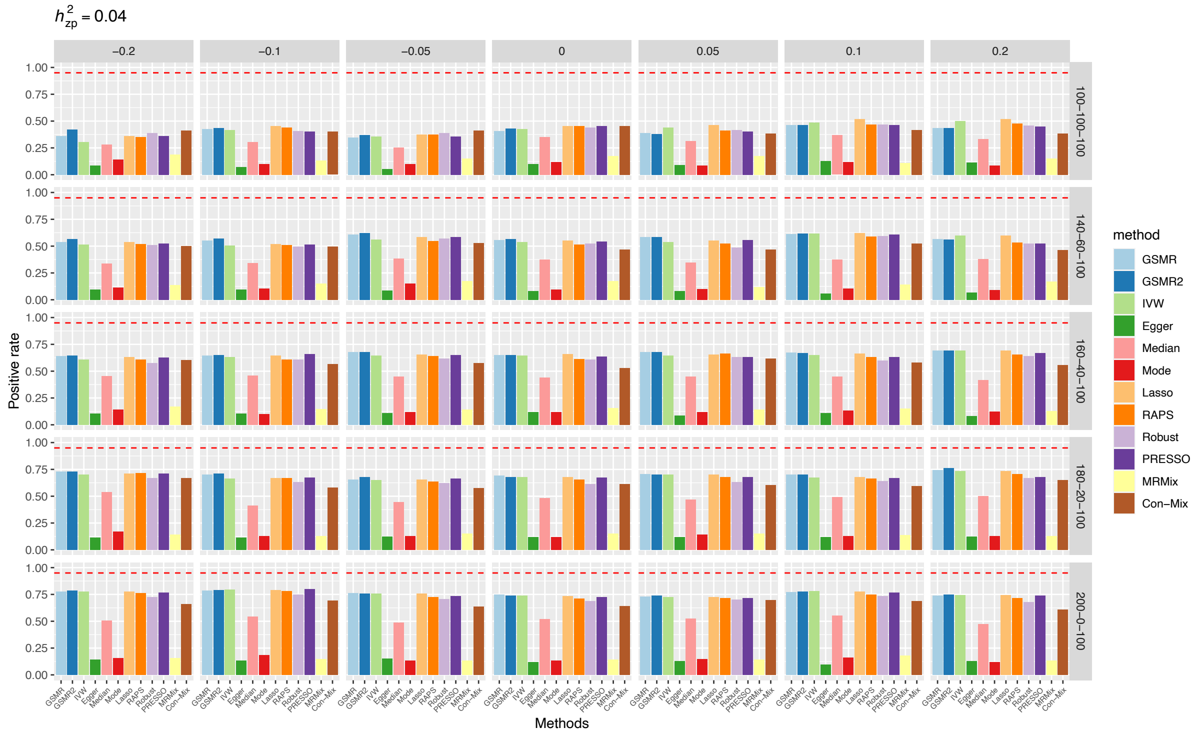
$h^2_{zp} = 0.04$
−0.2
−0.1
−0.05
0
0.05
0.1
0.2
100−100−100
140−60−100
160−40−100
180−20−100
200−0−100
Positive rate
Methods
GSMR
GSMR2
IVW
Egger
Median
Mode
Lasso
RAPS
Robust
PRESSO
MRMix
Con−Mix
method

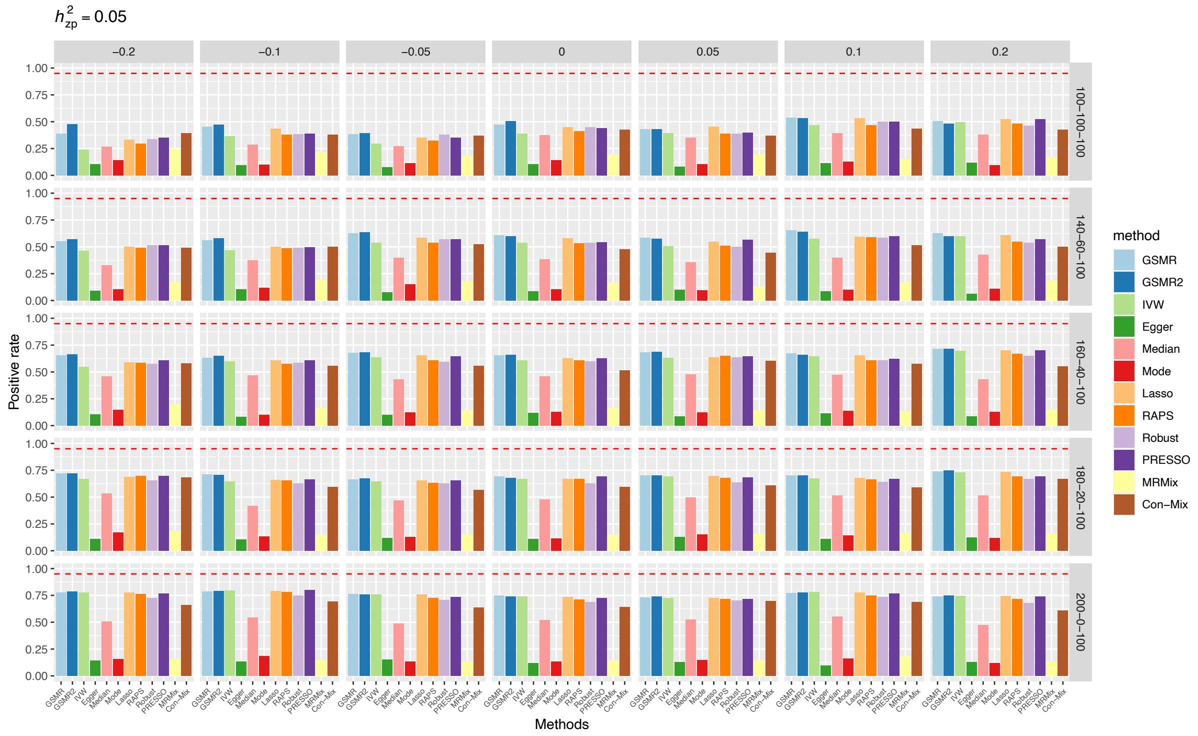


**Supplementary Figure 3. Comparison of power between the MR methods by simulation under the causal model**. There are five panels in total, each of which indicates a different level of variance explained by the pleiotropic variants (i.e., $h^2_{zp} \in \{0.01, 0.02, 0.03, 0.04, 0.05\}$) annotated on the top left of each plot. In each panel, there are 35 facets of bar plots matching with different pleiotropy and number of causal variants. The column facet group indicates 7 different levels for balanced or directional pleiotropy ($cov(b_{zpx}, b_{zpy})$). The row facets indicate 5 different combinations of numbers of causal and pleiotropic variants ($m_x - m_p - m_y$). The x-axis and colour of the bar denote different MR methods. The y-axis indicates the power (true positive rate). The red dashed line indicates power equals to 0.95.

198
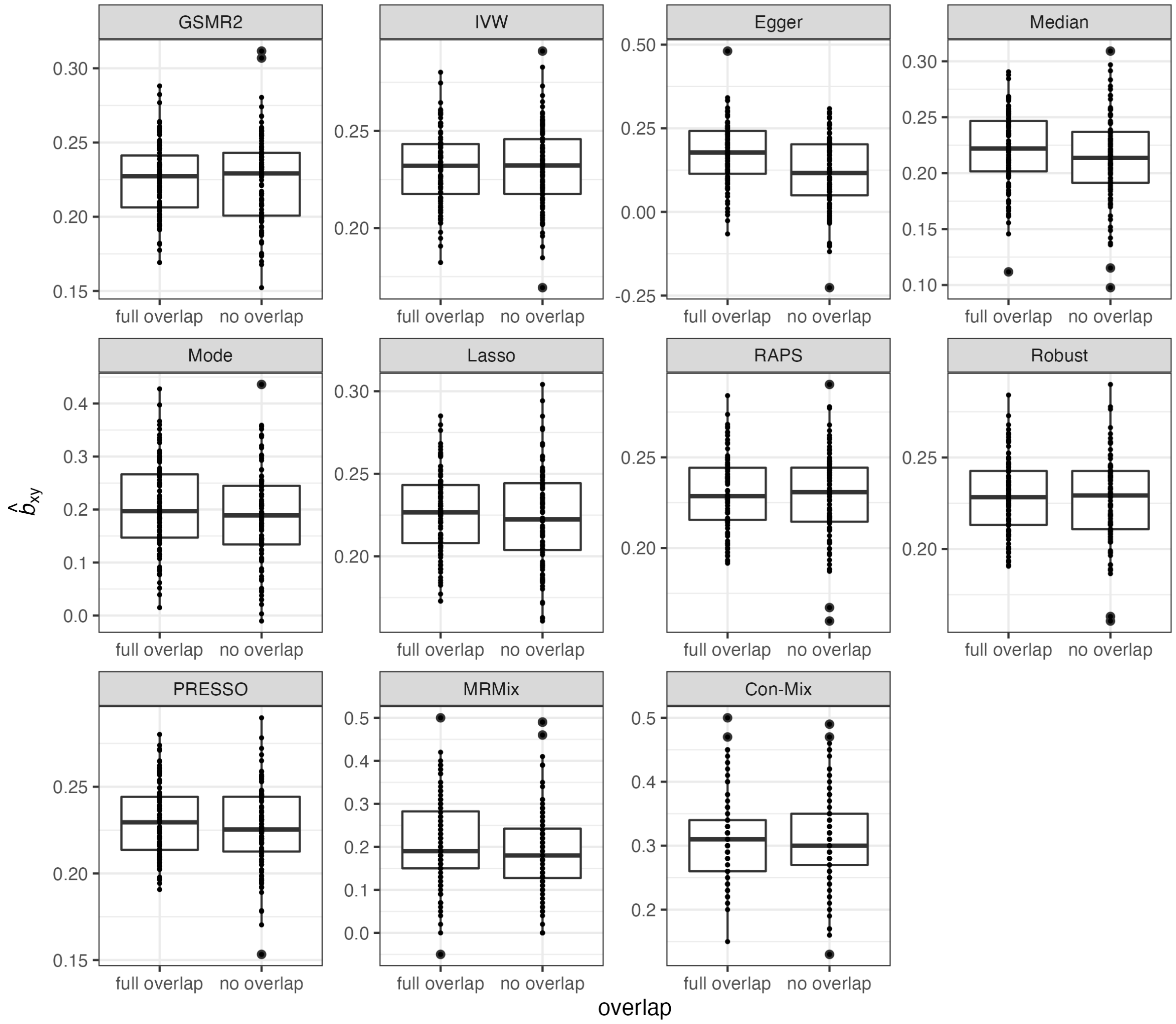
GSMR2
IVW
Egger
Median
Mode
Lasso
RAPS
Robust
PRESSO
MRMix
Con-Mix
full overlap
no overlap
$\hat{b}_{xy}$
overlap

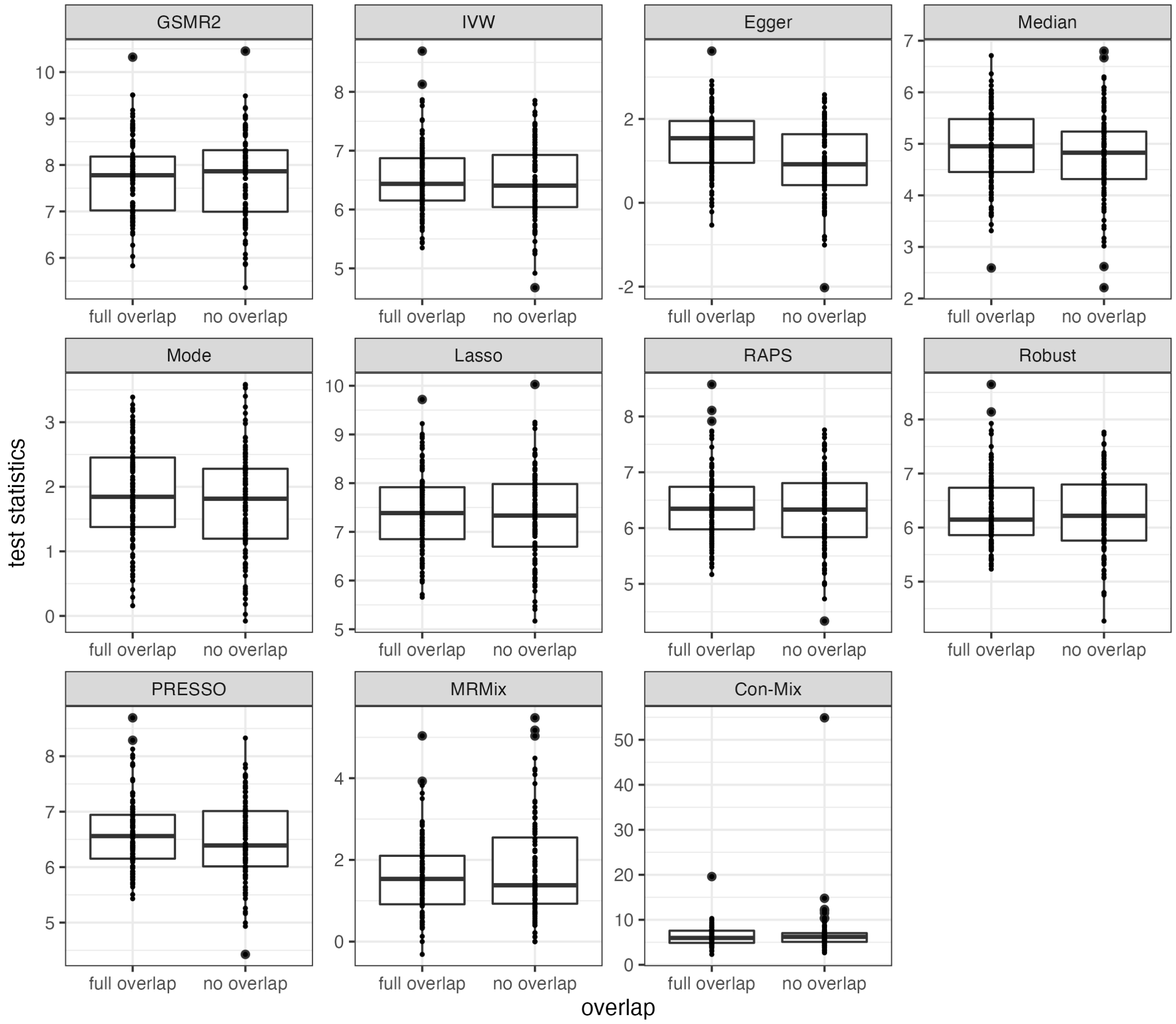


**Supplementary Figure 4. Comparison of MR causal estimates given full or no sample overlap.** The upper boxplot for $b_{xy}$ estimates given full or no sample overlap between simulated exposure and outcome. The lower boxplot shows the comparison of the test statistics (i.e., z-scores). Each scenario has 100 data points, representing 100 replications. Each panel corresponds one of the 11 MR methods. The true $b_{xy}$ is set as 0.2. Only Egger method shows significant inflation in the comparison of $b_{xy}$ estimate ($p$-value = 3.63E-5) and test statistics ($p$-value = 2.36E-5).

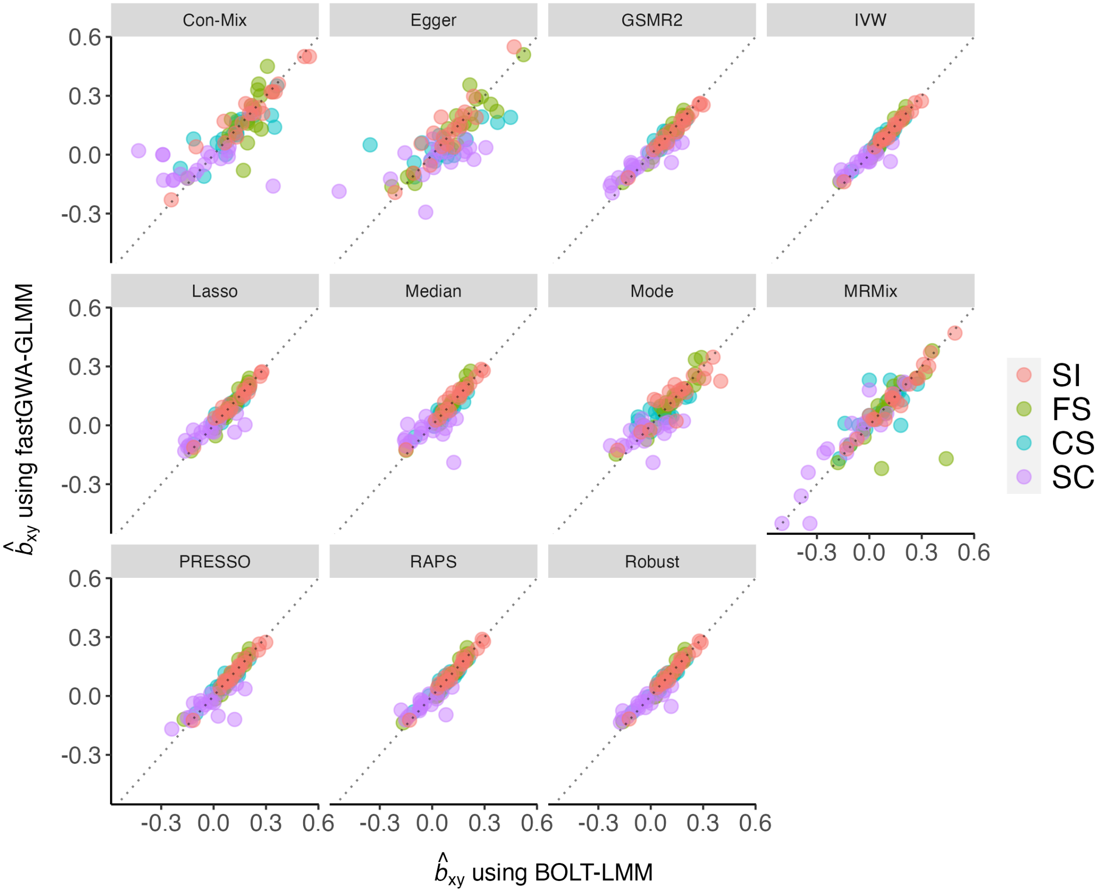


**Supplementary Figure 5. Comparison of estimates of causal effects when using GWAS obtained by LMM + transformation or GLMM.** This figure shows the comparison of $b_{xy}$ estimates using the smoking related GWAS results obtained by BOLT-LMM + LMOR transformation or fastGWA-GLMM. Each panel indicates the MR method used for estimation. The color of each dot indicates the smoking traits. The grey dashed line is the diagonal line of the coordinate plane.

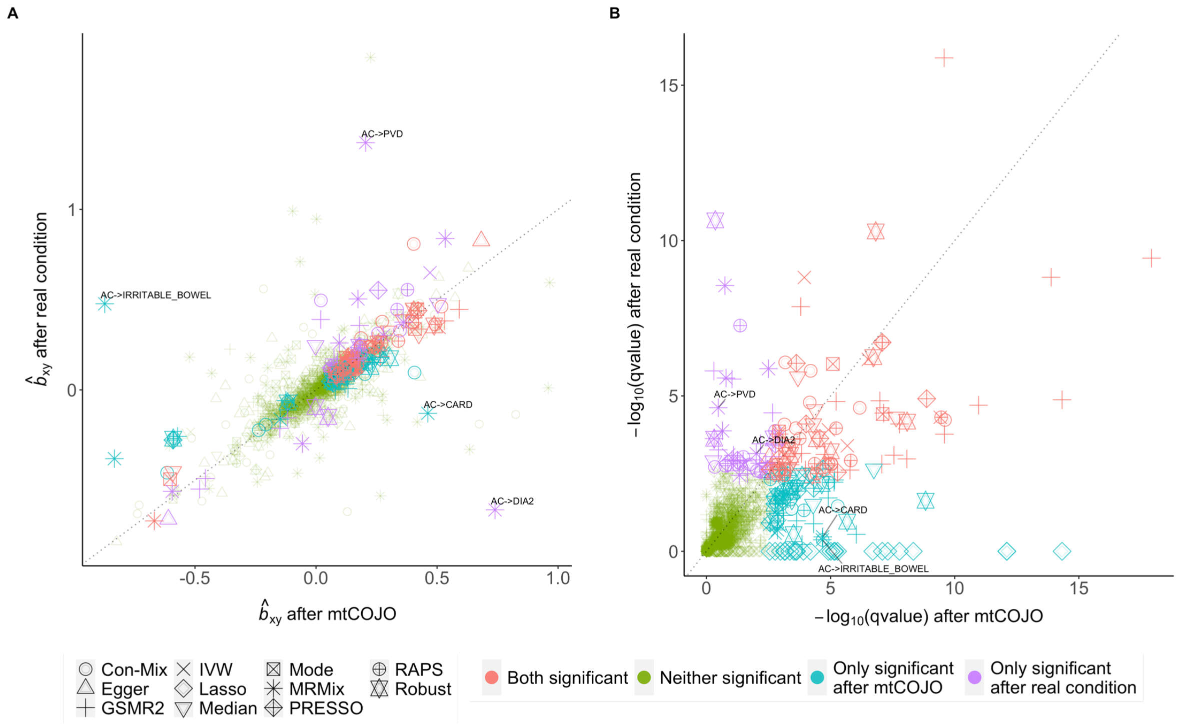


**Supplementary Figure 6. Comparison of MR results using GWAS summary data from mtCOJO with those using GWAS summary data from conditional analysis with individual-level data.** The MR analyses were performed to estimate the causal effects of 7 SUB traits on 18 common diseases. The two scenarios (mtCOJO or individual-level data-based conditional analysis) were tested using the same data. In panel A, the x-axis indicates the $b_{xy}$ estimate from MR analysis using the exposure from mtCOJO analysis, while the y-axis indicates the $b_{xy}$ estimate using exposure from individual-level data-based conditional GWAS analysis with BOLT-LMM. In panel B, the x-axis represents–$\log_{10}$(qvalue) from the MR analysis using exposure GWAS data from mtCOJO analysis, while the y-axis denotes–$\log_{10}$(qvalue) using exposure GWAS data from individual-level data-based conditional GWAS analysis. The colours of the dots represent the significance levels of the MR estimates. Those associations with large difference in MR estimate or significance level are annotated with "exposure->outcome". AC: alcohol consumption. CI: coffee intake. IRRITABLE_BOWEL: irritable bowel syndrome. CARD: cardiovascular disease. DIA2: type 2 diabetes. DYSLIPID: dyslipidemia. OSTIOA: osteoarthritis. OSTIOP: osteoporosis.

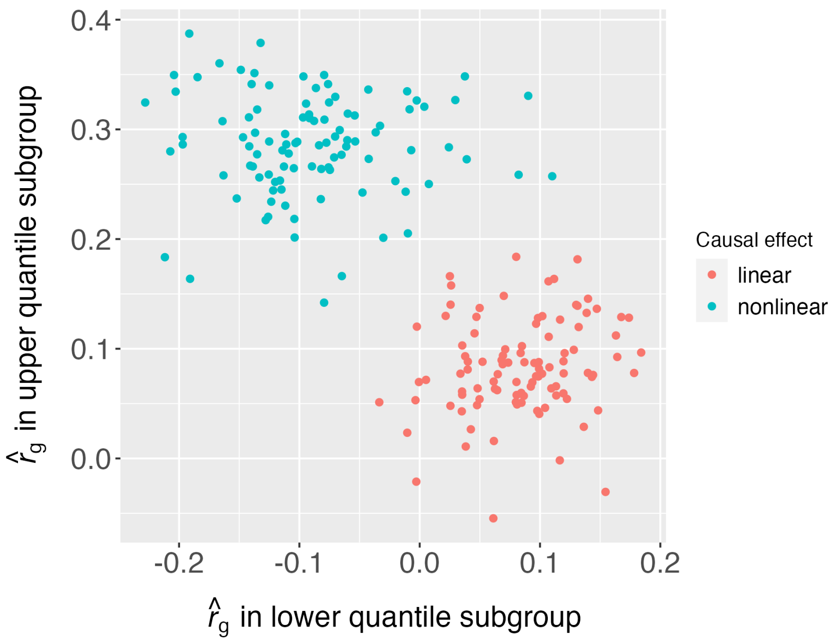


**Supplementary Figure 7. Genetic correlation estimates between exposure and outcome in exposure subgroups.** The x-axis and y-axis indicate the genetic correlation estimates between the outcome and the exposure of the lower and upper quantile subgroup, respectively. Each dot represents a replication in the simulation. The colour denotes whether the simulated causal effect is linear (red) or nonlinear (blue).

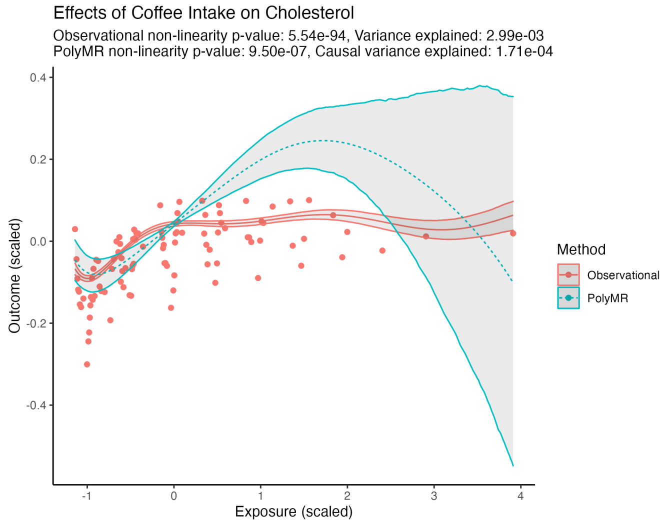


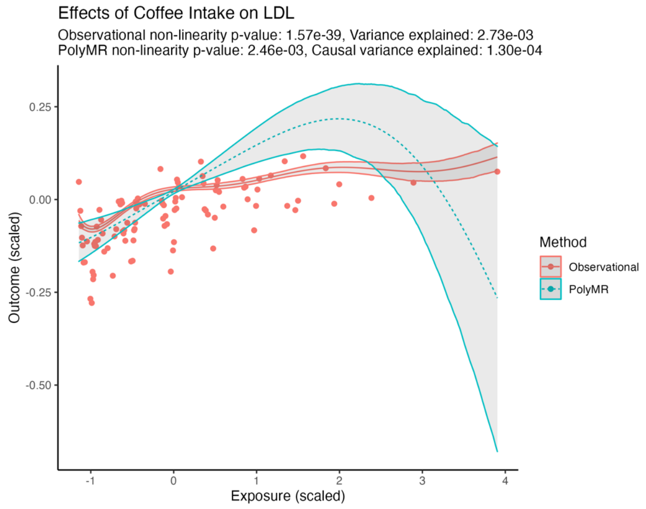


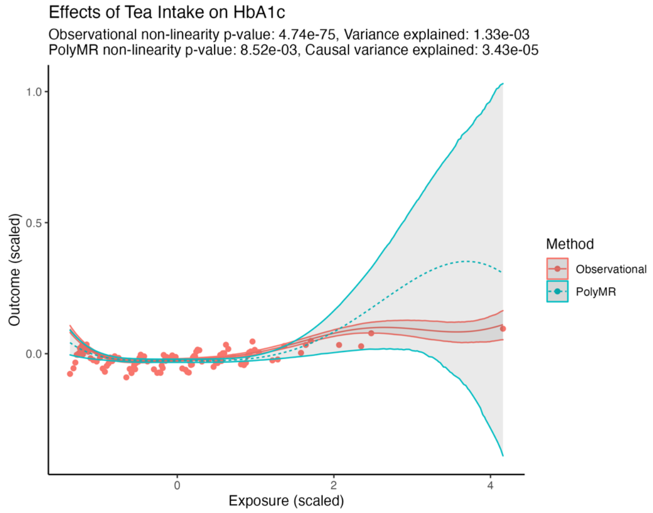


**Supplementary Figure 8**. Significant dose-dependent effects of coffee intake and tea intake on biomarkers. The x-axis and y-axis indicate the scaled phenotypes for exposure and outcome, respectively. The red line represents the multivariable regression line of stratified exposure on outcome. The red dots represent the mean outcome against the median exposure for each of the100 bins. The dashed blue line represents the estimated causal function from PolyMR. The grey area around both lines indicates the 95% confidence interval.

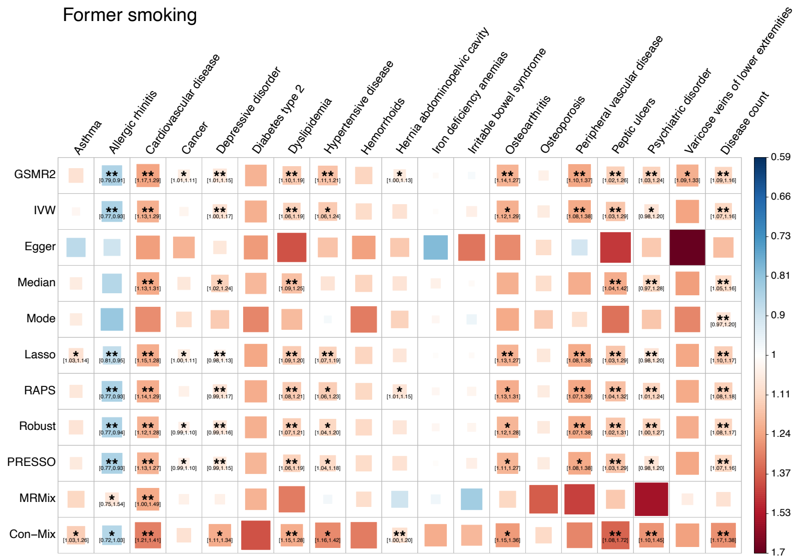


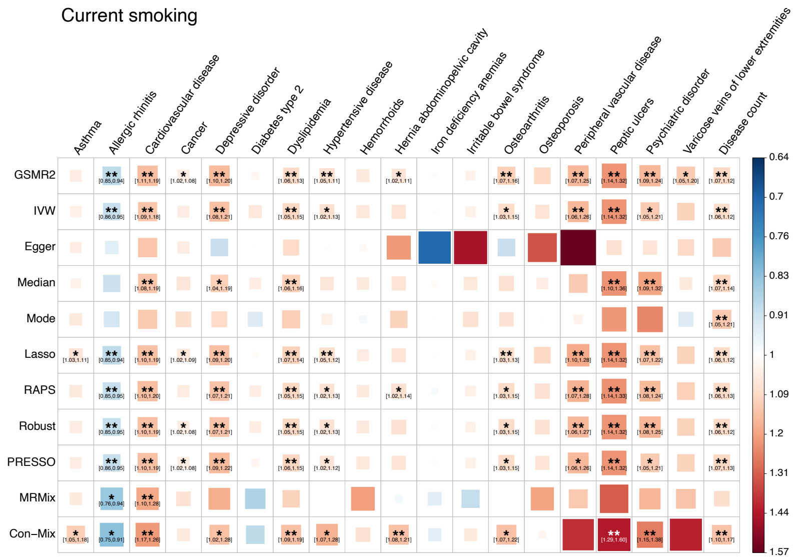


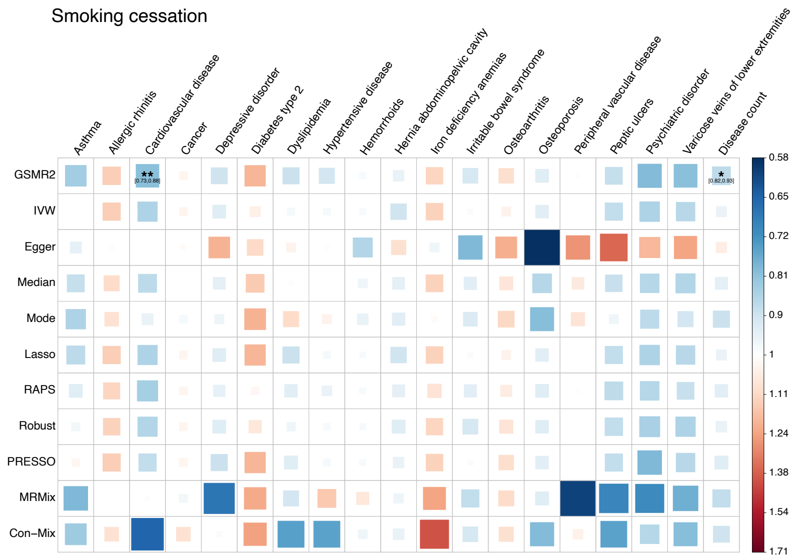


**Supplementary Figure 9. Estimating the causal associations between smoking-related traits and common diseases in the UKB from different MR methods.** The legend is the same as Figures 1-4.

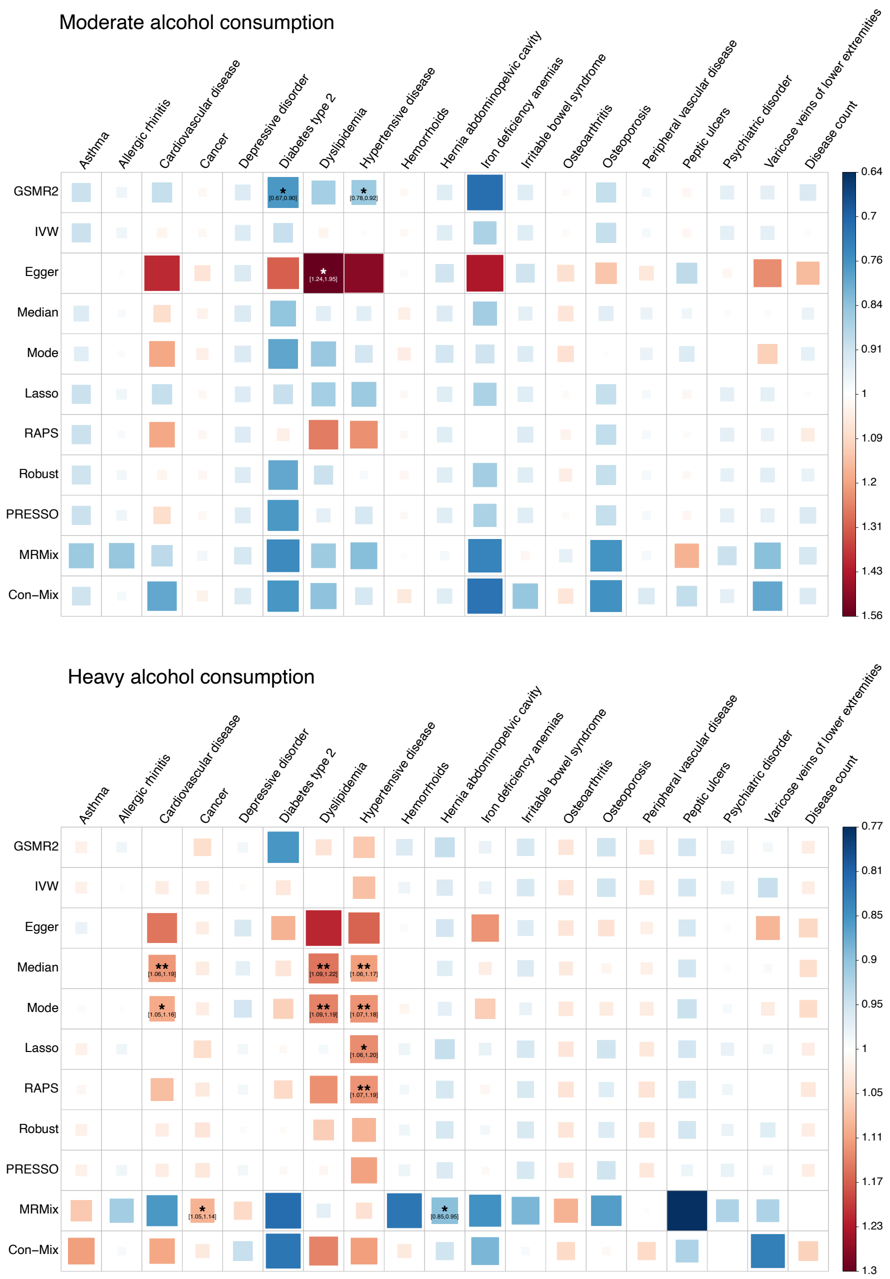


**Supplementary Figure 10. Estimating the causal associations between moderate/heavy alcohol consumption and common diseases in the UKB from different MR methods.** The legend is the same as Figures 1-4.

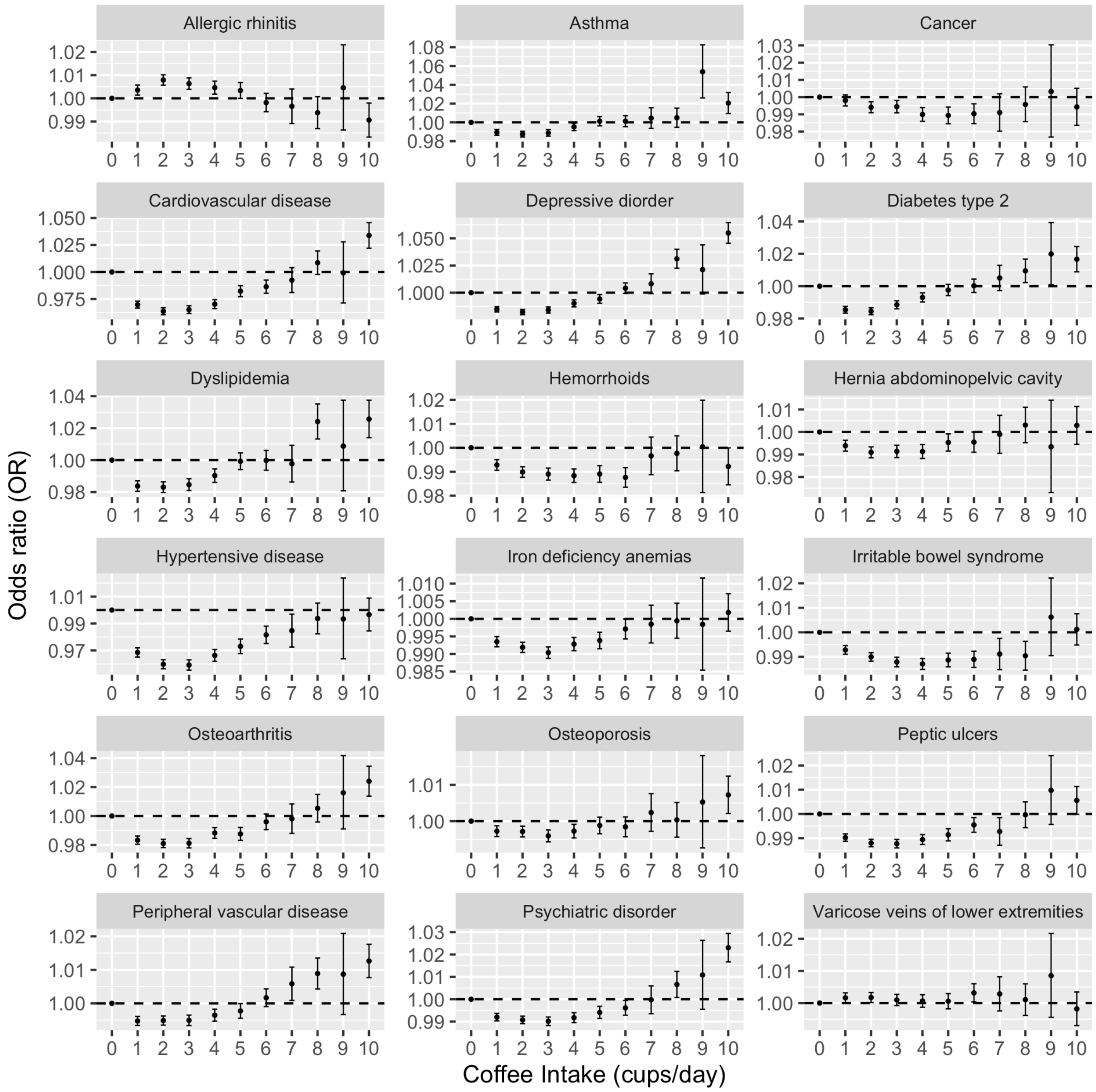


**Supplementary Figure 11. Dosage-dependent effects of coffee intake on common diseases.** Shown are the logistic regression results of common diseases on difference coffee intake level. The x-axis indicates different intake level. Intake large than 10 cups/day is classified into group 10. The y-axis indicates the odds ratio (OR) from the logistic regression. Error bars indicate the 95% confidence interval of each OR estimate.

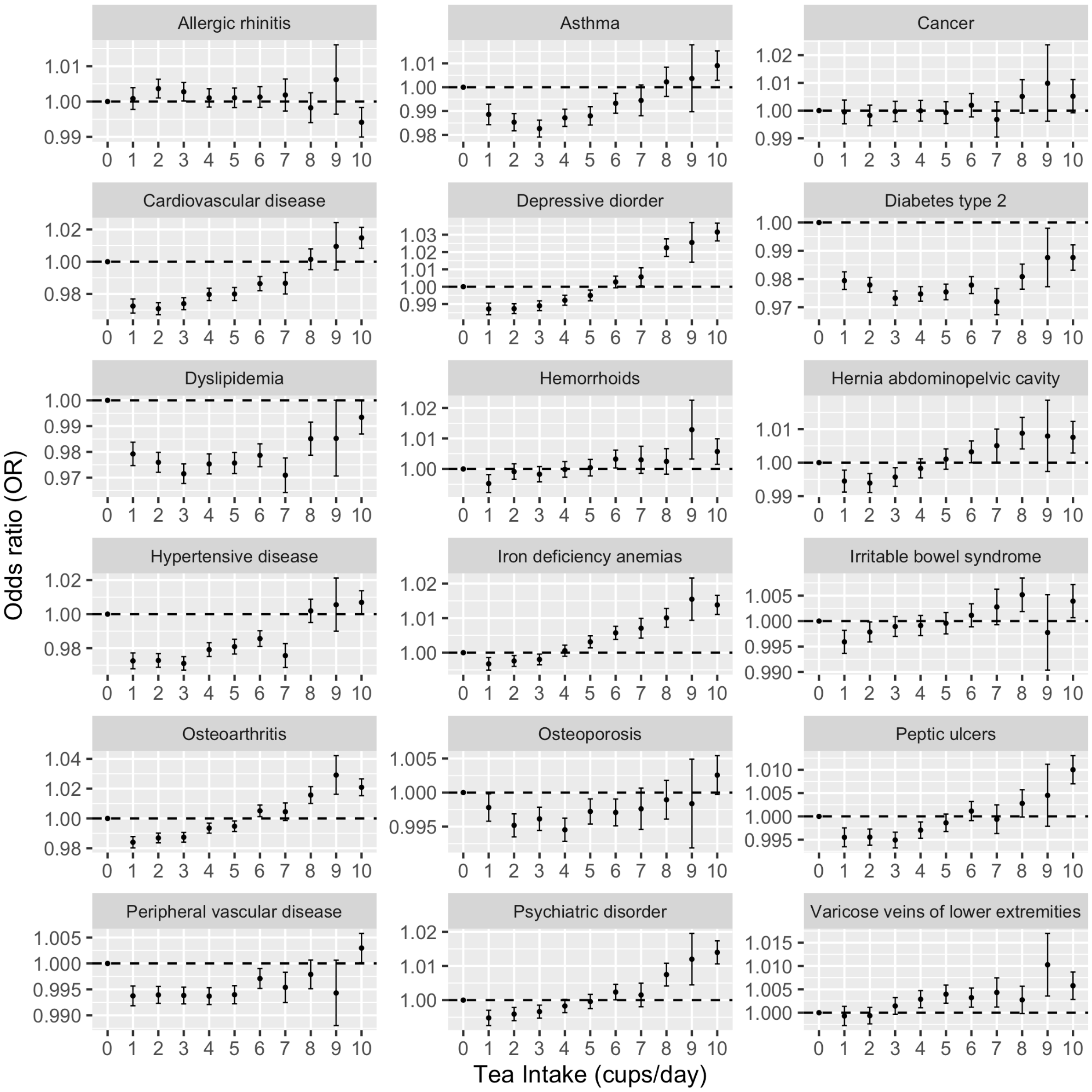


**Supplementary Figure 12. Dosage-dependent effects of tea intake on common diseases.** Shown are the logistic regression of common diseases on different tea intake levels. The x-axis indicates different intake levels. Intake larger than 10 cups/day is classified into group 10. The y-axis indicates the odds ratio (OR) from the logistic regression. Error bars indicate the 95% confidence interval of each OR estimate.

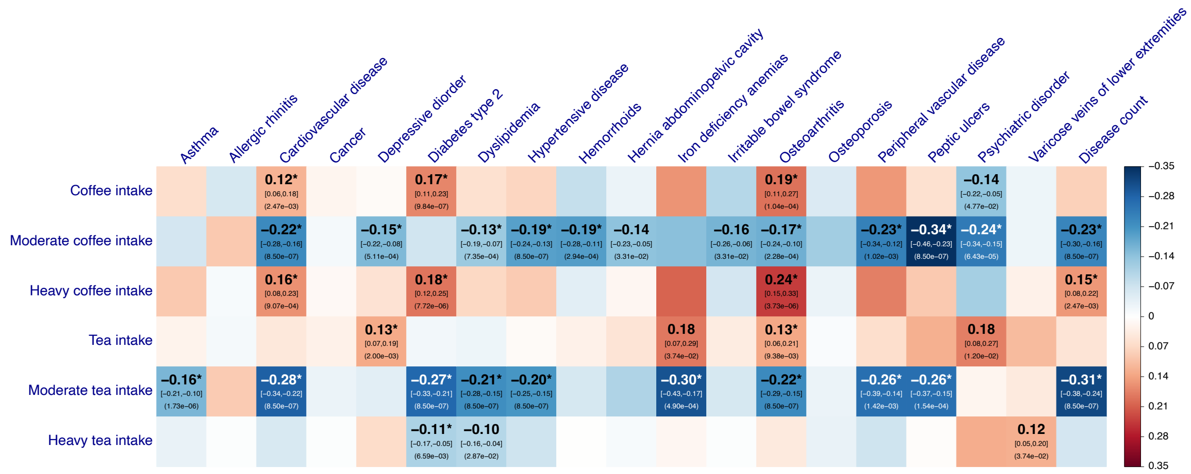


**Supplementary Figure 13. Estimates of genetic correlation between coffee (or tea) intake and common diseases.** The suggestive estimates (local FDR < 0.05) are labelled with $\hat{r}_g$ [95% confidence interval] (FDR), and the significant estimates (local FDR < 0.01) are labelled with an additional asterisk "*".

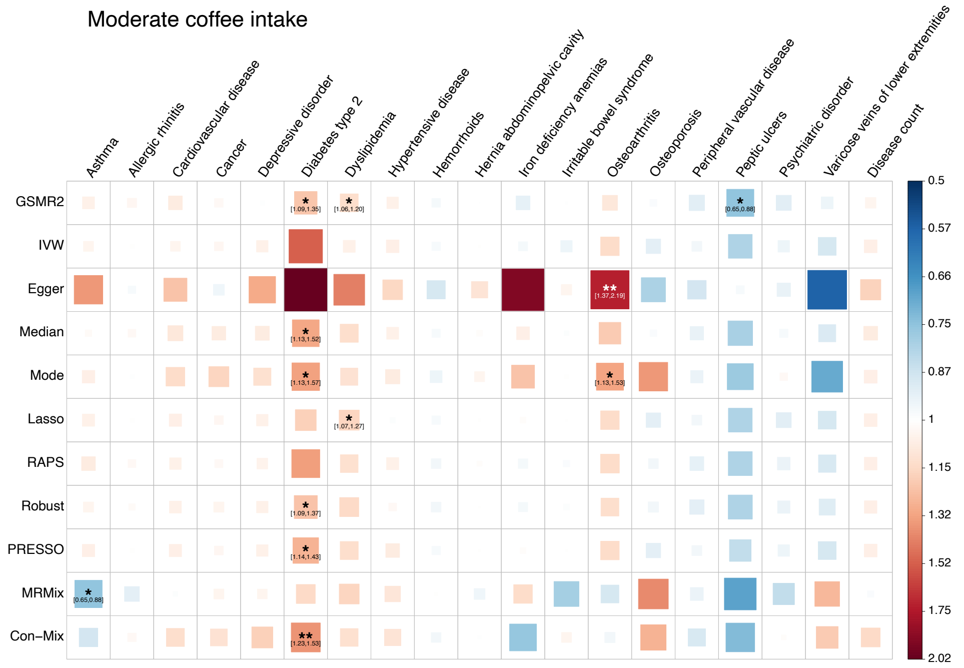


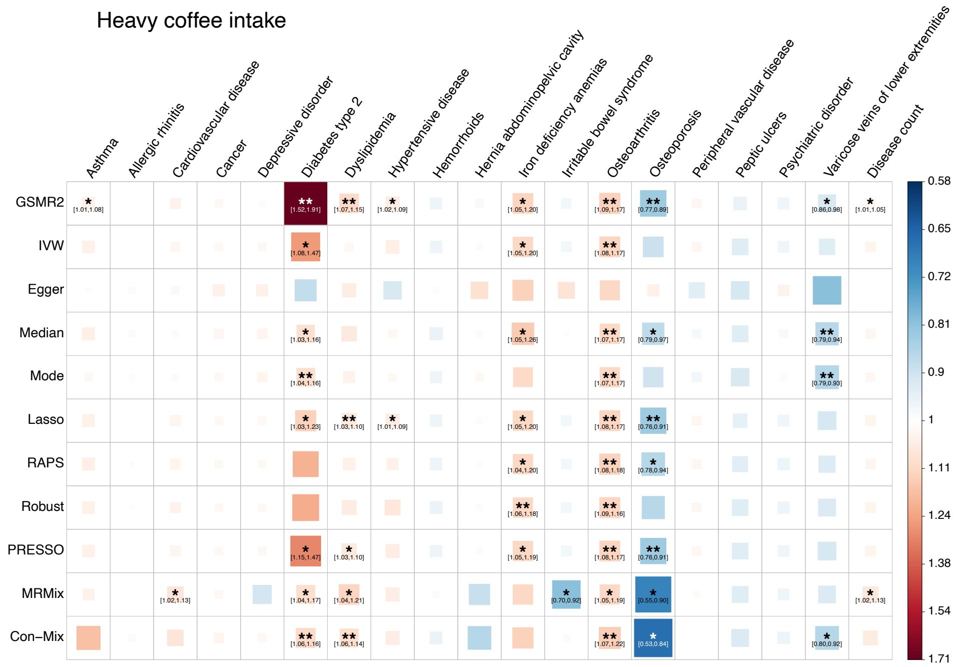


**Supplementary Figure 14. Estimating the causal associations between moderate/heavy coffee intake and common diseases in the UKB.** The legend is the same as Figures 1-4.

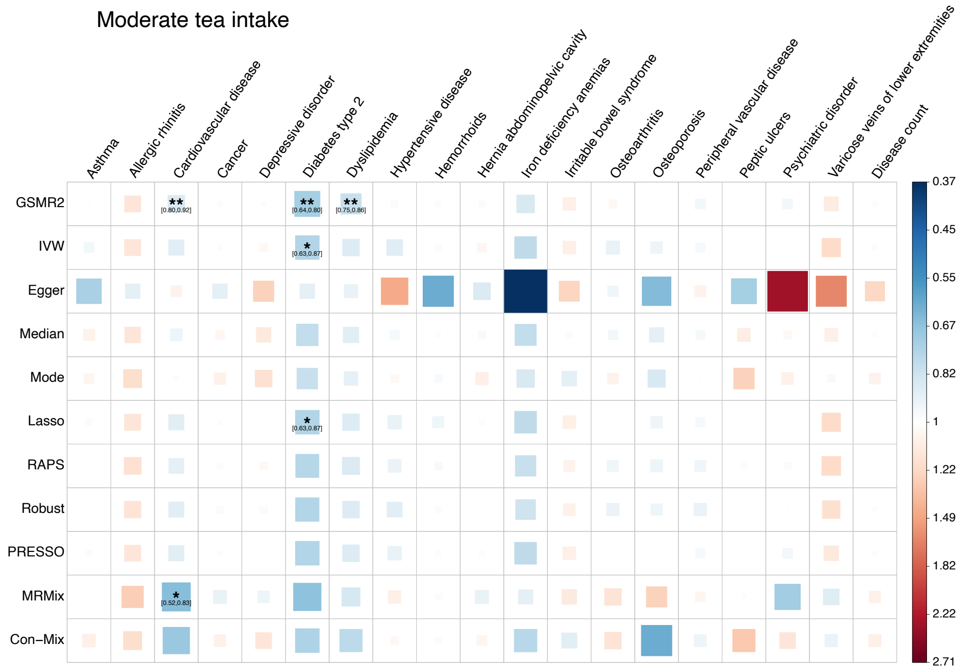


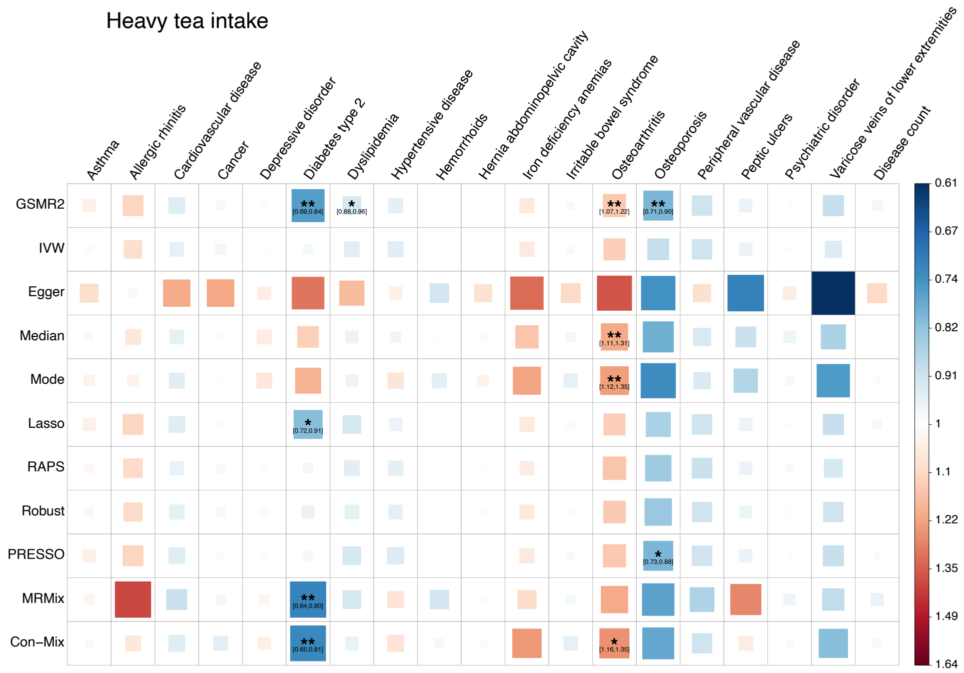


**Supplementary Figure 15. Estimating the causal associations between moderate/heavy tea intake and common diseases in the UKB.** The legend is the same as Figures 1-4.

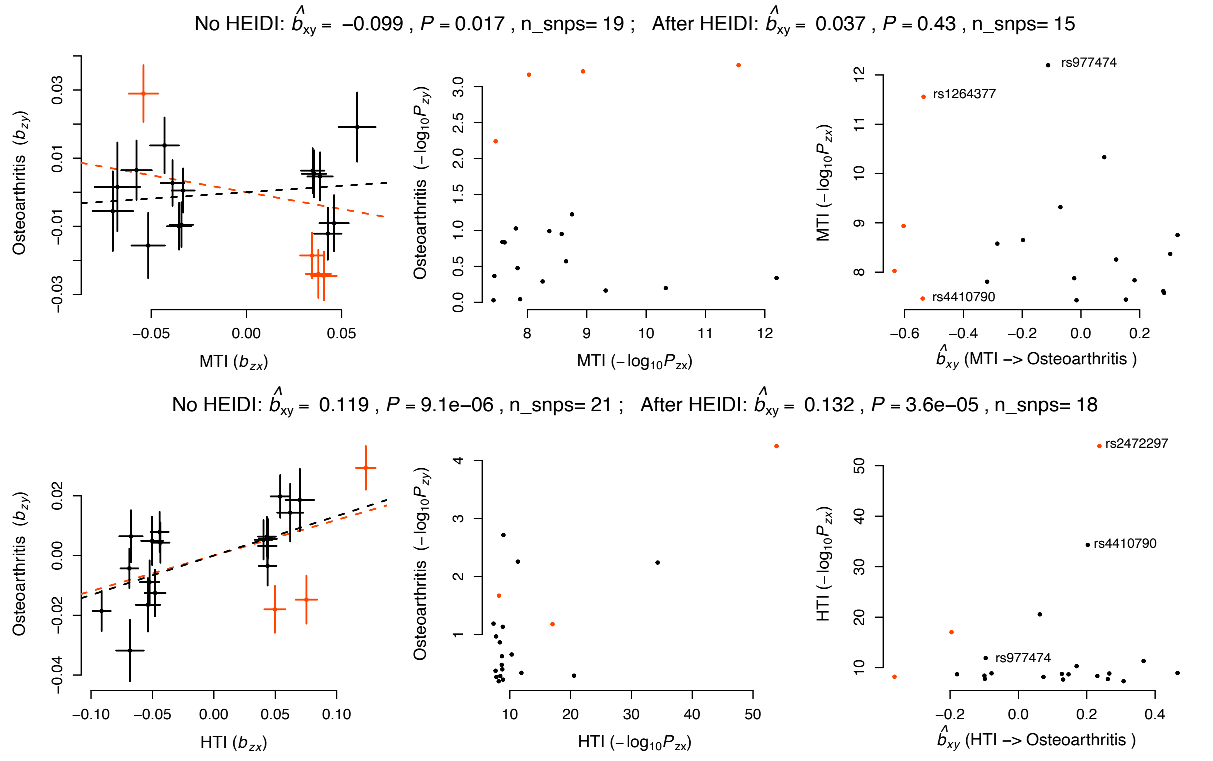


**Supplementary Figure 16. Causal effect of MTI or HTI on osteoarthritis estimated at individual IVs.** The upper and lower layers are for MTI and HTI, respectively. The subtitle on the top of each layer shows the causal estimate before and after the HEIDI-outlier filtering. The pleiotropic IVs detected by the HEIDI-outlier test are highlighted in red. In each layer, the left column shows the SNP effect estimates for the exposure (x-axis) plotted against those for the outcome (y-axis). Each error bar indicates the *s.e.* of the SNP effect estimate. The slope of the red and black dashed line indicates $\hat{b}_{xy}$ before and after HEIDI-outlier filtering, respectively. The middle column shows the -log$_{10}$($P$) of each IV for exposure (x-axis) against that for outcome (y-axis). The right column shows $\hat{b}_{xy}$ for each IV (x-axis) against the corresponding GWAS -log$_{10}$($P$) for the exposure (y-axis). MTI: moderate tea intake. HTI: heavy tea intake. The top two signals in TI/HTI (rs4410790 and rs2472297) and top two in MTI (rs1264377 and rs977474) were annotated. The rs2472297 was not significant in MTI and rs1264377 was not significant in HTI.

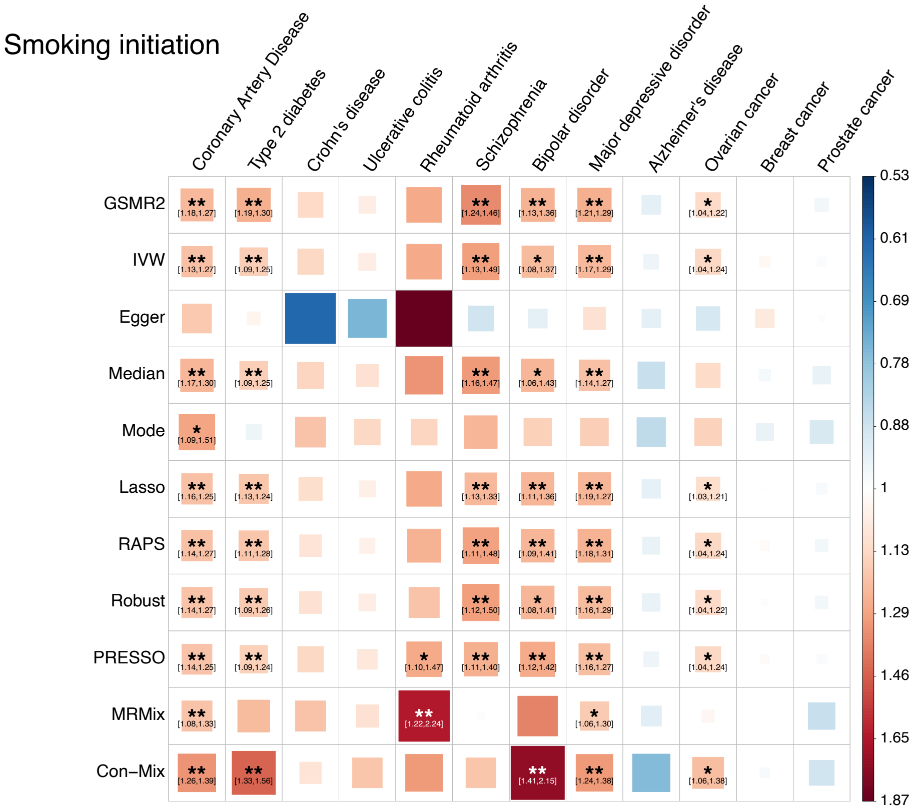
Smoking initiation
Coronary Artery Disease
Type 2 diabetes
Crohn's disease
Ulcerative colitis
Rheumatoid arthritis
Schizophrenia
Bipolar disorder
Major depressive disorder
Alzheimer's disease
Ovarian cancer
Breast cancer
Prostate cancer
GSMR2
IVW
Egger
Median
Mode
Lasso
RAPS
Robust
PRESSO
MRMix
Con−Mix
0.53
0.61
0.69
0.78
0.88
1
1.13
1.29
1.46
1.65
1.87

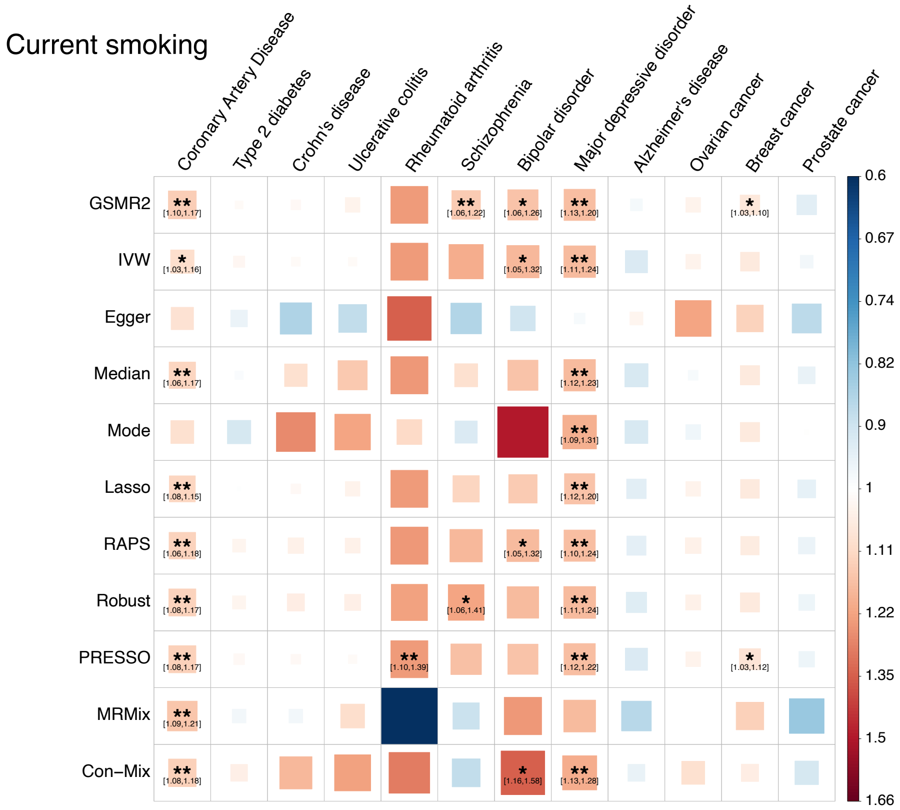
Current smoking
Coronary Artery Disease
Type 2 diabetes
Crohn's disease
Ulcerative colitis
Rheumatoid arthritis
Schizophrenia
Bipolar disorder
Major depressive disorder
Alzheimer's disease
Ovarian cancer
Breast cancer
Prostate cancer
GSMR2
IVW
Egger
Median
Mode
Lasso
RAPS
Robust
PRESSO
MRMix
Con−Mix
0.6
0.67
0.74
0.82
0.9
1
1.11
1.22
1.35
1.5
1.66

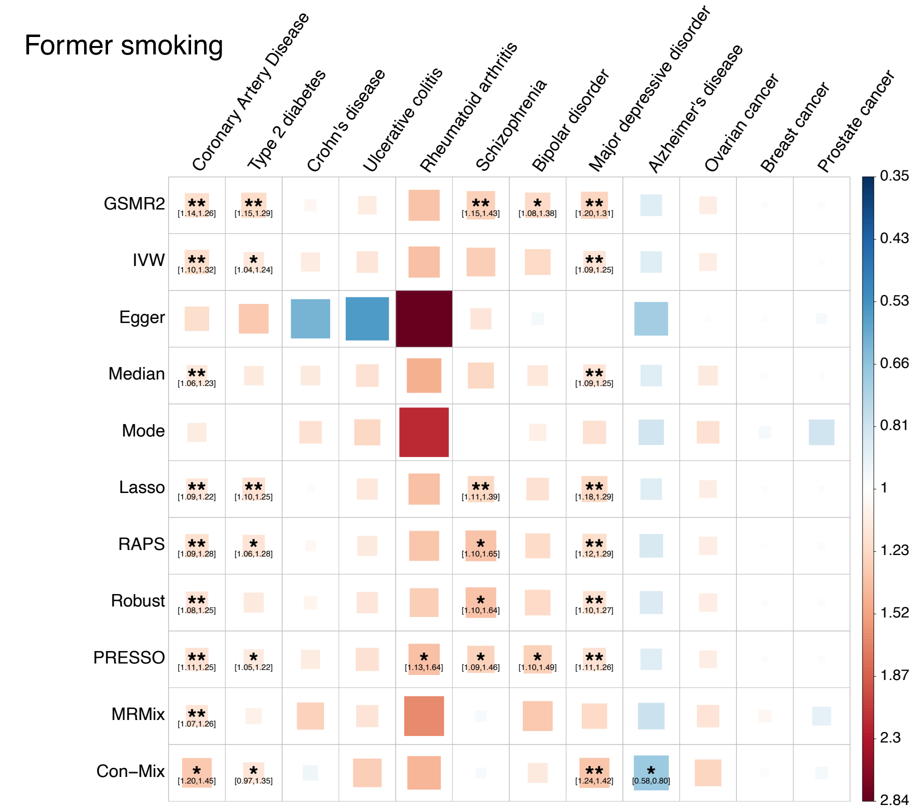

Former smoking
Coronary Artery Disease
Type 2 diabetes
Crohn's disease
Ulcerative colitis
Rheumatoid arthritis
Schizophrenia
Bipolar disorder
Major depressive disorder
Alzheimer's disease
Ovarian cancer
Breast cancer
Prostate cancer
GSMR2
IVW
Egger
Median
Mode
Lasso
RAPS
Robust
PRESSO
MRMix
Con−Mix
0.35
0.43
0.53
0.66
0.81
1
1.23
1.52
1.87
2.3
2.84


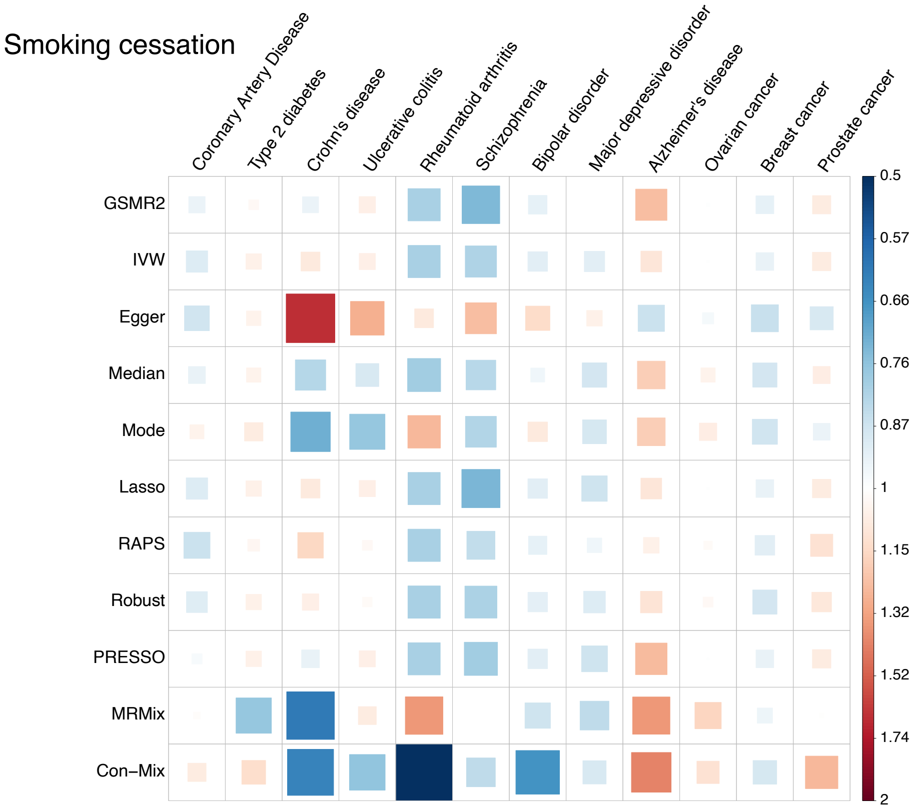

Smoking cessation
Coronary Artery Disease
Type 2 diabetes
Crohn's disease
Ulcerative colitis
Rheumatoid arthritis
Schizophrenia
Bipolar disorder
Major depressive disorder
Alzheimer's disease
Ovarian cancer
Breast cancer
Prostate cancer
GSMR2
IVW
Egger
Median
Mode
Lasso
RAPS
Robust
PRESSO
MRMix
Con−Mix
0.5
0.57
0.66
0.76
0.87
1
1.15
1.32
1.52
1.74
2

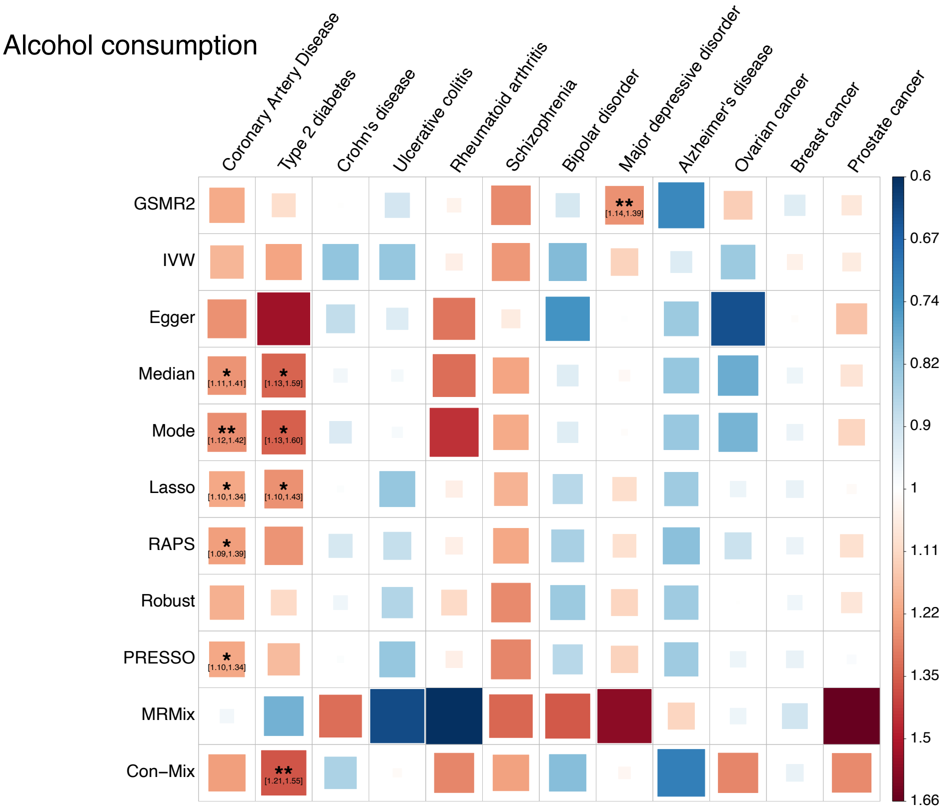

Alcohol consumption
Coronary Artery Disease
Type 2 diabetes
Crohn's disease
Ulcerative colitis
Rheumatoid arthritis
Schizophrenia
Bipolar disorder
Major depressive disorder
Alzheimer's disease
Ovarian cancer
Breast cancer
Prostate cancer
GSMR2
IVW
Egger
Median
Mode
Lasso
RAPS
Robust
PRESSO
MRMix
Con−Mix
** [1.14,1.39]
* [1.11,1.41]
* [1.13,1.59]
** [1.12,1.42]
* [1.13,1.60]
* [1.10,1.34]
* [1.10,1.43]
* [1.09,1.39]
* [1.10,1.34]
** [1.21,1.55]
0.6
0.67
0.74
0.82
0.9
1
1.11
1.22
1.35
1.5
1.66


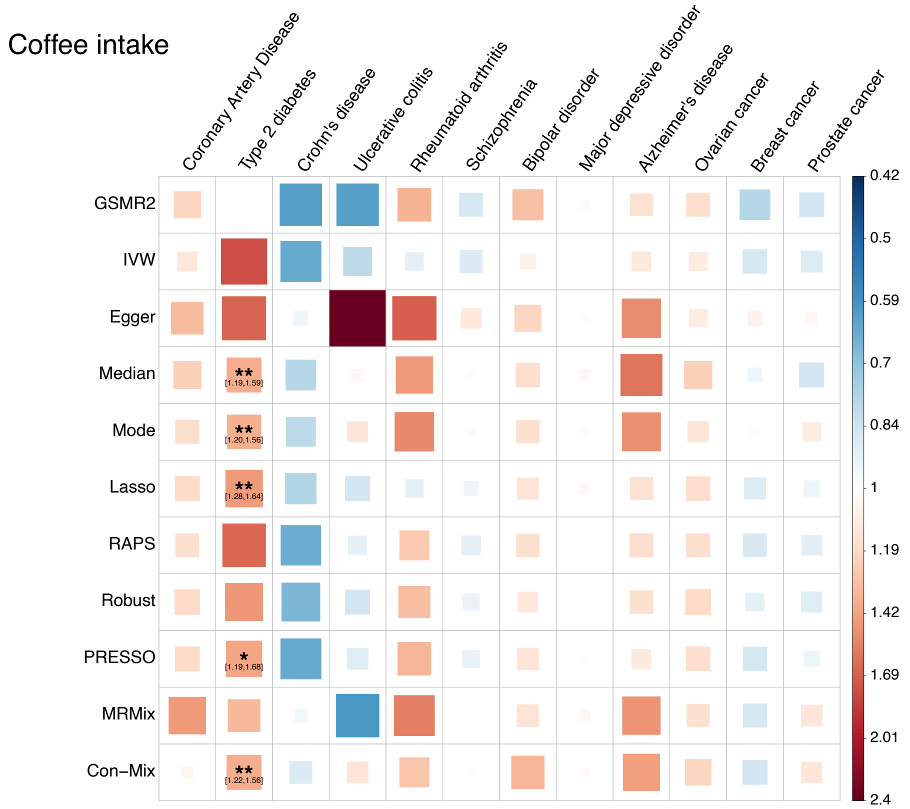

Coffee intake
Coronary Artery Disease
Type 2 diabetes
Crohn's disease
Ulcerative colitis
Rheumatoid arthritis
Schizophrenia
Bipolar disorder
Major depressive disorder
Alzheimer's disease
Ovarian cancer
Breast cancer
Prostate cancer
GSMR2
IVW
Egger
Median
Mode
Lasso
RAPS
Robust
PRESSO
MRMix
Con−Mix
** [1.19,1.59]
** [1.20,1.56]
** [1.28,1.64]
* [1.19,1.68]
** [1.22,1.56]
0.42
0.5
0.59
0.7
0.84
1
1.19
1.42
1.69
2.01
2.4

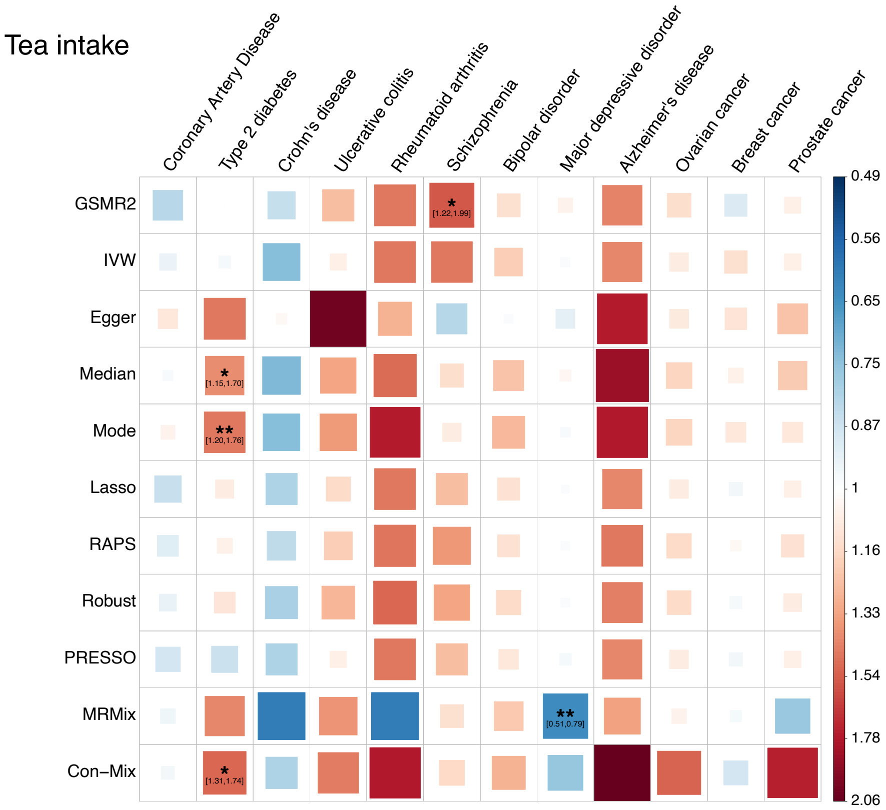


**Supplementary Figure 17. Estimates of causal effects of 7 substance use behaviours on 12 common diseases.** The analyses were performed using SUB GWAS summary data generated in this study using the UKB data and disease GWAS summary data from published studies. The exposure name is annotated on the top left of each panel. The size and opacity of the square is proportional to the size of the causal effect estimate. The suggestive estimates (local FDR < 0.05) are annotated with "*" and "[95% confidence interval]". The significant estimates with local FDR < 0.01 are labelled with "**". The names of the MR methods are shown on the left of the plot, and the names of the diseases are shown on the top.

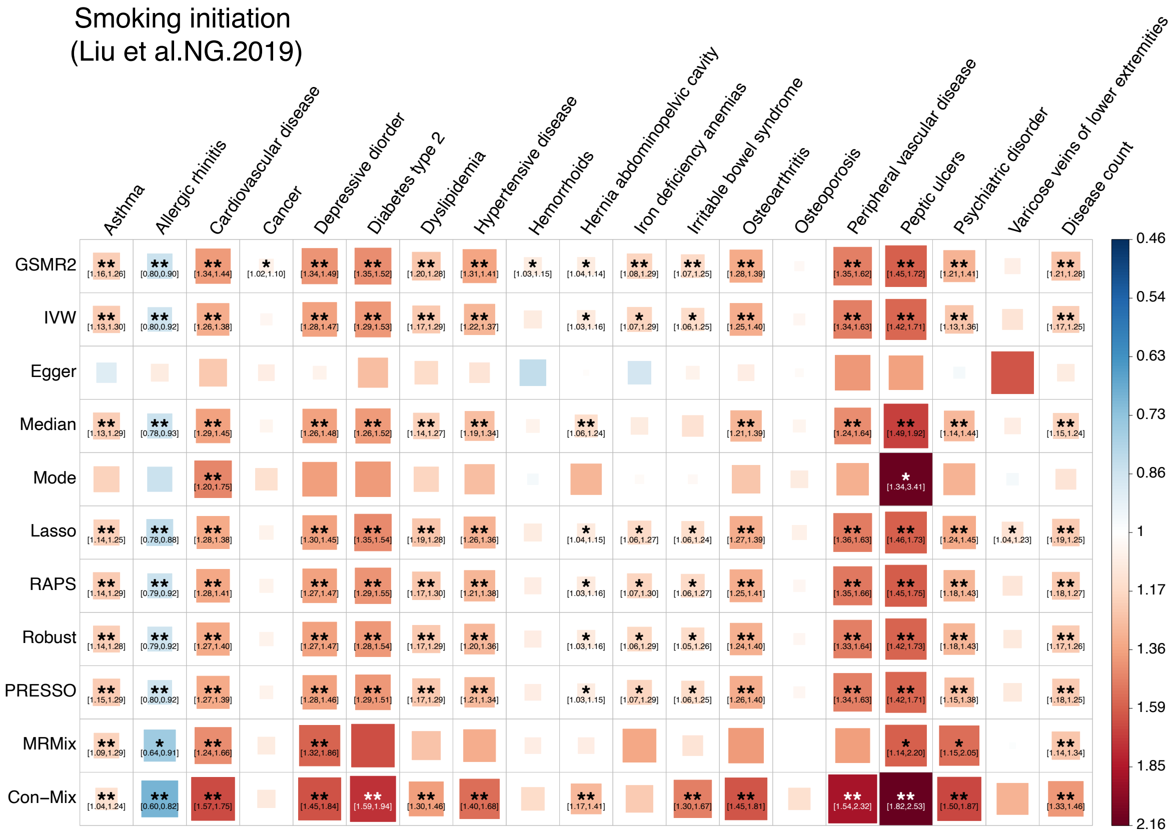
Smoking initiation
(Liu et al.NG.2019)
Asthma
Allergic rhinitis
Cardiovascular disease
Cancer
Depressive diorder
Diabetes type 2
Dyslipidemia
Hypertensive disease
Hemorrhoids
Hernia abdominopelvic cavity
Iron deficiency anemias
Irritable bowel syndrome
Osteoarthritis
Osteoporosis
Peripheral vascular disease
Peptic ulcers
Psychiatric disorder
Varicose veins of lower extremities
Disease count
GSMR2
IVW
Egger
Median
Mode
Lasso
RAPS
Robust
PRESSO
MRMix
Con−Mix
0.46
0.54
0.63
0.73
0.86
1
1.17
1.36
1.59
1.85
2.16

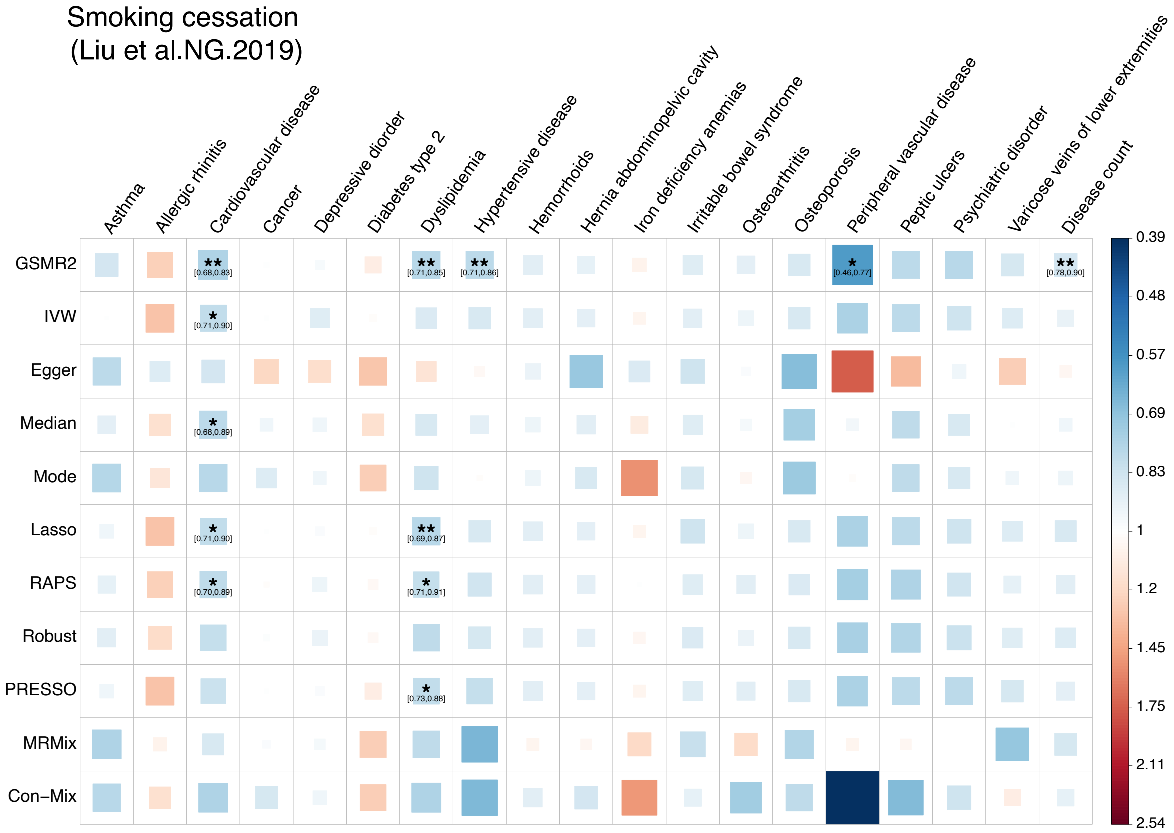
Smoking cessation
(Liu et al.NG.2019)
Asthma
Allergic rhinitis
Cardiovascular disease
Cancer
Depressive diorder
Diabetes type 2
Dyslipidemia
Hypertensive disease
Hemorrhoids
Hernia abdominopelvic cavity
Iron deficiency anemias
Irritable bowel syndrome
Osteoarthritis
Osteoporosis
Peripheral vascular disease
Peptic ulcers
Psychiatric disorder
Varicose veins of lower extremities
Disease count
GSMR2
IVW
Egger
Median
Mode
Lasso
RAPS
Robust
PRESSO
MRMix
Con−Mix
0.39
0.48
0.57
0.69
0.83
1
1.2
1.45
1.75
2.11
2.54

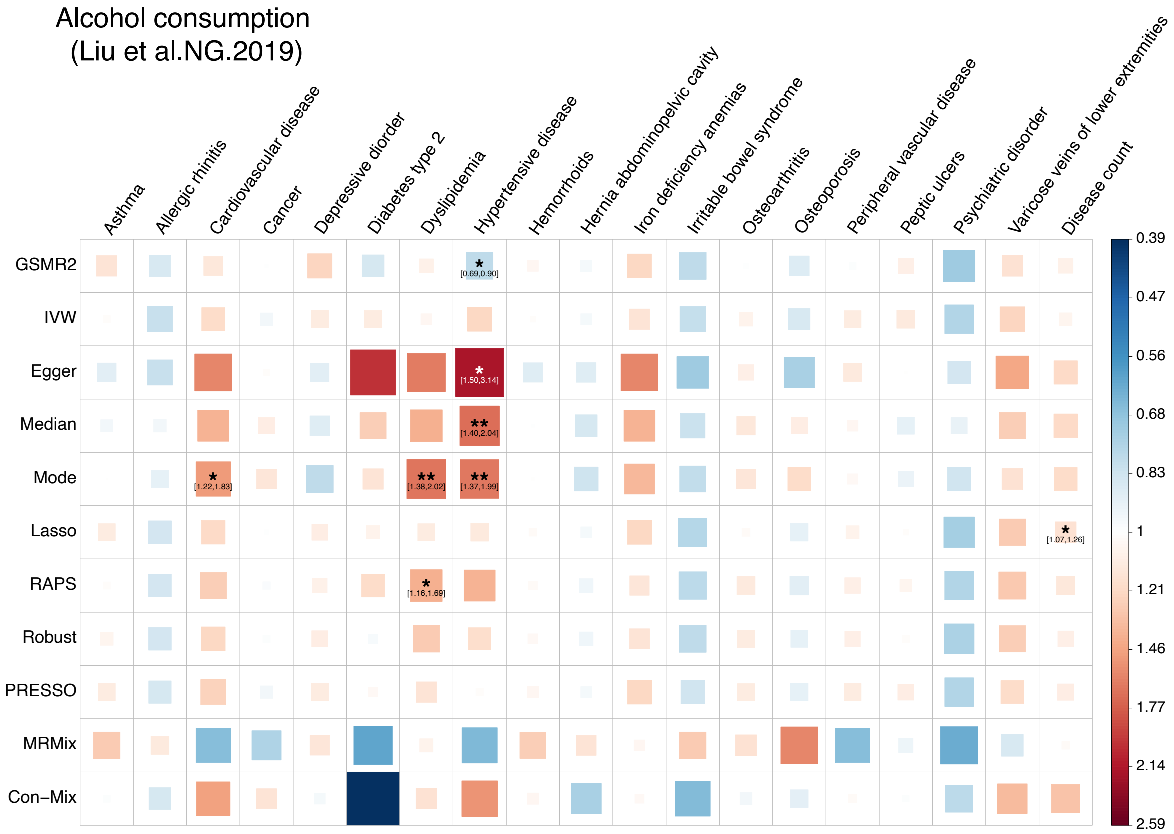


**Supplementary Figure 18. Estimates of the causal effects of substance use behaviours from GSCAN on common diseases from UKB.** Data for exposure were from GSCAN consortium and outcomes were from the UK Biobank. The exposure name is annotated on the top left of each panel. For each of the three exposures, 317, 18, and 84 index SNPs were used as instrumental variables, respectively. Each square represents an MR association result, and the colour indicates the direction of the estimate. The size and opacity of the square is proportional to the size of the causal effect estimate. The suggestive estimates (local FDR < 0.05) are annotated with "*" and "[95% confidence interval]". The significant estimates with local FDR < 0.01 are labelled with "**". The names of the MR methods are shown on the left of the plot, and the names of the diseases are shown on the top.

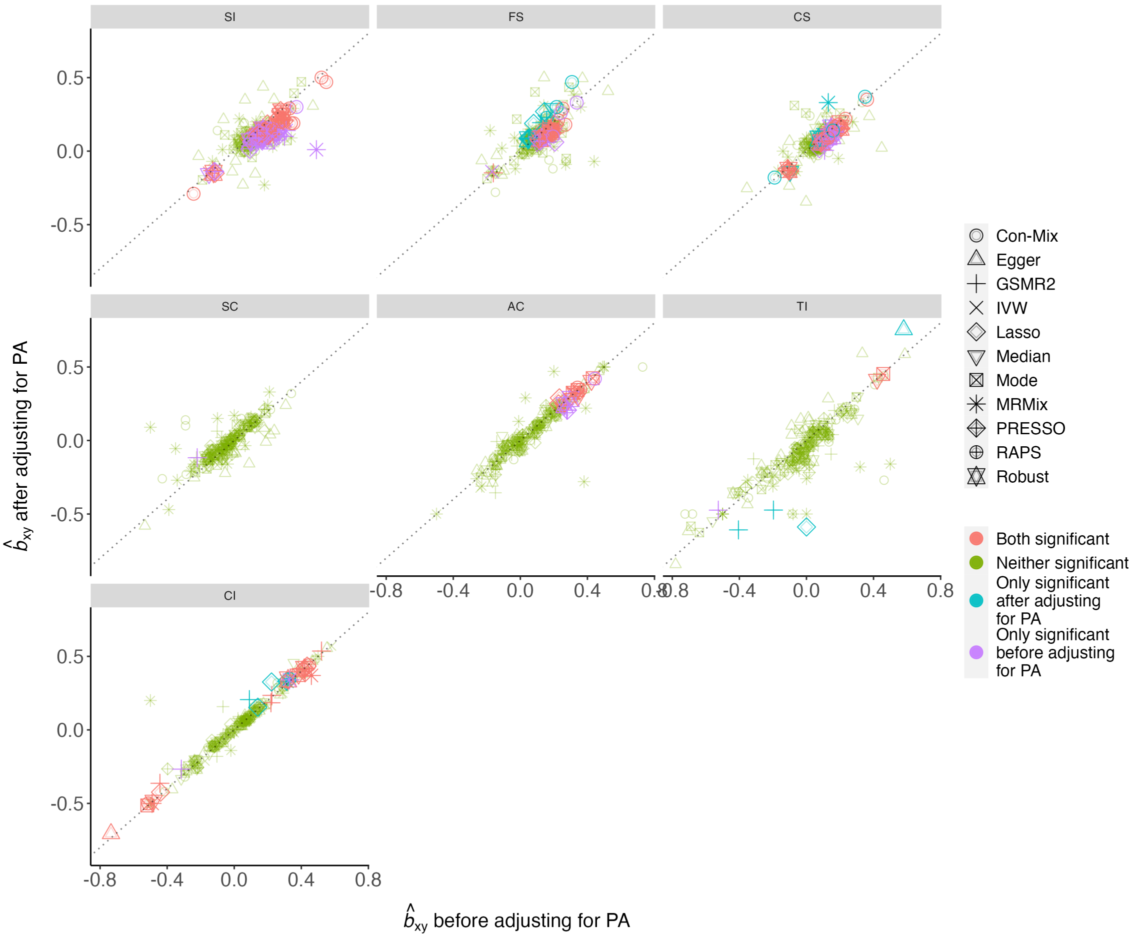


**Supplementary Figure 19. Comparison of estimates of causal effects of substance use behaviours on common diseases before and after adjusting for physical activity**. This figure shows the comparison of $b_{xy}$ estimates before and after adjusting for PA traits. Each panel indicates the exposure used. The color of each dot indicates the significance level change before and after PA adjustment. The significant level is defined at local FDR < 0.01. The shape of each dot indicates the MR method used. The grey dashed line is the diagonal line of the coordinate plane.

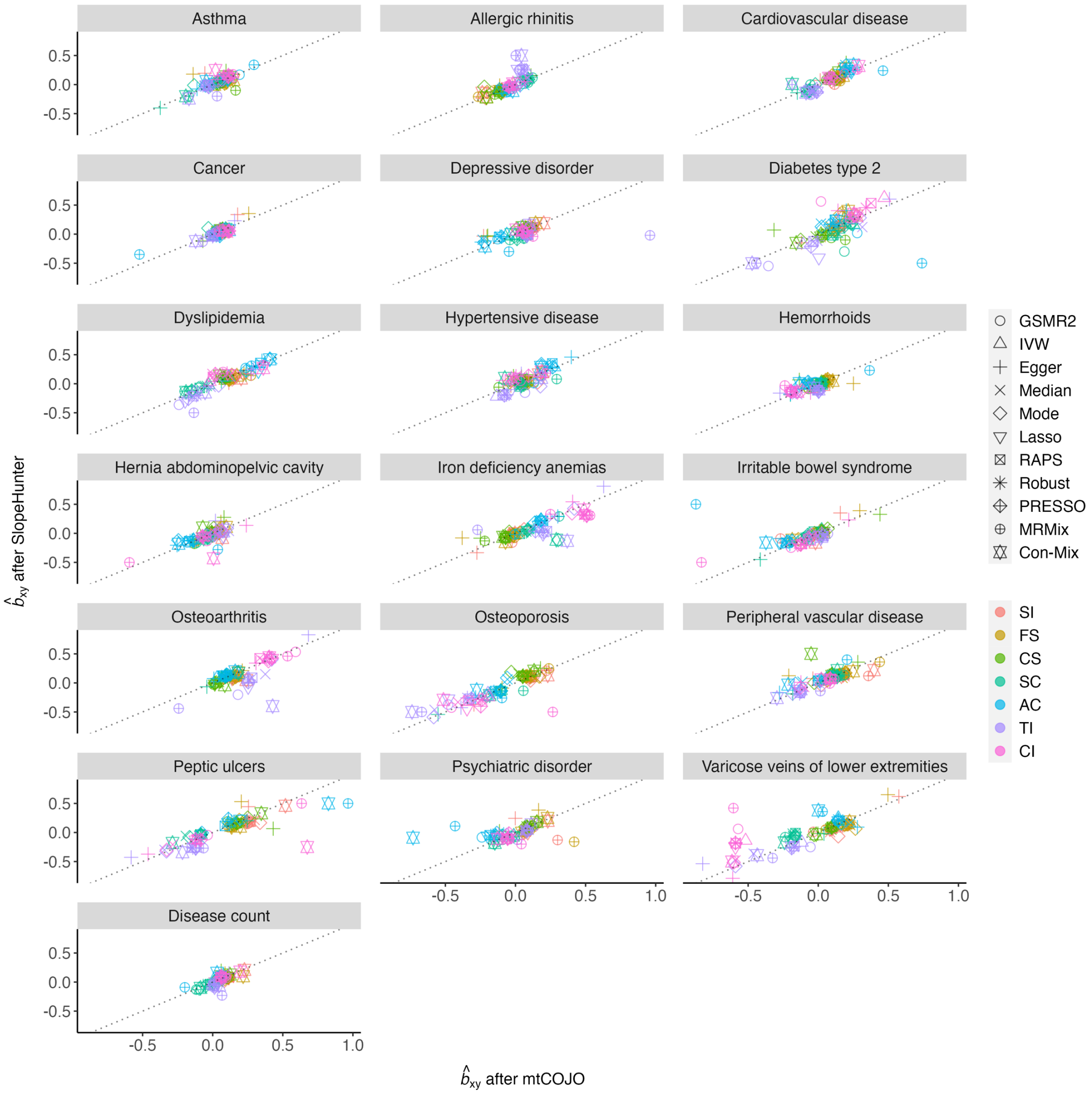


**Supplementary Figure 20. Comparison of MR results using GWAS summary data from mtCOJO and SlopeHunter.** This figure shows the comparison of $b_{xy}$ estimates after adjusting for SES using mtCOJO and SlopeHunter. Each panel indicates the outcome used. The color of each dot indicates the exposure used, and the shape of each dot indicates the MR method used. The grey dashed line represents the diagonal line of the coordinate plane.

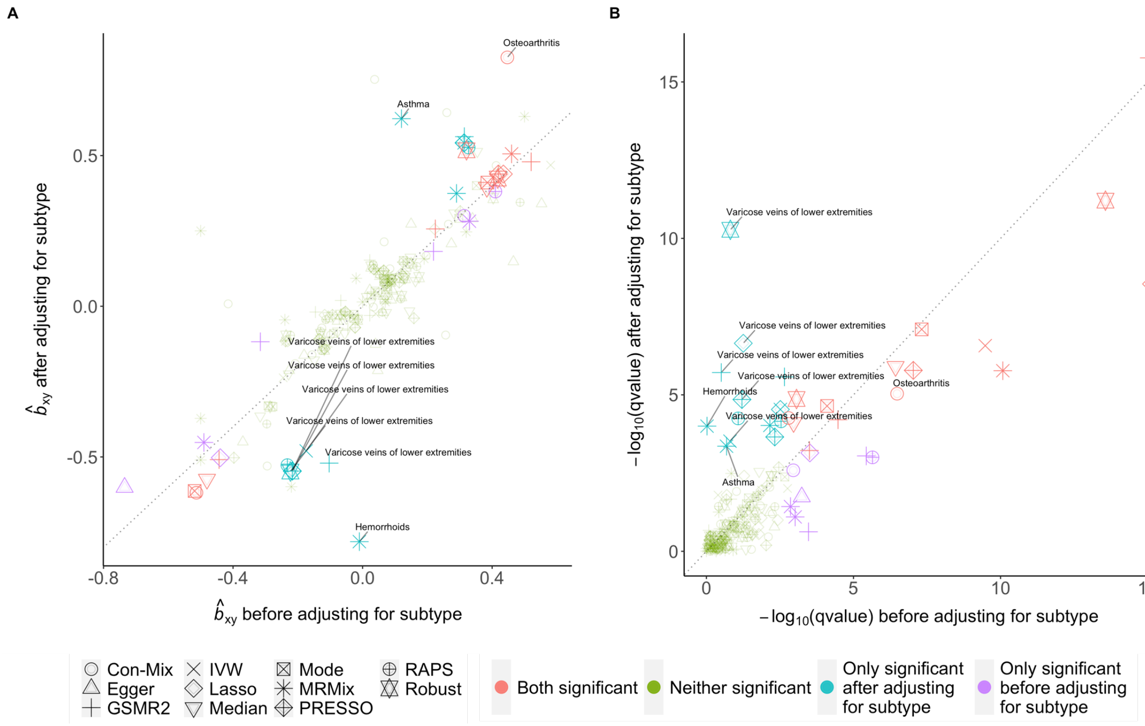


**Supplementary Figure 21. Comparison of the estimates of causal effects of coffee intake on common diseases before and after adjusting for coffee subtypes.** In panel A, the x-axis indicates the $b_{xy}$ estimate from MR analysis using coffee intake GWAS data without adjustment for coffee subtypes, while the y-axis indicates the $b_{xy}$ estimate after adjusting for coffee subtypes. In panel B, x-axis denotes $-\log_{10}$(qvalue) from MR analysis using coffee intake without adjustment, while y-axis denotes the $-\log_{10}$(qvalue) after adjusting for coffee subtypes. The colours of the dots represent the significance levels of the MR estimates. Those associations with large difference in MR estimate or significance level are annotated with "outcome".

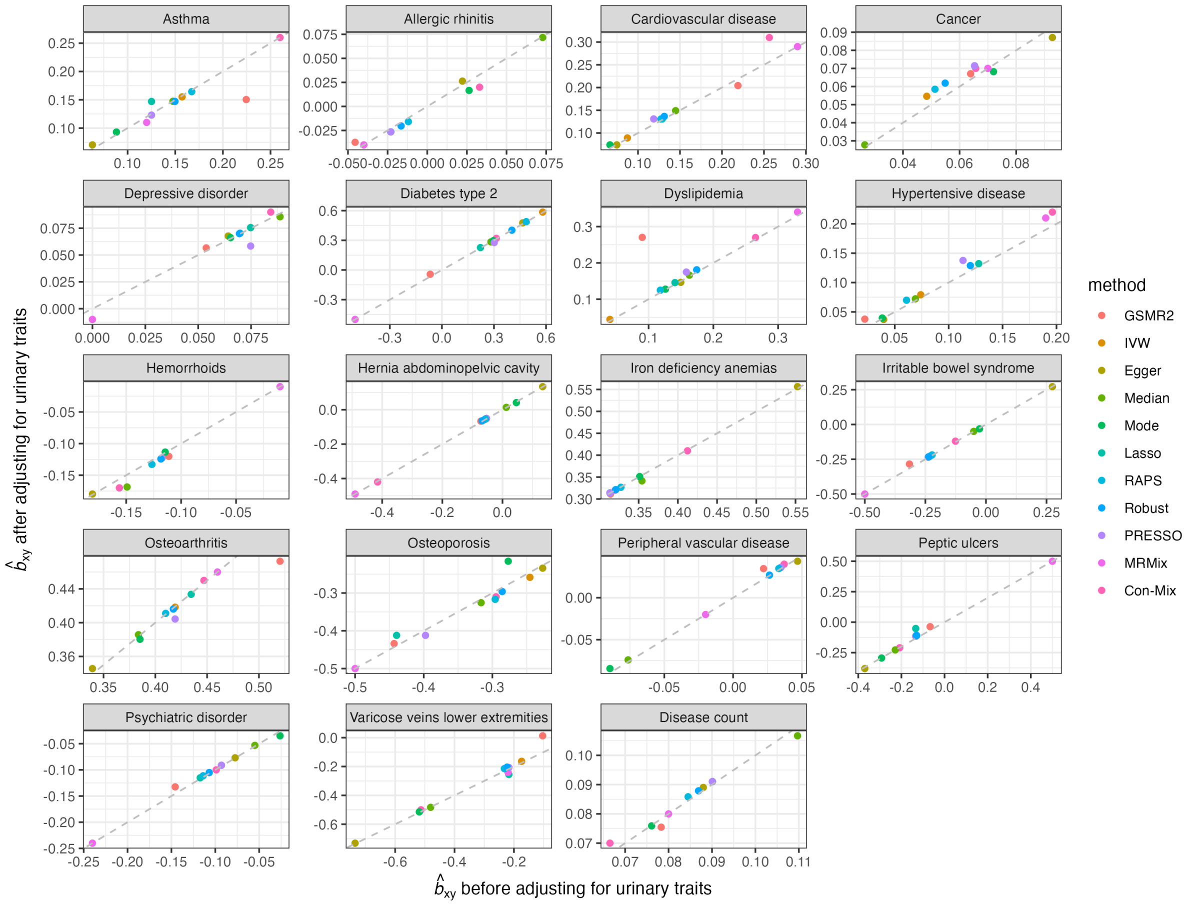


**Supplementary Figure 22. Comparison of estimates of causal effects of coffee intake on common diseases before and after adjusting for urinary traits.** This figure shows the comparison of $b_{xy}$ estimates after adjusting for urinary traits using mtCOJO. Each panel indicates the outcome used. The color of each dot indicates the MR method used. The grey dashed line represents the diagonal line of the coordinate plane.

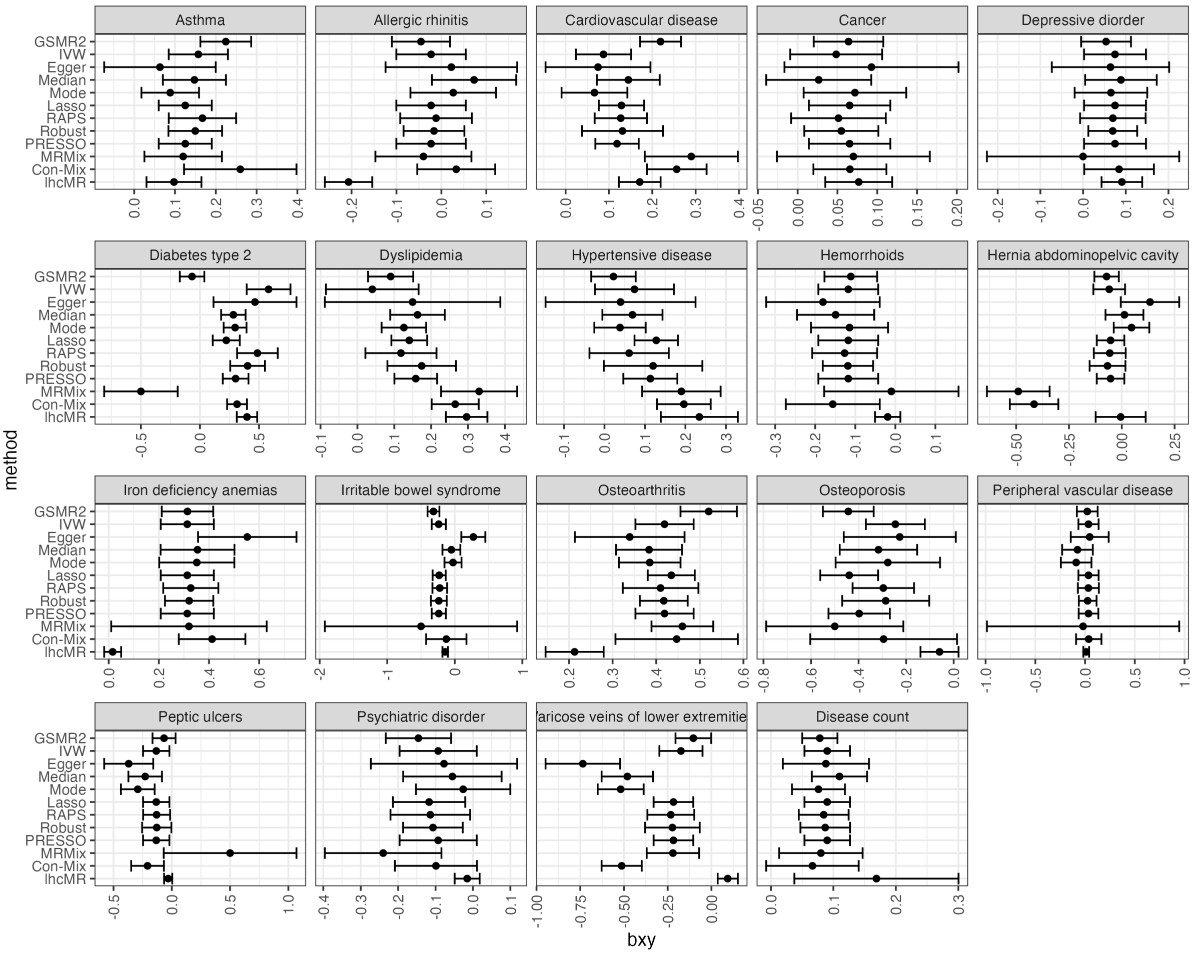


**Supplementary Figure 23. Comparison of estimates of causal effects of coffee intake on common diseases across different MR methods with LHC-MR.** This figure shows the comparison of $b_{xy}$ estimates of coffee intake on common diseases across 11 MR methods with lhcMR. Each panel indicates the disease outcome. The error bar indicates the standard error of the estimates. LHC-MR identified a significant negative estimate of CI on allergic rhinitis ($b_{xy}$ = -0.2067, se = 0.0527, $p$-value = 8.88E-05), but all other methods showed no significant effect. LHC-MR identified a reverse causal effect ($b_{yx}$ = 0.2502, se = 0.0944, $p$-value = 8.08E-03) (**Supplementary Table 15**), which might explain why other methods yielded insignificant results.

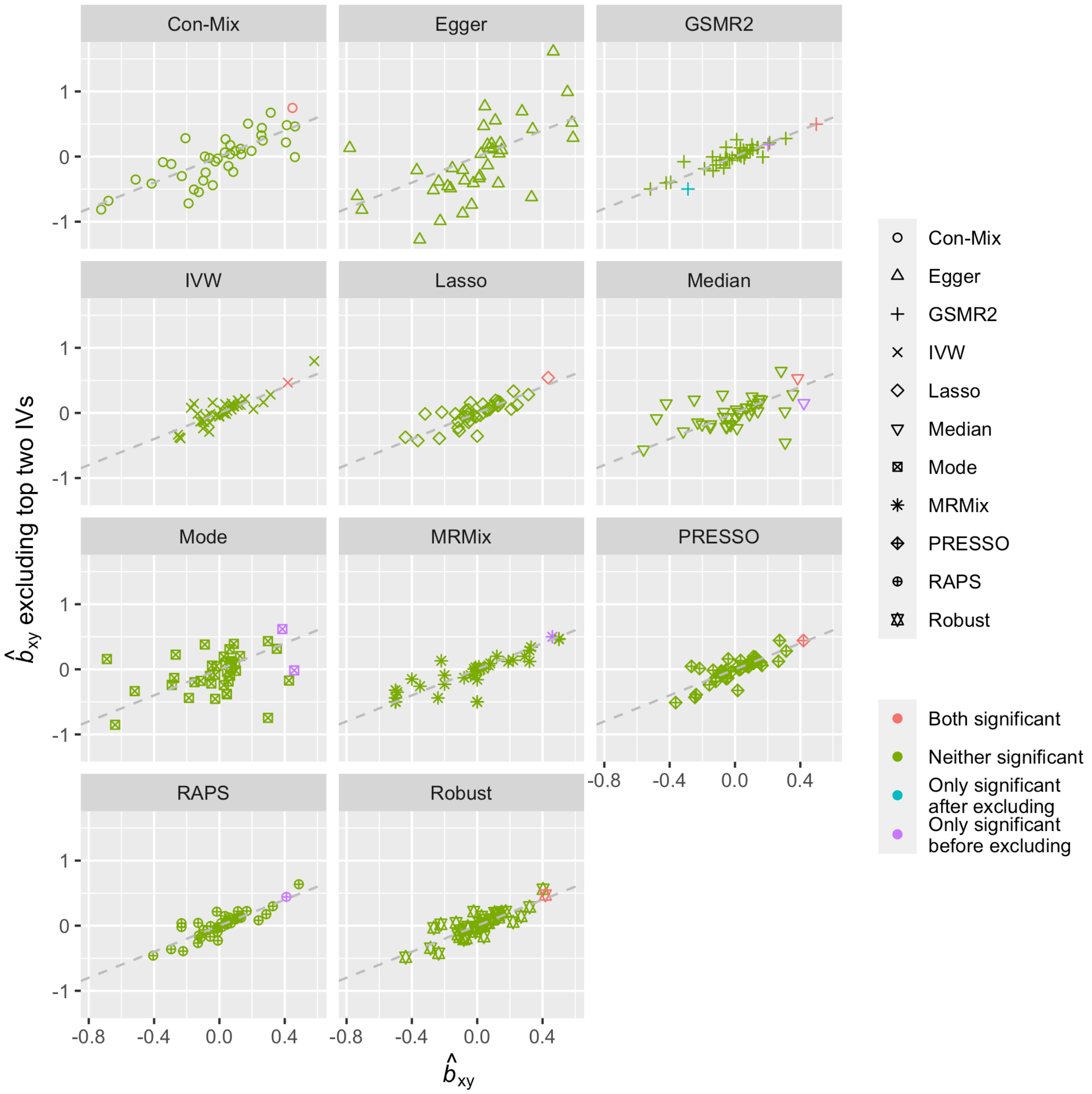


**Supplementary Figure 24. Comparison of the estimates of causal effects of coffee intake on common diseases before and after excluding the top two IVs.** This figure shows the comparison of $b_{xy}$ estimates before and after excluding the top two IVs for coffee intake GWAS. Each panel indicates the MR method used for estimation. The color of each dot indicates the significance level change before and after excluding the top two IVs. Significant level is defined at local FDR < 0.01. The shape of each dot indicates the MR method used. The grey dashed line is the diagonal line of the coordinate plane.
