# Supplementary material for: Unravelling the complex causal effects of substance use behaviours on common diseases
Source: Commun Med (Lond). 2024 Mar 12;4:43. doi: 10.1038/s43856-024-00473-3 (PMC10933313; doi:10.1038/s43856-024-00473-3)
Supplement: Supplementary file 3 — Description of Additional Supplementary Files [file 43856_2024_473_MOESM3_ESM.pdf]

Description of Additional Supplementary Files

File Name: Supplementary Data 1
Description: Causal effect of substance use behaviours on common diseases estimated at individual IVs.

File Name: Supplementary Data 2
Description: Causal estimates between smoking initiation and common diseases in UKBiobank.

File Name: Supplementary Data 3
Description: Causal estimates between former smoking and common diseases in UKBiobank.

File Name: Supplementary Data 4
Description: Causal estimates between current smoking and common diseases in UKBiobank.

File Name: Supplementary Data 5
Description: Causal estimates between smoking cessation and common diseases in UKBiobank

File Name: Supplementary Data 6
Description: Causal estimates between alcohol consumption and common diseases in UKBiobank

File Name: Supplementary Data 7
Description: Causal estimates between moderate and heavy alcohol consumption and common diseases in UKBiobank

File Name: Supplementary Data 8
Description: Causal estimates between coffee intake and common diseases in UKBiobank

File Name: Supplementary Data 9
Description: Causal estimates between tea intake and common diseases in UKBiobank

File Name: Supplementary Data 10
Description: Estimates of genetic correlation between coffee/tea intake and common diseases

File Name: Supplementary Data 11

Description: Causal estimates between substance use behaviours and common diseases adjuted socio-economic status in UKBiobank

File Name: Supplementary Data 12
Description: Causal estimates between common diseases and SUB

File Name: Supplementary Data 13
Description: Bi-directional causal estimates of coffee intake on common diseases using lhcMR
