# Supplementary material for: Unravelling the complex causal effects of substance use behaviours on common diseases
Source: Commun Med (Lond). 2024 Mar 12;4:43. doi: 10.1038/s43856-024-00473-3 (PMC10933313; doi:10.1038/s43856-024-00473-3)
Supplement: Supplementary file 4 — Supplementary Data 1 [file 43856_2024_473_MOESM4_ESM.pdf]

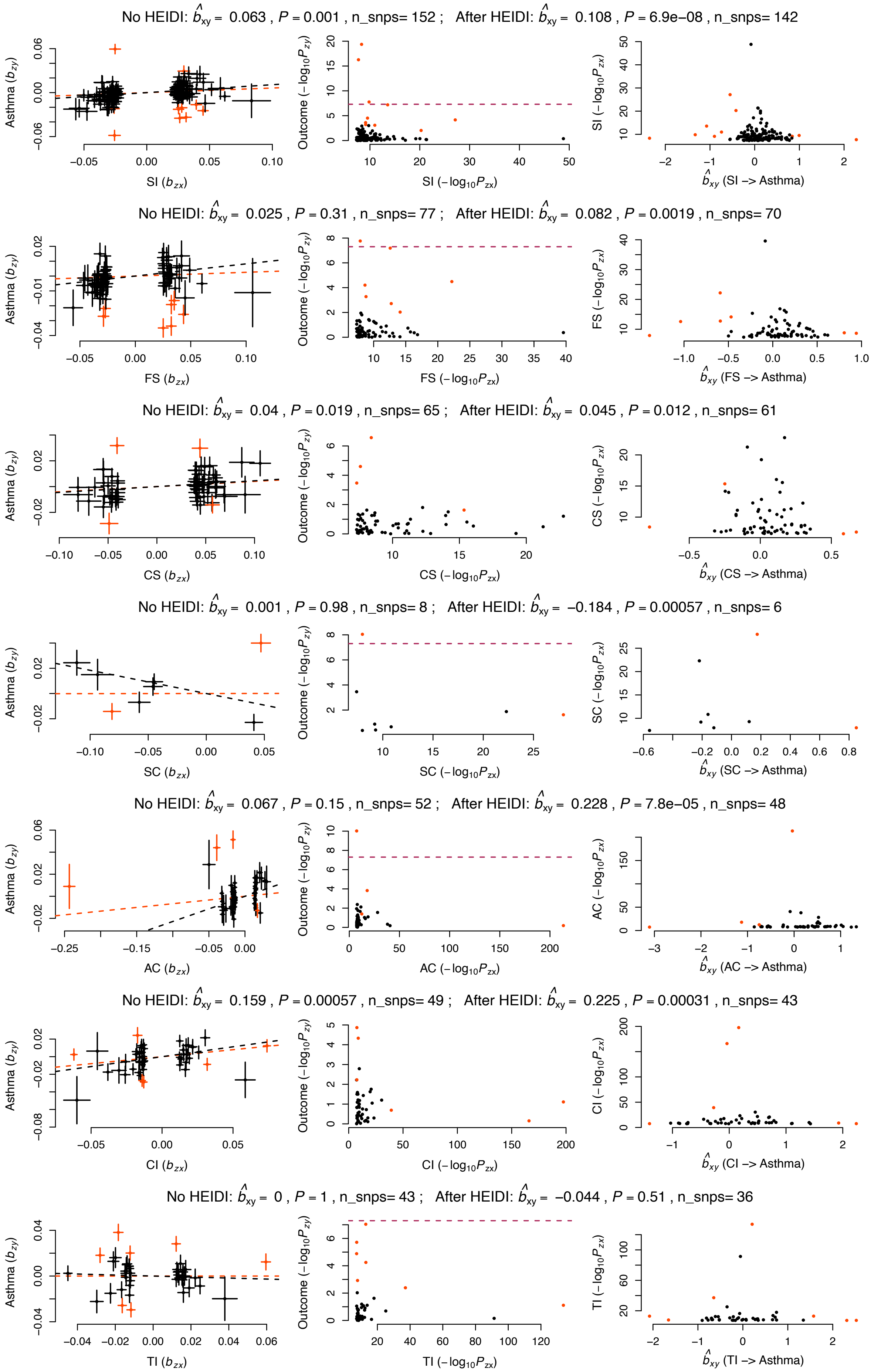

No HEIDI: $\hat{b}_{xy}$ = 0.063 , P = 0.001 , n_snps= 152 ; After HEIDI: $\hat{b}_{xy}$ = 0.108 , P = 6.9e−08 , n_snps= 142
Asthma (b_{zy})
SI (b_{zx})
Outcome ($-\log_{10}P_{zy}$)
SI ($-\log_{10}P_{zx}$)
SI ($-\log_{10}P_{zx}$)
$\hat{b}_{xy}$ (SI −> Asthma)
No HEIDI: $\hat{b}_{xy}$ = 0.025 , P = 0.31 , n_snps= 77 ; After HEIDI: $\hat{b}_{xy}$ = 0.082 , P = 0.0019 , n_snps= 70
Asthma (b_{zy})
FS (b_{zx})
Outcome ($-\log_{10}P_{zy}$)
FS ($-\log_{10}P_{zx}$)
FS ($-\log_{10}P_{zx}$)
$\hat{b}_{xy}$ (FS −> Asthma)
No HEIDI: $\hat{b}_{xy}$ = 0.04 , P = 0.019 , n_snps= 65 ; After HEIDI: $\hat{b}_{xy}$ = 0.045 , P = 0.012 , n_snps= 61
Asthma (b_{zy})
CS (b_{zx})
Outcome ($-\log_{10}P_{zy}$)
CS ($-\log_{10}P_{zx}$)
CS ($-\log_{10}P_{zx}$)
$\hat{b}_{xy}$ (CS −> Asthma)
No HEIDI: $\hat{b}_{xy}$ = 0.001 , P = 0.98 , n_snps= 8 ; After HEIDI: $\hat{b}_{xy}$ = −0.184 , P = 0.00057 , n_snps= 6
Asthma (b_{zy})
SC (b_{zx})
Outcome ($-\log_{10}P_{zy}$)
SC ($-\log_{10}P_{zx}$)
SC ($-\log_{10}P_{zx}$)
$\hat{b}_{xy}$ (SC −> Asthma)
No HEIDI: $\hat{b}_{xy}$ = 0.067 , P = 0.15 , n_snps= 52 ; After HEIDI: $\hat{b}_{xy}$ = 0.228 , P = 7.8e−05 , n_snps= 48
Asthma (b_{zy})
AC (b_{zx})
Outcome ($-\log_{10}P_{zy}$)
AC ($-\log_{10}P_{zx}$)
AC ($-\log_{10}P_{zx}$)
$\hat{b}_{xy}$ (AC −> Asthma)
No HEIDI: $\hat{b}_{xy}$ = 0.159 , P = 0.00057 , n_snps= 49 ; After HEIDI: $\hat{b}_{xy}$ = 0.225 , P = 0.00031 , n_snps= 43
Asthma (b_{zy})
CI (b_{zx})
Outcome ($-\log_{10}P_{zy}$)
CI ($-\log_{10}P_{zx}$)
CI ($-\log_{10}P_{zx}$)
$\hat{b}_{xy}$ (CI −> Asthma)
No HEIDI: $\hat{b}_{xy}$ = 0 , P = 1 , n_snps= 43 ; After HEIDI: $\hat{b}_{xy}$ = −0.044 , P = 0.51 , n_snps= 36
Asthma (b_{zy})
TI (b_{zx})
Outcome ($-\log_{10}P_{zy}$)
TI ($-\log_{10}P_{zx}$)
TI ($-\log_{10}P_{zx}$)
$\hat{b}_{xy}$ (TI −> Asthma)

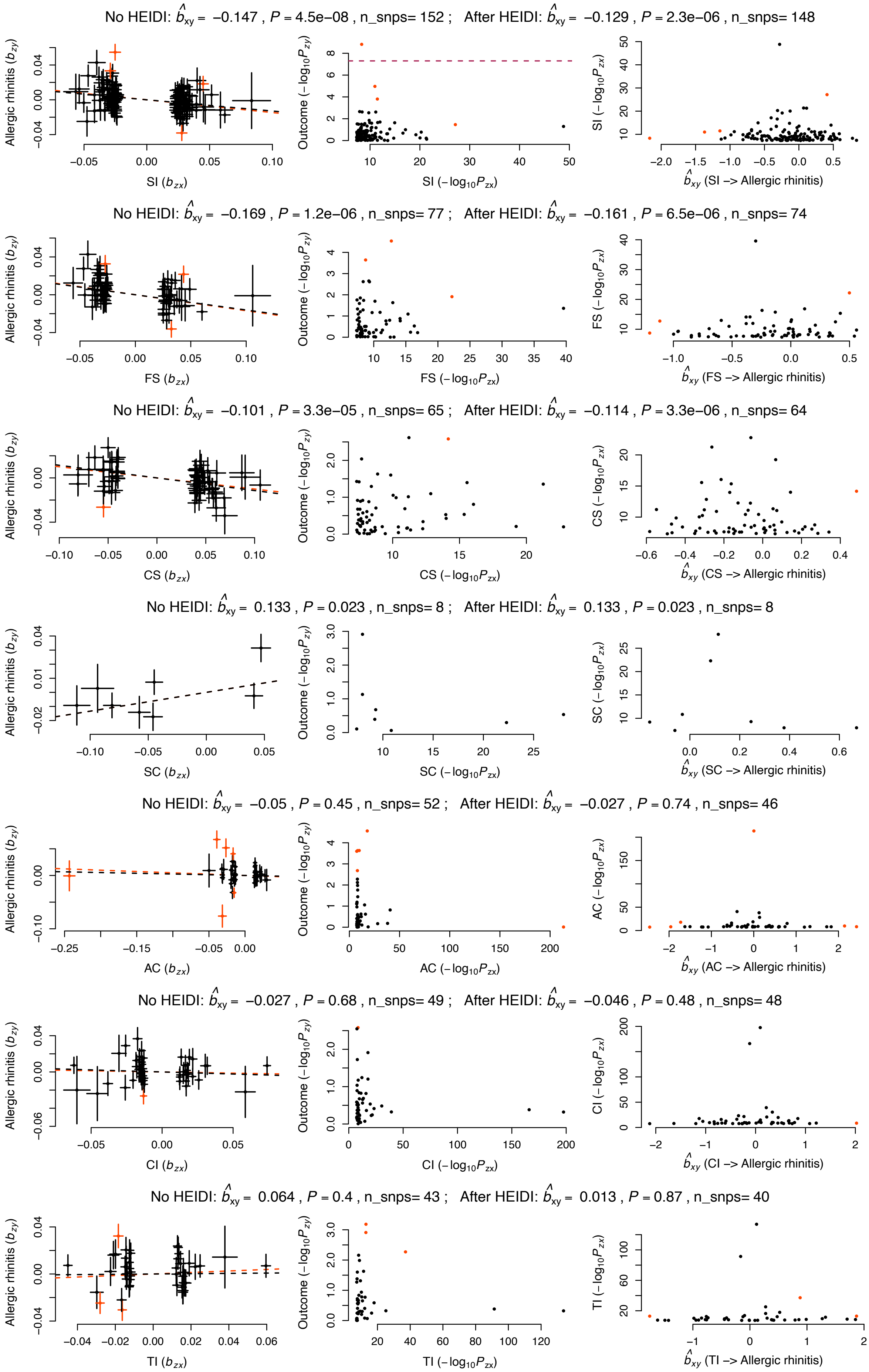
No HEIDI: $\hat{b}_{xy}$ = −0.147 , P = 4.5e−08 , n_snps= 152 ; After HEIDI: $\hat{b}_{xy}$ = −0.129 , P = 2.3e−06 , n_snps= 148
Allergic rhinitis (b_{zy})
SI (b_{zx})
Outcome ($-\log_{10}P_{zy}$)
SI ($-\log_{10}P_{zx}$)
$\hat{b}_{xy}$ (SI −> Allergic rhinitis)
No HEIDI: $\hat{b}_{xy}$ = −0.169 , P = 1.2e−06 , n_snps= 77 ; After HEIDI: $\hat{b}_{xy}$ = −0.161 , P = 6.5e−06 , n_snps= 74
FS (b_{zx})
FS ($-\log_{10}P_{zx}$)
$\hat{b}_{xy}$ (FS −> Allergic rhinitis)
No HEIDI: $\hat{b}_{xy}$ = −0.101 , P = 3.3e−05 , n_snps= 65 ; After HEIDI: $\hat{b}_{xy}$ = −0.114 , P = 3.3e−06 , n_snps= 64
CS (b_{zx})
CS ($-\log_{10}P_{zx}$)
$\hat{b}_{xy}$ (CS −> Allergic rhinitis)
No HEIDI: $\hat{b}_{xy}$ = 0.133 , P = 0.023 , n_snps= 8 ; After HEIDI: $\hat{b}_{xy}$ = 0.133 , P = 0.023 , n_snps= 8
SC (b_{zx})
SC ($-\log_{10}P_{zx}$)
$\hat{b}_{xy}$ (SC −> Allergic rhinitis)
No HEIDI: $\hat{b}_{xy}$ = −0.05 , P = 0.45 , n_snps= 52 ; After HEIDI: $\hat{b}_{xy}$ = −0.027 , P = 0.74 , n_snps= 46
AC (b_{zx})
AC ($-\log_{10}P_{zx}$)
$\hat{b}_{xy}$ (AC −> Allergic rhinitis)
No HEIDI: $\hat{b}_{xy}$ = −0.027 , P = 0.68 , n_snps= 49 ; After HEIDI: $\hat{b}_{xy}$ = −0.046 , P = 0.48 , n_snps= 48
CI (b_{zx})
CI ($-\log_{10}P_{zx}$)
$\hat{b}_{xy}$ (CI −> Allergic rhinitis)
No HEIDI: $\hat{b}_{xy}$ = 0.064 , P = 0.4 , n_snps= 43 ; After HEIDI: $\hat{b}_{xy}$ = 0.013 , P = 0.87 , n_snps= 40
TI (b_{zx})
TI ($-\log_{10}P_{zx}$)
$\hat{b}_{xy}$ (TI −> Allergic rhinitis)

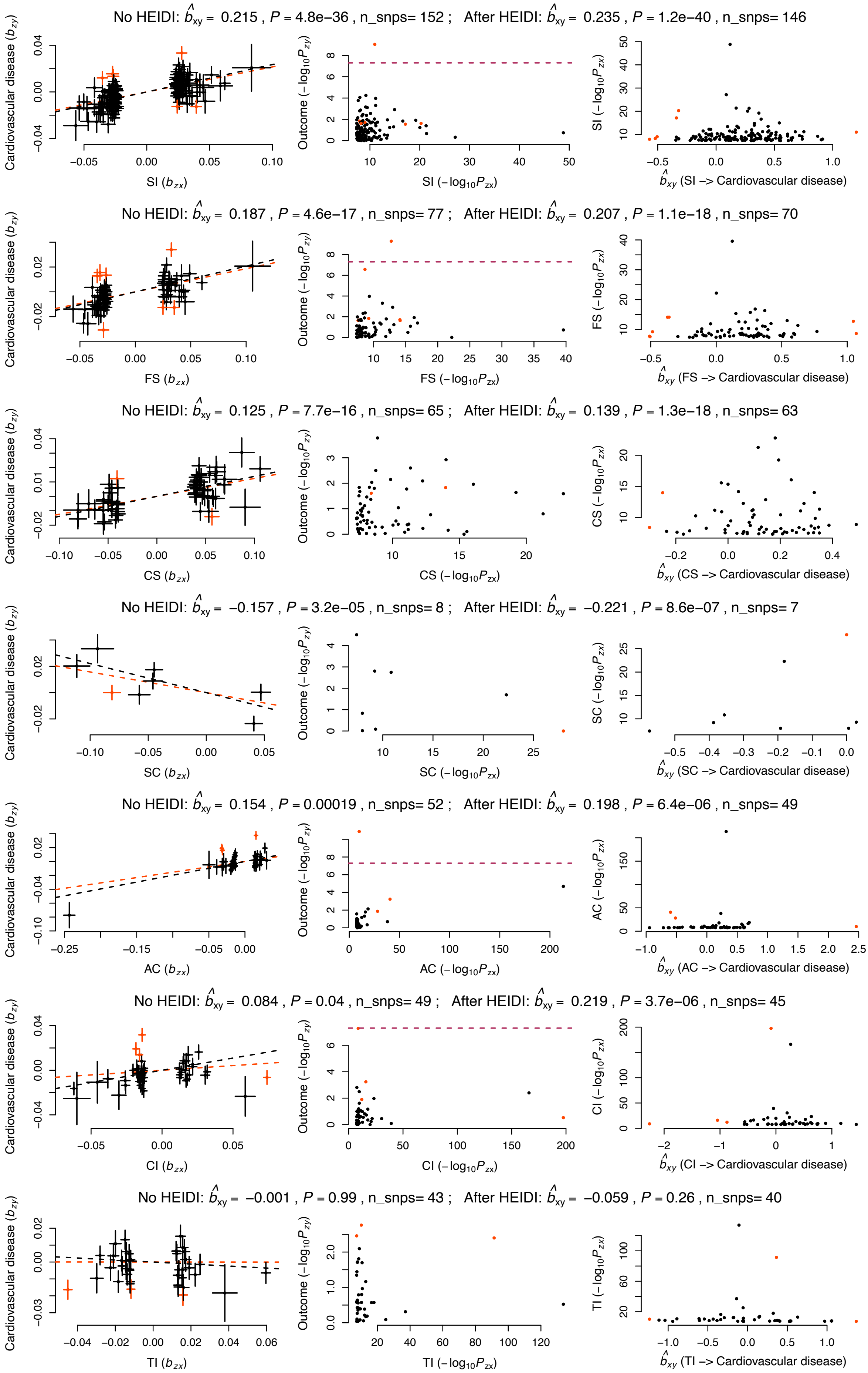
No HEIDI: $\hat{b}_{xy}$ = 0.215 , P = 4.8e−36 , n_snps= 152 ; After HEIDI: $\hat{b}_{xy}$ = 0.235 , P = 1.2e−40 , n_snps= 146
Cardiovascular disease (b_{zy})
SI (b_{zx})
Outcome ($-\log_{10}P_{zy}$)
SI ($-\log_{10}P_{zx}$)
$\hat{b}_{xy}$ (SI −> Cardiovascular disease)
No HEIDI: $\hat{b}_{xy}$ = 0.187 , P = 4.6e−17 , n_snps= 77 ; After HEIDI: $\hat{b}_{xy}$ = 0.207 , P = 1.1e−18 , n_snps= 70
Cardiovascular disease (b_{zy})
FS (b_{zx})
Outcome ($-\log_{10}P_{zy}$)
FS ($-\log_{10}P_{zx}$)
$\hat{b}_{xy}$ (FS −> Cardiovascular disease)
No HEIDI: $\hat{b}_{xy}$ = 0.125 , P = 7.7e−16 , n_snps= 65 ; After HEIDI: $\hat{b}_{xy}$ = 0.139 , P = 1.3e−18 , n_snps= 63
Cardiovascular disease (b_{zy})
CS (b_{zx})
Outcome ($-\log_{10}P_{zy}$)
CS ($-\log_{10}P_{zx}$)
$\hat{b}_{xy}$ (CS −> Cardiovascular disease)
No HEIDI: $\hat{b}_{xy}$ = −0.157 , P = 3.2e−05 , n_snps= 8 ; After HEIDI: $\hat{b}_{xy}$ = −0.221 , P = 8.6e−07 , n_snps= 7
Cardiovascular disease (b_{zy})
SC (b_{zx})
Outcome ($-\log_{10}P_{zy}$)
SC ($-\log_{10}P_{zx}$)
$\hat{b}_{xy}$ (SC −> Cardiovascular disease)
No HEIDI: $\hat{b}_{xy}$ = 0.154 , P = 0.00019 , n_snps= 52 ; After HEIDI: $\hat{b}_{xy}$ = 0.198 , P = 6.4e−06 , n_snps= 49
Cardiovascular disease (b_{zy})
AC (b_{zx})
Outcome ($-\log_{10}P_{zy}$)
AC ($-\log_{10}P_{zx}$)
$\hat{b}_{xy}$ (AC −> Cardiovascular disease)
No HEIDI: $\hat{b}_{xy}$ = 0.084 , P = 0.04 , n_snps= 49 ; After HEIDI: $\hat{b}_{xy}$ = 0.219 , P = 3.7e−06 , n_snps= 45
Cardiovascular disease (b_{zy})
CI (b_{zx})
Outcome ($-\log_{10}P_{zy}$)
CI ($-\log_{10}P_{zx}$)
$\hat{b}_{xy}$ (CI −> Cardiovascular disease)
No HEIDI: $\hat{b}_{xy}$ = −0.001 , P = 0.99 , n_snps= 43 ; After HEIDI: $\hat{b}_{xy}$ = −0.059 , P = 0.26 , n_snps= 40
Cardiovascular disease (b_{zy})
TI (b_{zx})
Outcome ($-\log_{10}P_{zy}$)
TI ($-\log_{10}P_{zx}$)
$\hat{b}_{xy}$ (TI −> Cardiovascular disease)

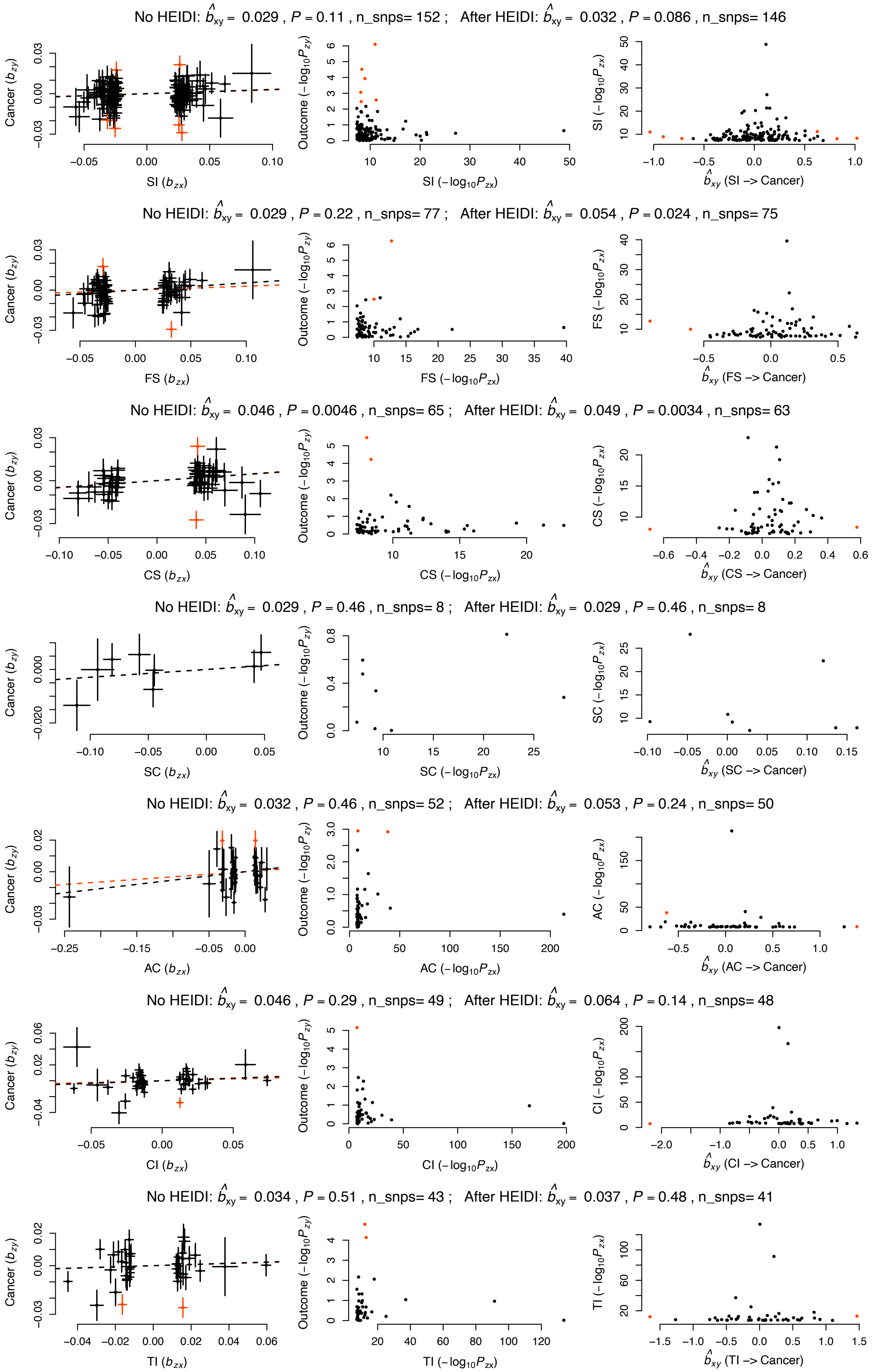

No HEIDI: $\hat{b}_{xy}$ = 0.029 , P = 0.11 , n_snps= 152 ; After HEIDI: $\hat{b}_{xy}$ = 0.032 , P = 0.086 , n_snps= 146
Cancer (b_{zy})
SI (b_{zx})
Outcome ($-\log_{10}P_{zy}$)
SI ($-\log_{10}P_{zx}$)
$\hat{b}_{xy}$ (SI -> Cancer)
No HEIDI: $\hat{b}_{xy}$ = 0.029 , P = 0.22 , n_snps= 77 ; After HEIDI: $\hat{b}_{xy}$ = 0.054 , P = 0.024 , n_snps= 75
Cancer (b_{zy})
FS (b_{zx})
Outcome ($-\log_{10}P_{zy}$)
FS ($-\log_{10}P_{zx}$)
$\hat{b}_{xy}$ (FS -> Cancer)
No HEIDI: $\hat{b}_{xy}$ = 0.046 , P = 0.0046 , n_snps= 65 ; After HEIDI: $\hat{b}_{xy}$ = 0.049 , P = 0.0034 , n_snps= 63
Cancer (b_{zy})
CS (b_{zx})
Outcome ($-\log_{10}P_{zy}$)
CS ($-\log_{10}P_{zx}$)
$\hat{b}_{xy}$ (CS -> Cancer)
No HEIDI: $\hat{b}_{xy}$ = 0.029 , P = 0.46 , n_snps= 8 ; After HEIDI: $\hat{b}_{xy}$ = 0.029 , P = 0.46 , n_snps= 8
Cancer (b_{zy})
SC (b_{zx})
Outcome ($-\log_{10}P_{zy}$)
SC ($-\log_{10}P_{zx}$)
$\hat{b}_{xy}$ (SC -> Cancer)
No HEIDI: $\hat{b}_{xy}$ = 0.032 , P = 0.46 , n_snps= 52 ; After HEIDI: $\hat{b}_{xy}$ = 0.053 , P = 0.24 , n_snps= 50
Cancer (b_{zy})
AC (b_{zx})
Outcome ($-\log_{10}P_{zy}$)
AC ($-\log_{10}P_{zx}$)
$\hat{b}_{xy}$ (AC -> Cancer)
No HEIDI: $\hat{b}_{xy}$ = 0.046 , P = 0.29 , n_snps= 49 ; After HEIDI: $\hat{b}_{xy}$ = 0.064 , P = 0.14 , n_snps= 48
Cancer (b_{zy})
CI (b_{zx})
Outcome ($-\log_{10}P_{zy}$)
CI ($-\log_{10}P_{zx}$)
$\hat{b}_{xy}$ (CI -> Cancer)
No HEIDI: $\hat{b}_{xy}$ = 0.034 , P = 0.51 , n_snps= 43 ; After HEIDI: $\hat{b}_{xy}$ = 0.037 , P = 0.48 , n_snps= 41
Cancer (b_{zy})
TI (b_{zx})
Outcome ($-\log_{10}P_{zy}$)
TI ($-\log_{10}P_{zx}$)
$\hat{b}_{xy}$ (TI -> Cancer)

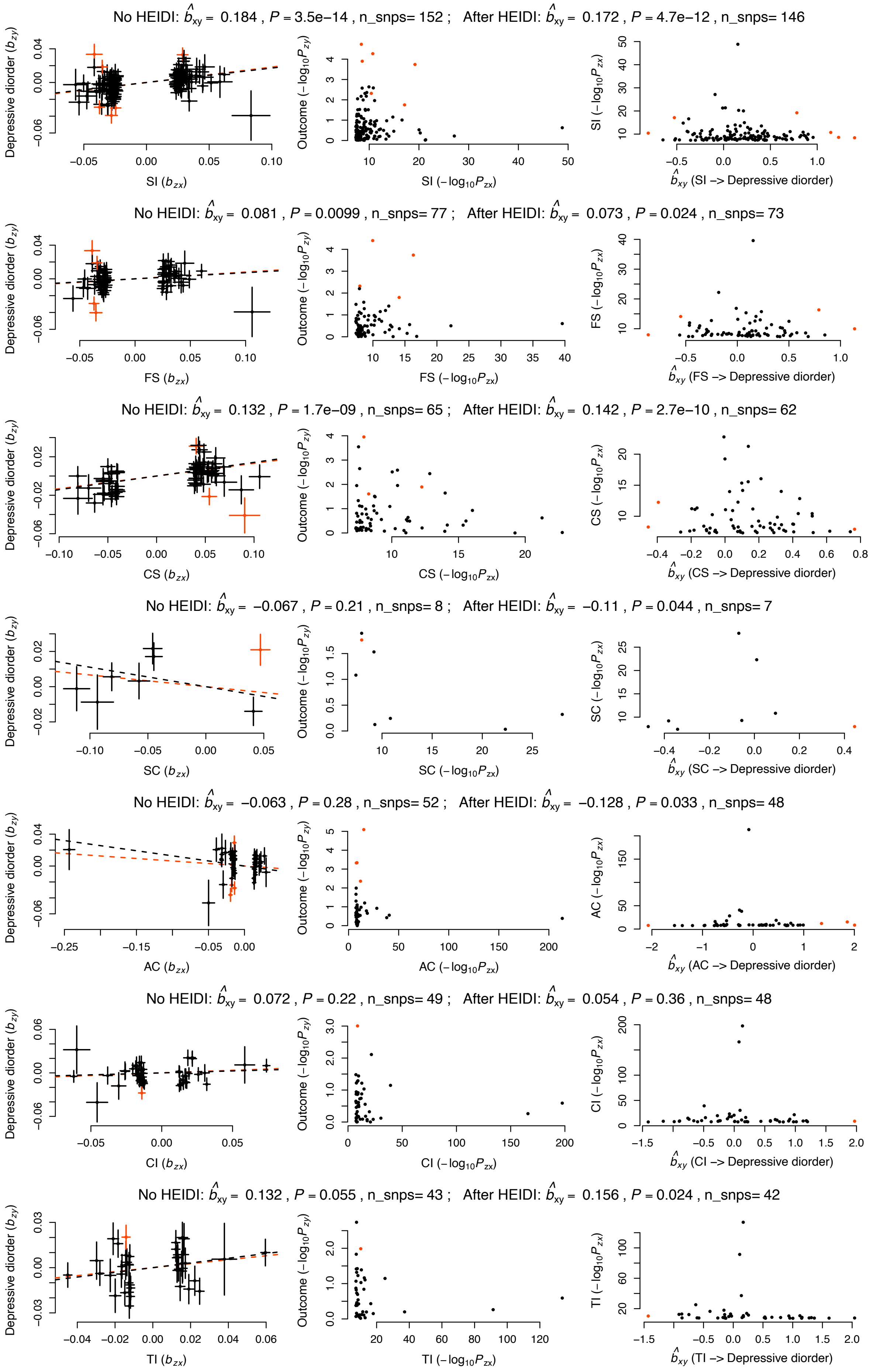
No HEIDI: $\hat{b}_{xy}$ = 0.184 , P = 3.5e−14 , n_snps= 152 ; After HEIDI: $\hat{b}_{xy}$ = 0.172 , P = 4.7e−12 , n_snps= 146
Depressive diorder (b_{zy})
SI (b_{zx})
Outcome ($-\log_{10}P_{zy}$)
SI ($-\log_{10}P_{zx}$)
$\hat{b}_{xy}$ (SI –> Depressive diorder)
No HEIDI: $\hat{b}_{xy}$ = 0.081 , P = 0.0099 , n_snps= 77 ; After HEIDI: $\hat{b}_{xy}$ = 0.073 , P = 0.024 , n_snps= 73
FS (b_{zx})
FS ($-\log_{10}P_{zx}$)
$\hat{b}_{xy}$ (FS –> Depressive diorder)
No HEIDI: $\hat{b}_{xy}$ = 0.132 , P = 1.7e−09 , n_snps= 65 ; After HEIDI: $\hat{b}_{xy}$ = 0.142 , P = 2.7e−10 , n_snps= 62
CS (b_{zx})
CS ($-\log_{10}P_{zx}$)
$\hat{b}_{xy}$ (CS –> Depressive diorder)
No HEIDI: $\hat{b}_{xy}$ = −0.067 , P = 0.21 , n_snps= 8 ; After HEIDI: $\hat{b}_{xy}$ = −0.11 , P = 0.044 , n_snps= 7
SC (b_{zx})
SC ($-\log_{10}P_{zx}$)
$\hat{b}_{xy}$ (SC –> Depressive diorder)
No HEIDI: $\hat{b}_{xy}$ = −0.063 , P = 0.28 , n_snps= 52 ; After HEIDI: $\hat{b}_{xy}$ = −0.128 , P = 0.033 , n_snps= 48
AC (b_{zx})
AC ($-\log_{10}P_{zx}$)
$\hat{b}_{xy}$ (AC –> Depressive diorder)
No HEIDI: $\hat{b}_{xy}$ = 0.072 , P = 0.22 , n_snps= 49 ; After HEIDI: $\hat{b}_{xy}$ = 0.054 , P = 0.36 , n_snps= 48
CI (b_{zx})
CI ($-\log_{10}P_{zx}$)
$\hat{b}_{xy}$ (CI –> Depressive diorder)
No HEIDI: $\hat{b}_{xy}$ = 0.132 , P = 0.055 , n_snps= 43 ; After HEIDI: $\hat{b}_{xy}$ = 0.156 , P = 0.024 , n_snps= 42
TI (b_{zx})
TI ($-\log_{10}P_{zx}$)
$\hat{b}_{xy}$ (TI –> Depressive diorder)

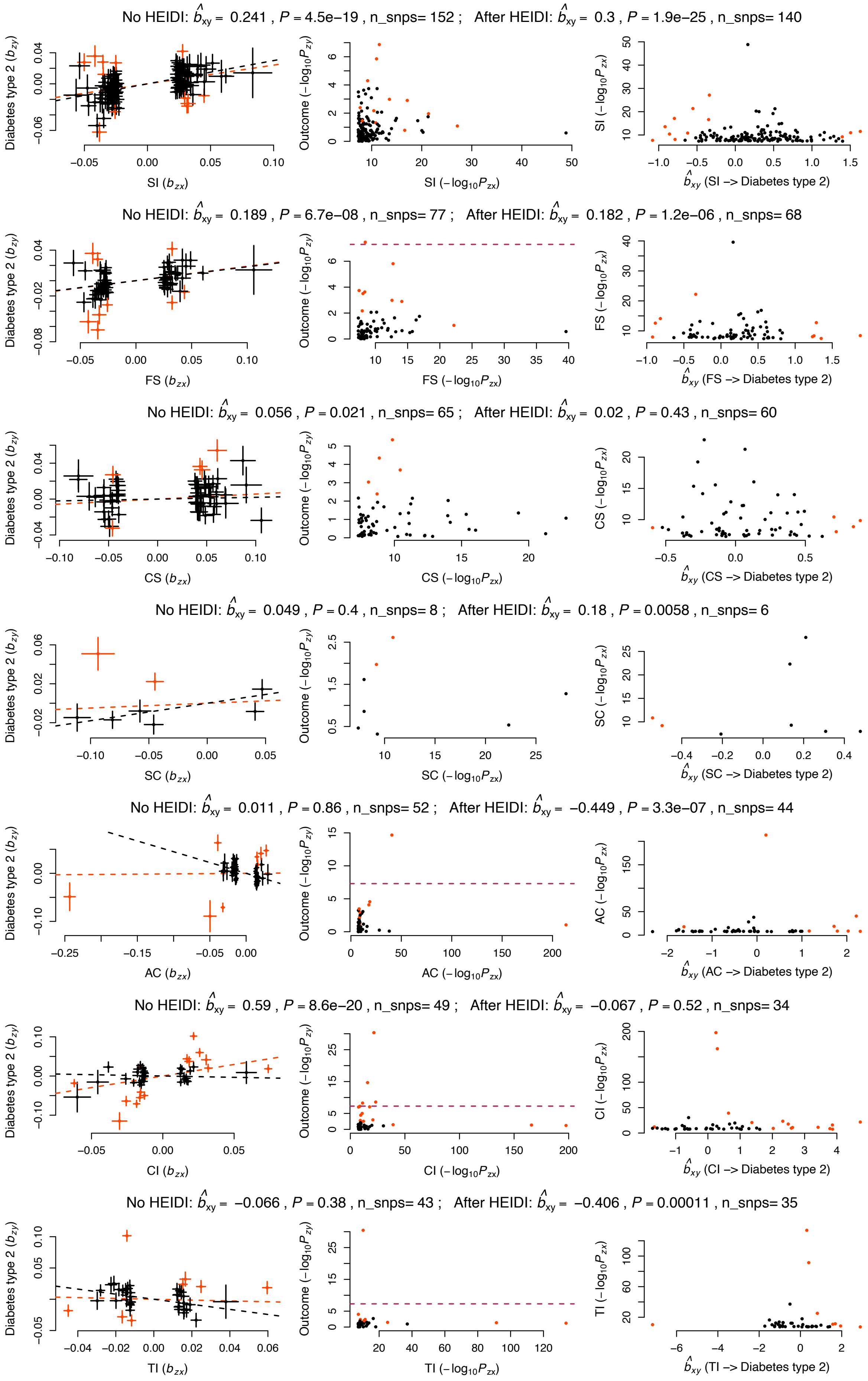
No HEIDI: b̂xy = 0.241 , P = 4.5e−19 , n_snps= 152 ; After HEIDI: b̂xy = 0.3 , P = 1.9e−25 , n_snps= 140
Diabetes type 2 (bzy)
SI (bzx)
Outcome (−log10Pzy)
SI (−log10Pzx)
b̂xy (SI −> Diabetes type 2)
No HEIDI: b̂xy = 0.189 , P = 6.7e−08 , n_snps= 77 ; After HEIDI: b̂xy = 0.182 , P = 1.2e−06 , n_snps= 68
Diabetes type 2 (bzy)
FS (bzx)
Outcome (−log10Pzy)
FS (−log10Pzx)
b̂xy (FS −> Diabetes type 2)
No HEIDI: b̂xy = 0.056 , P = 0.021 , n_snps= 65 ; After HEIDI: b̂xy = 0.02 , P = 0.43 , n_snps= 60
Diabetes type 2 (bzy)
CS (bzx)
Outcome (−log10Pzy)
CS (−log10Pzx)
b̂xy (CS −> Diabetes type 2)
No HEIDI: b̂xy = 0.049 , P = 0.4 , n_snps= 8 ; After HEIDI: b̂xy = 0.18 , P = 0.0058 , n_snps= 6
Diabetes type 2 (bzy)
SC (bzx)
Outcome (−log10Pzy)
SC (−log10Pzx)
b̂xy (SC −> Diabetes type 2)
No HEIDI: b̂xy = 0.011 , P = 0.86 , n_snps= 52 ; After HEIDI: b̂xy = −0.449 , P = 3.3e−07 , n_snps= 44
Diabetes type 2 (bzy)
AC (bzx)
Outcome (−log10Pzy)
AC (−log10Pzx)
b̂xy (AC −> Diabetes type 2)
No HEIDI: b̂xy = 0.59 , P = 8.6e−20 , n_snps= 49 ; After HEIDI: b̂xy = −0.067 , P = 0.52 , n_snps= 34
Diabetes type 2 (bzy)
CI (bzx)
Outcome (−log10Pzy)
CI (−log10Pzx)
b̂xy (CI −> Diabetes type 2)
No HEIDI: b̂xy = −0.066 , P = 0.38 , n_snps= 43 ; After HEIDI: b̂xy = −0.406 , P = 0.00011 , n_snps= 35
Diabetes type 2 (bzy)
TI (bzx)
Outcome (−log10Pzy)
TI (−log10Pzx)
b̂xy (TI −> Diabetes type 2)

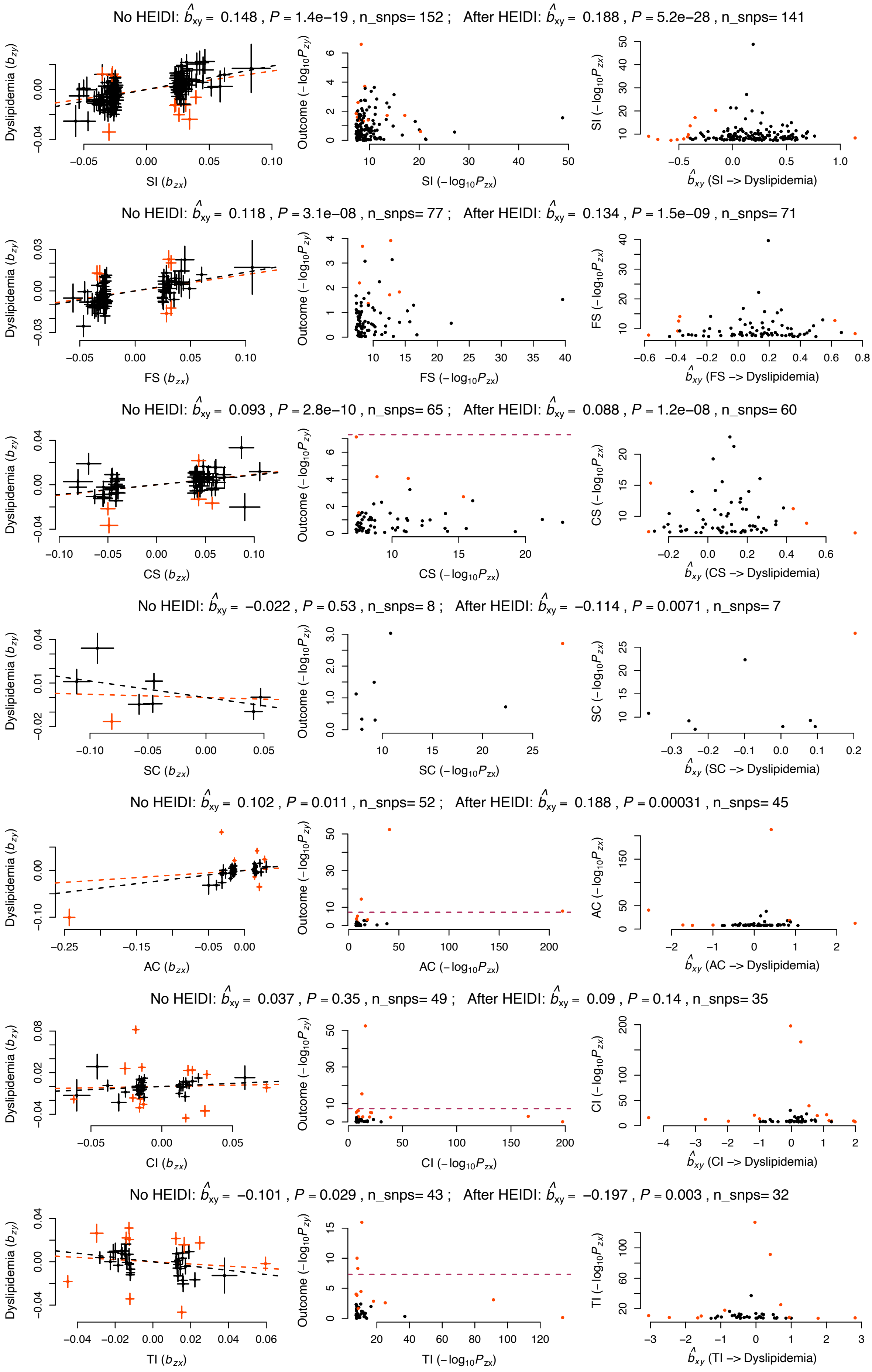
No HEIDI: b̂xy = 0.148 , P = 1.4e−19 , n_snps= 152 ; After HEIDI: b̂xy = 0.188 , P = 5.2e−28 , n_snps= 141
Dyslipidemia (bzy)
SI (bzx)
Outcome (−log10Pzy)
SI (−log10Pzx)
b̂xy (SI −> Dyslipidemia)
No HEIDI: b̂xy = 0.118 , P = 3.1e−08 , n_snps= 77 ; After HEIDI: b̂xy = 0.134 , P = 1.5e−09 , n_snps= 71
FS (bzx)
FS (−log10Pzx)
b̂xy (FS −> Dyslipidemia)
No HEIDI: b̂xy = 0.093 , P = 2.8e−10 , n_snps= 65 ; After HEIDI: b̂xy = 0.088 , P = 1.2e−08 , n_snps= 60
CS (bzx)
CS (−log10Pzx)
b̂xy (CS −> Dyslipidemia)
No HEIDI: b̂xy = −0.022 , P = 0.53 , n_snps= 8 ; After HEIDI: b̂xy = −0.114 , P = 0.0071 , n_snps= 7
SC (bzx)
SC (−log10Pzx)
b̂xy (SC −> Dyslipidemia)
No HEIDI: b̂xy = 0.102 , P = 0.011 , n_snps= 52 ; After HEIDI: b̂xy = 0.188 , P = 0.00031 , n_snps= 45
AC (bzx)
AC (−log10Pzx)
b̂xy (AC −> Dyslipidemia)
No HEIDI: b̂xy = 0.037 , P = 0.35 , n_snps= 49 ; After HEIDI: b̂xy = 0.09 , P = 0.14 , n_snps= 35
CI (bzx)
CI (−log10Pzx)
b̂xy (CI −> Dyslipidemia)
No HEIDI: b̂xy = −0.101 , P = 0.029 , n_snps= 43 ; After HEIDI: b̂xy = −0.197 , P = 0.003 , n_snps= 32
TI (bzx)
TI (−log10Pzx)
b̂xy (TI −> Dyslipidemia)

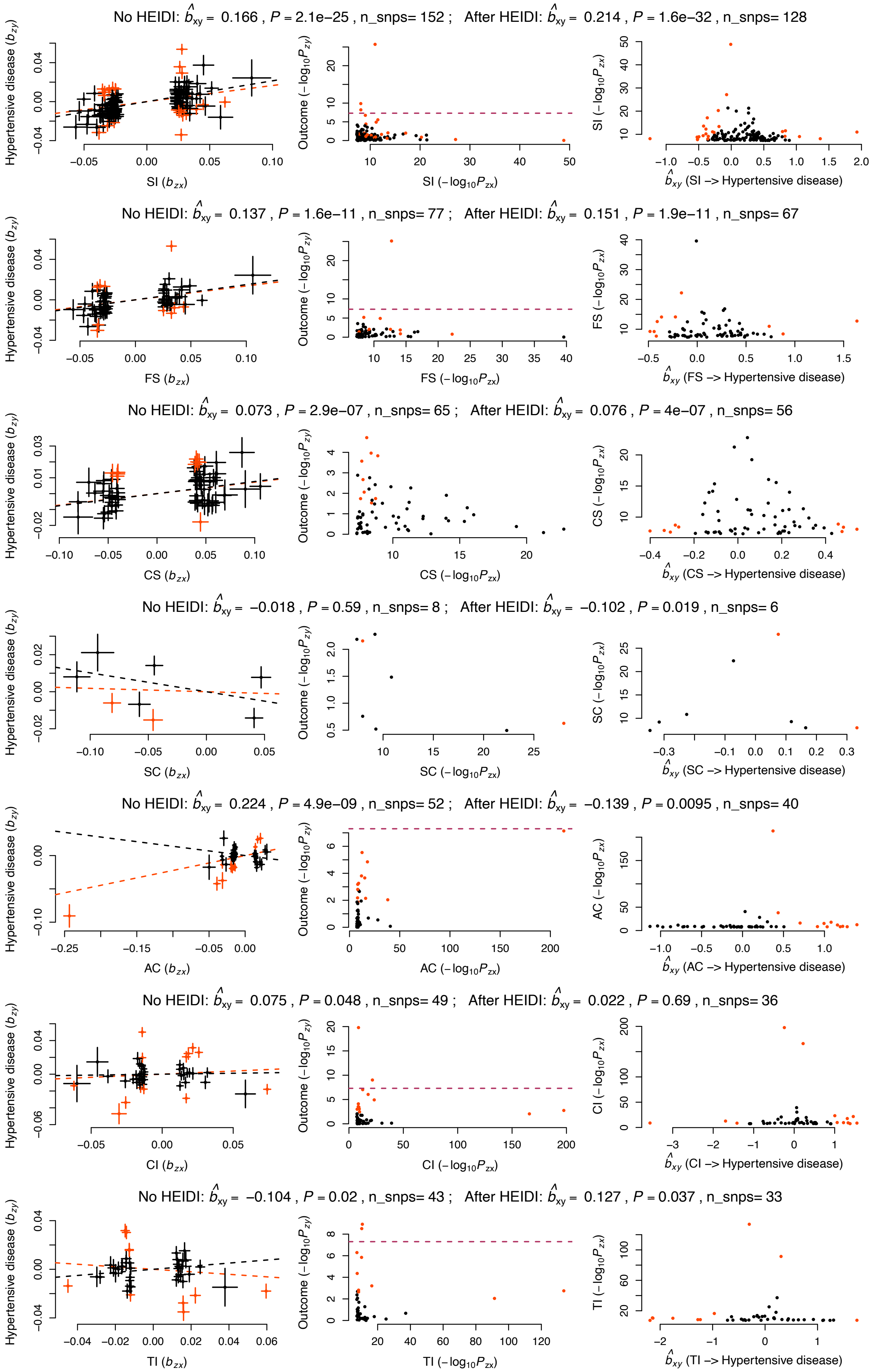

No HEIDI: b̂xy = 0.166 , P = 2.1e−25 , n_snps= 152 ; After HEIDI: b̂xy = 0.214 , P = 1.6e−32 , n_snps= 128
Hypertensive disease (bzy)
SI (bzx)
Outcome (−log10Pzy)
SI (−log10Pzx)
b̂xy (SI −> Hypertensive disease)
No HEIDI: b̂xy = 0.137 , P = 1.6e−11 , n_snps= 77 ; After HEIDI: b̂xy = 0.151 , P = 1.9e−11 , n_snps= 67
FS (bzx)
FS (−log10Pzx)
b̂xy (FS −> Hypertensive disease)
No HEIDI: b̂xy = 0.073 , P = 2.9e−07 , n_snps= 65 ; After HEIDI: b̂xy = 0.076 , P = 4e−07 , n_snps= 56
CS (bzx)
CS (−log10Pzx)
b̂xy (CS −> Hypertensive disease)
No HEIDI: b̂xy = −0.018 , P = 0.59 , n_snps= 8 ; After HEIDI: b̂xy = −0.102 , P = 0.019 , n_snps= 6
SC (bzx)
SC (−log10Pzx)
b̂xy (SC −> Hypertensive disease)
No HEIDI: b̂xy = 0.224 , P = 4.9e−09 , n_snps= 52 ; After HEIDI: b̂xy = −0.139 , P = 0.0095 , n_snps= 40
AC (bzx)
AC (−log10Pzx)
b̂xy (AC −> Hypertensive disease)
No HEIDI: b̂xy = 0.075 , P = 0.048 , n_snps= 49 ; After HEIDI: b̂xy = 0.022 , P = 0.69 , n_snps= 36
CI (bzx)
CI (−log10Pzx)
b̂xy (CI −> Hypertensive disease)
No HEIDI: b̂xy = −0.104 , P = 0.02 , n_snps= 43 ; After HEIDI: b̂xy = 0.127 , P = 0.037 , n_snps= 33
TI (bzx)
TI (−log10Pzx)
b̂xy (TI −> Hypertensive disease)

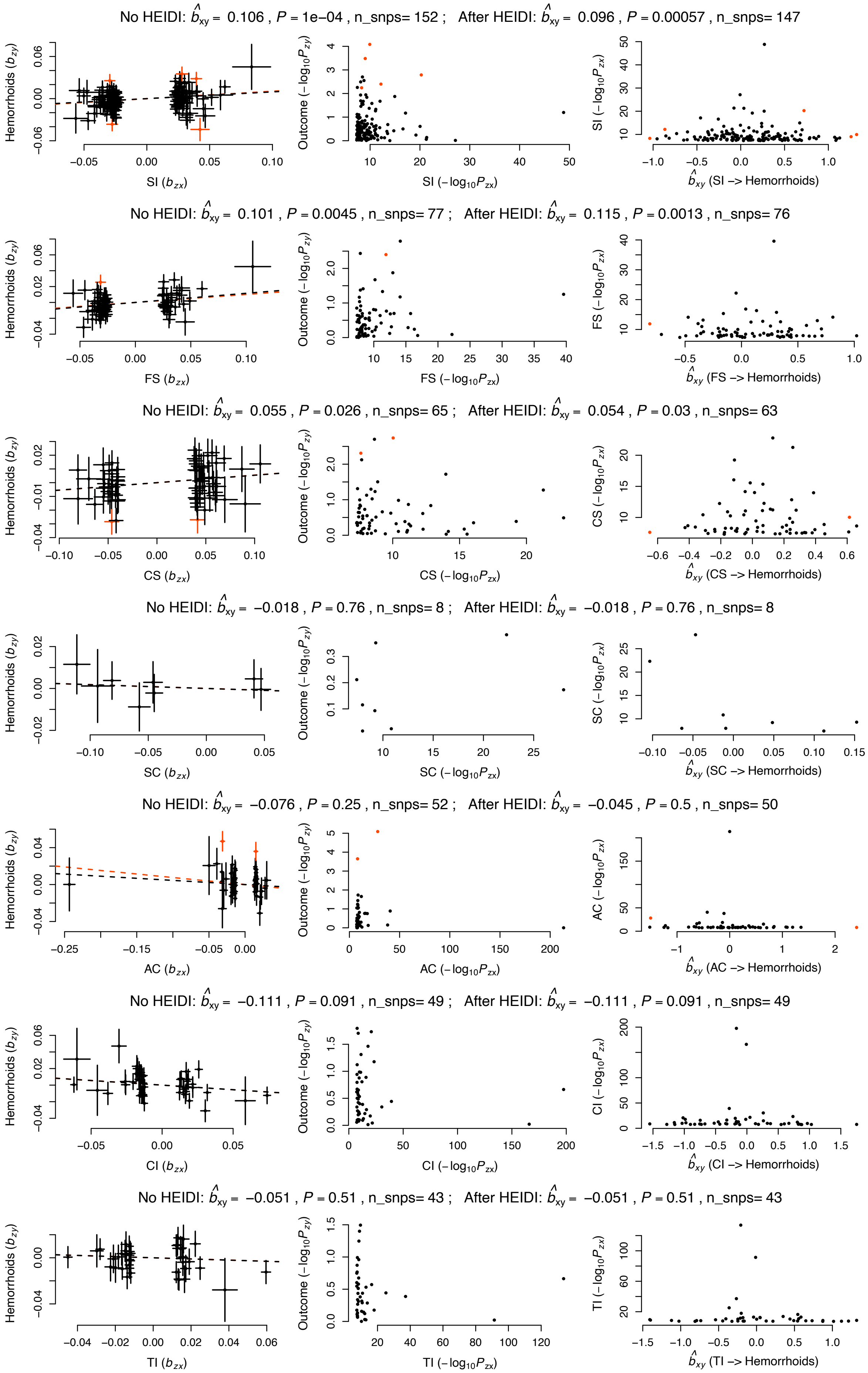
No HEIDI: b̂xy = 0.106 , P = 1e−04 , n_snps= 152 ; After HEIDI: b̂xy = 0.096 , P = 0.00057 , n_snps= 147
Hemorrhoids (bzy)
SI (bzx)
Outcome (−log10Pzy)
SI (−log10Pzx)
b̂xy (SI −> Hemorrhoids)
No HEIDI: b̂xy = 0.101 , P = 0.0045 , n_snps= 77 ; After HEIDI: b̂xy = 0.115 , P = 0.0013 , n_snps= 76
FS (bzx)
FS (−log10Pzx)
b̂xy (FS −> Hemorrhoids)
No HEIDI: b̂xy = 0.055 , P = 0.026 , n_snps= 65 ; After HEIDI: b̂xy = 0.054 , P = 0.03 , n_snps= 63
CS (bzx)
CS (−log10Pzx)
b̂xy (CS −> Hemorrhoids)
No HEIDI: b̂xy = −0.018 , P = 0.76 , n_snps= 8 ; After HEIDI: b̂xy = −0.018 , P = 0.76 , n_snps= 8
SC (bzx)
SC (−log10Pzx)
b̂xy (SC −> Hemorrhoids)
No HEIDI: b̂xy = −0.076 , P = 0.25 , n_snps= 52 ; After HEIDI: b̂xy = −0.045 , P = 0.5 , n_snps= 50
AC (bzx)
AC (−log10Pzx)
b̂xy (AC −> Hemorrhoids)
No HEIDI: b̂xy = −0.111 , P = 0.091 , n_snps= 49 ; After HEIDI: b̂xy = −0.111 , P = 0.091 , n_snps= 49
CI (bzx)
CI (−log10Pzx)
b̂xy (CI −> Hemorrhoids)
No HEIDI: b̂xy = −0.051 , P = 0.51 , n_snps= 43 ; After HEIDI: b̂xy = −0.051 , P = 0.51 , n_snps= 43
TI (bzx)
TI (−log10Pzx)
b̂xy (TI −> Hemorrhoids)

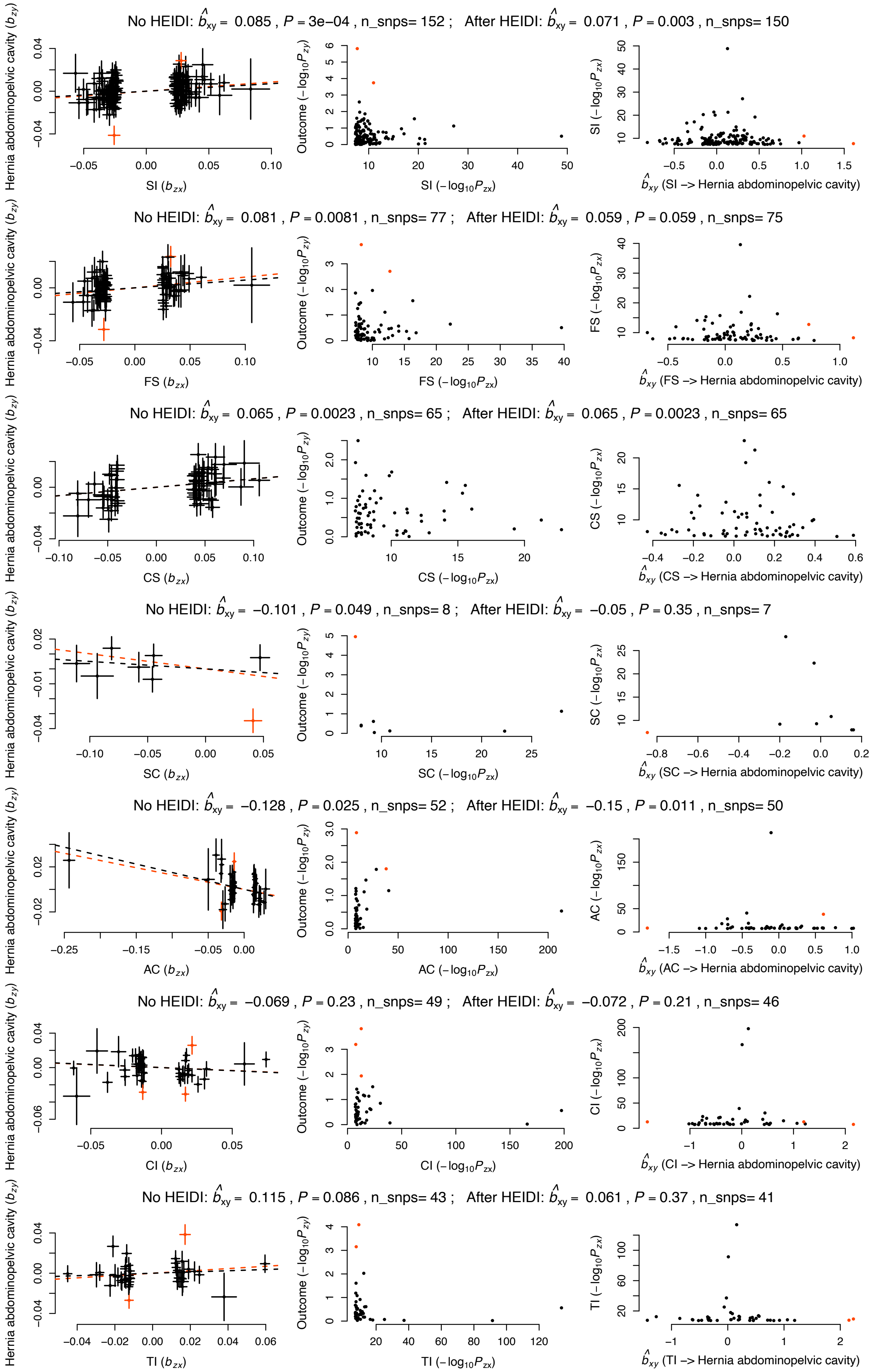
No HEIDI: $\hat{b}_{xy}$ = 0.085 , P = 3e−04 , n_snps= 152 ; After HEIDI: $\hat{b}_{xy}$ = 0.071 , P = 0.003 , n_snps= 150
Hernia abdominopelvic cavity (b_{zy})
SI (b_{zx})
Outcome ($-\log_{10}P_{zy}$)
SI ($-\log_{10}P_{zx}$)
$\hat{b}_{xy}$ (SI -> Hernia abdominopelvic cavity)
No HEIDI: $\hat{b}_{xy}$ = 0.081 , P = 0.0081 , n_snps= 77 ; After HEIDI: $\hat{b}_{xy}$ = 0.059 , P = 0.059 , n_snps= 75
FS (b_{zx})
FS ($-\log_{10}P_{zx}$)
$\hat{b}_{xy}$ (FS -> Hernia abdominopelvic cavity)
No HEIDI: $\hat{b}_{xy}$ = 0.065 , P = 0.0023 , n_snps= 65 ; After HEIDI: $\hat{b}_{xy}$ = 0.065 , P = 0.0023 , n_snps= 65
CS (b_{zx})
CS ($-\log_{10}P_{zx}$)
$\hat{b}_{xy}$ (CS -> Hernia abdominopelvic cavity)
No HEIDI: $\hat{b}_{xy}$ = −0.101 , P = 0.049 , n_snps= 8 ; After HEIDI: $\hat{b}_{xy}$ = −0.05 , P = 0.35 , n_snps= 7
SC (b_{zx})
SC ($-\log_{10}P_{zx}$)
$\hat{b}_{xy}$ (SC -> Hernia abdominopelvic cavity)
No HEIDI: $\hat{b}_{xy}$ = −0.128 , P = 0.025 , n_snps= 52 ; After HEIDI: $\hat{b}_{xy}$ = −0.15 , P = 0.011 , n_snps= 50
AC (b_{zx})
AC ($-\log_{10}P_{zx}$)
$\hat{b}_{xy}$ (AC -> Hernia abdominopelvic cavity)
No HEIDI: $\hat{b}_{xy}$ = −0.069 , P = 0.23 , n_snps= 49 ; After HEIDI: $\hat{b}_{xy}$ = −0.072 , P = 0.21 , n_snps= 46
CI (b_{zx})
CI ($-\log_{10}P_{zx}$)
$\hat{b}_{xy}$ (CI -> Hernia abdominopelvic cavity)
No HEIDI: $\hat{b}_{xy}$ = 0.115 , P = 0.086 , n_snps= 43 ; After HEIDI: $\hat{b}_{xy}$ = 0.061 , P = 0.37 , n_snps= 41
TI (b_{zx})
TI ($-\log_{10}P_{zx}$)
$\hat{b}_{xy}$ (TI -> Hernia abdominopelvic cavity)

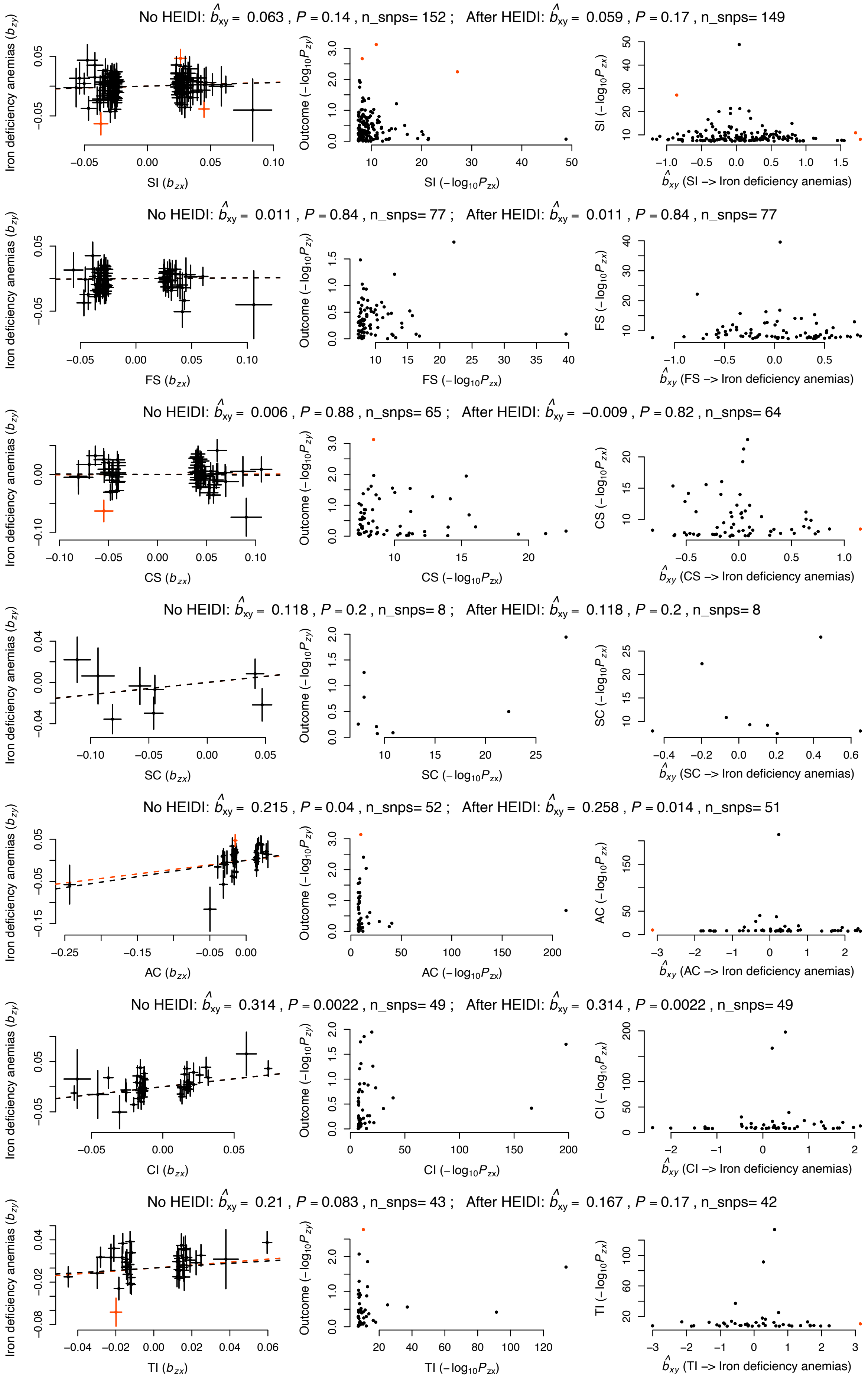

No HEIDI: $\hat{b}_{xy}$ = 0.063 , P = 0.14 , n_snps= 152 ; After HEIDI: $\hat{b}_{xy}$ = 0.059 , P = 0.17 , n_snps= 149
Iron deficiency anemias (b_{zy})
SI (b_{zx})
Outcome ($-\log_{10}P_{zy}$)
SI ($-\log_{10}P_{zx}$)
$\hat{b}_{xy}$ (SI -> Iron deficiency anemias)
No HEIDI: $\hat{b}_{xy}$ = 0.011 , P = 0.84 , n_snps= 77 ; After HEIDI: $\hat{b}_{xy}$ = 0.011 , P = 0.84 , n_snps= 77
FS (b_{zx})
FS ($-\log_{10}P_{zx}$)
$\hat{b}_{xy}$ (FS -> Iron deficiency anemias)
No HEIDI: $\hat{b}_{xy}$ = 0.006 , P = 0.88 , n_snps= 65 ; After HEIDI: $\hat{b}_{xy}$ = −0.009 , P = 0.82 , n_snps= 64
CS (b_{zx})
CS ($-\log_{10}P_{zx}$)
$\hat{b}_{xy}$ (CS -> Iron deficiency anemias)
No HEIDI: $\hat{b}_{xy}$ = 0.118 , P = 0.2 , n_snps= 8 ; After HEIDI: $\hat{b}_{xy}$ = 0.118 , P = 0.2 , n_snps= 8
SC (b_{zx})
SC ($-\log_{10}P_{zx}$)
$\hat{b}_{xy}$ (SC -> Iron deficiency anemias)
No HEIDI: $\hat{b}_{xy}$ = 0.215 , P = 0.04 , n_snps= 52 ; After HEIDI: $\hat{b}_{xy}$ = 0.258 , P = 0.014 , n_snps= 51
AC (b_{zx})
AC ($-\log_{10}P_{zx}$)
$\hat{b}_{xy}$ (AC -> Iron deficiency anemias)
No HEIDI: $\hat{b}_{xy}$ = 0.314 , P = 0.0022 , n_snps= 49 ; After HEIDI: $\hat{b}_{xy}$ = 0.314 , P = 0.0022 , n_snps= 49
CI (b_{zx})
CI ($-\log_{10}P_{zx}$)
$\hat{b}_{xy}$ (CI -> Iron deficiency anemias)
No HEIDI: $\hat{b}_{xy}$ = 0.21 , P = 0.083 , n_snps= 43 ; After HEIDI: $\hat{b}_{xy}$ = 0.167 , P = 0.17 , n_snps= 42
TI (b_{zx})
TI ($-\log_{10}P_{zx}$)
$\hat{b}_{xy}$ (TI -> Iron deficiency anemias)

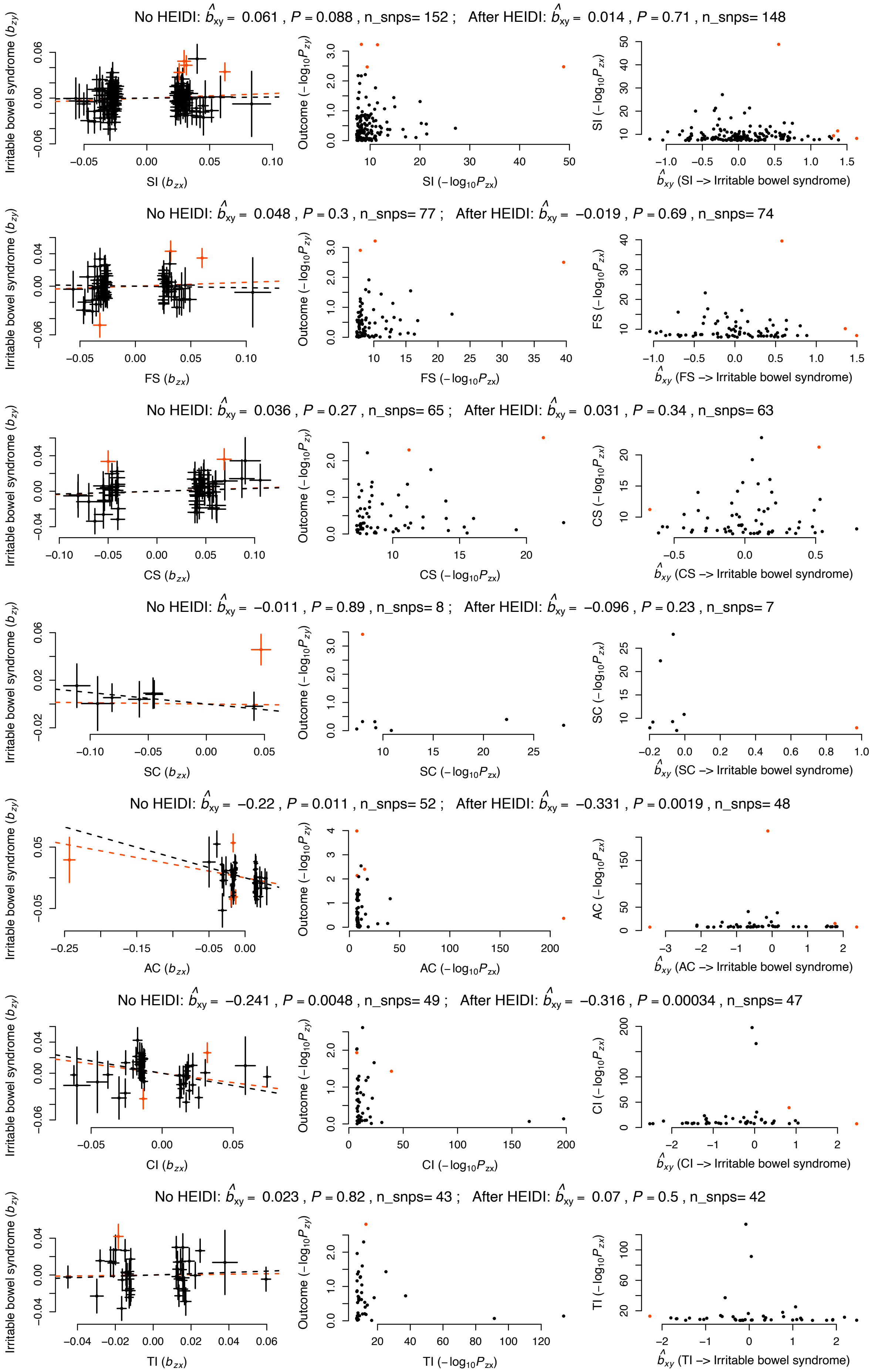
No HEIDI: $\hat{b}_{xy}$ = 0.061 , P = 0.088 , n_snps= 152 ; After HEIDI: $\hat{b}_{xy}$ = 0.014 , P = 0.71 , n_snps= 148
Irritable bowel syndrome (b_{zy})
SI (b_{zx})
Outcome ($-\log_{10}P_{zy}$)
SI ($-\log_{10}P_{zx}$)
$\hat{b}_{xy}$ (SI -> Irritable bowel syndrome)
No HEIDI: $\hat{b}_{xy}$ = 0.048 , P = 0.3 , n_snps= 77 ; After HEIDI: $\hat{b}_{xy}$ = −0.019 , P = 0.69 , n_snps= 74
Irritable bowel syndrome (b_{zy})
FS (b_{zx})
Outcome ($-\log_{10}P_{zy}$)
FS ($-\log_{10}P_{zx}$)
$\hat{b}_{xy}$ (FS -> Irritable bowel syndrome)
No HEIDI: $\hat{b}_{xy}$ = 0.036 , P = 0.27 , n_snps= 65 ; After HEIDI: $\hat{b}_{xy}$ = 0.031 , P = 0.34 , n_snps= 63
Irritable bowel syndrome (b_{zy})
CS (b_{zx})
Outcome ($-\log_{10}P_{zy}$)
CS ($-\log_{10}P_{zx}$)
$\hat{b}_{xy}$ (CS -> Irritable bowel syndrome)
No HEIDI: $\hat{b}_{xy}$ = −0.011 , P = 0.89 , n_snps= 8 ; After HEIDI: $\hat{b}_{xy}$ = −0.096 , P = 0.23 , n_snps= 7
Irritable bowel syndrome (b_{zy})
SC (b_{zx})
Outcome ($-\log_{10}P_{zy}$)
SC ($-\log_{10}P_{zx}$)
$\hat{b}_{xy}$ (SC -> Irritable bowel syndrome)
No HEIDI: $\hat{b}_{xy}$ = −0.22 , P = 0.011 , n_snps= 52 ; After HEIDI: $\hat{b}_{xy}$ = −0.331 , P = 0.0019 , n_snps= 48
Irritable bowel syndrome (b_{zy})
AC (b_{zx})
Outcome ($-\log_{10}P_{zy}$)
AC ($-\log_{10}P_{zx}$)
$\hat{b}_{xy}$ (AC -> Irritable bowel syndrome)
No HEIDI: $\hat{b}_{xy}$ = −0.241 , P = 0.0048 , n_snps= 49 ; After HEIDI: $\hat{b}_{xy}$ = −0.316 , P = 0.00034 , n_snps= 47
Irritable bowel syndrome (b_{zy})
CI (b_{zx})
Outcome ($-\log_{10}P_{zy}$)
CI ($-\log_{10}P_{zx}$)
$\hat{b}_{xy}$ (CI -> Irritable bowel syndrome)
No HEIDI: $\hat{b}_{xy}$ = 0.023 , P = 0.82 , n_snps= 43 ; After HEIDI: $\hat{b}_{xy}$ = 0.07 , P = 0.5 , n_snps= 42
Irritable bowel syndrome (b_{zy})
TI (b_{zx})
Outcome ($-\log_{10}P_{zy}$)
TI ($-\log_{10}P_{zx}$)
$\hat{b}_{xy}$ (TI -> Irritable bowel syndrome)

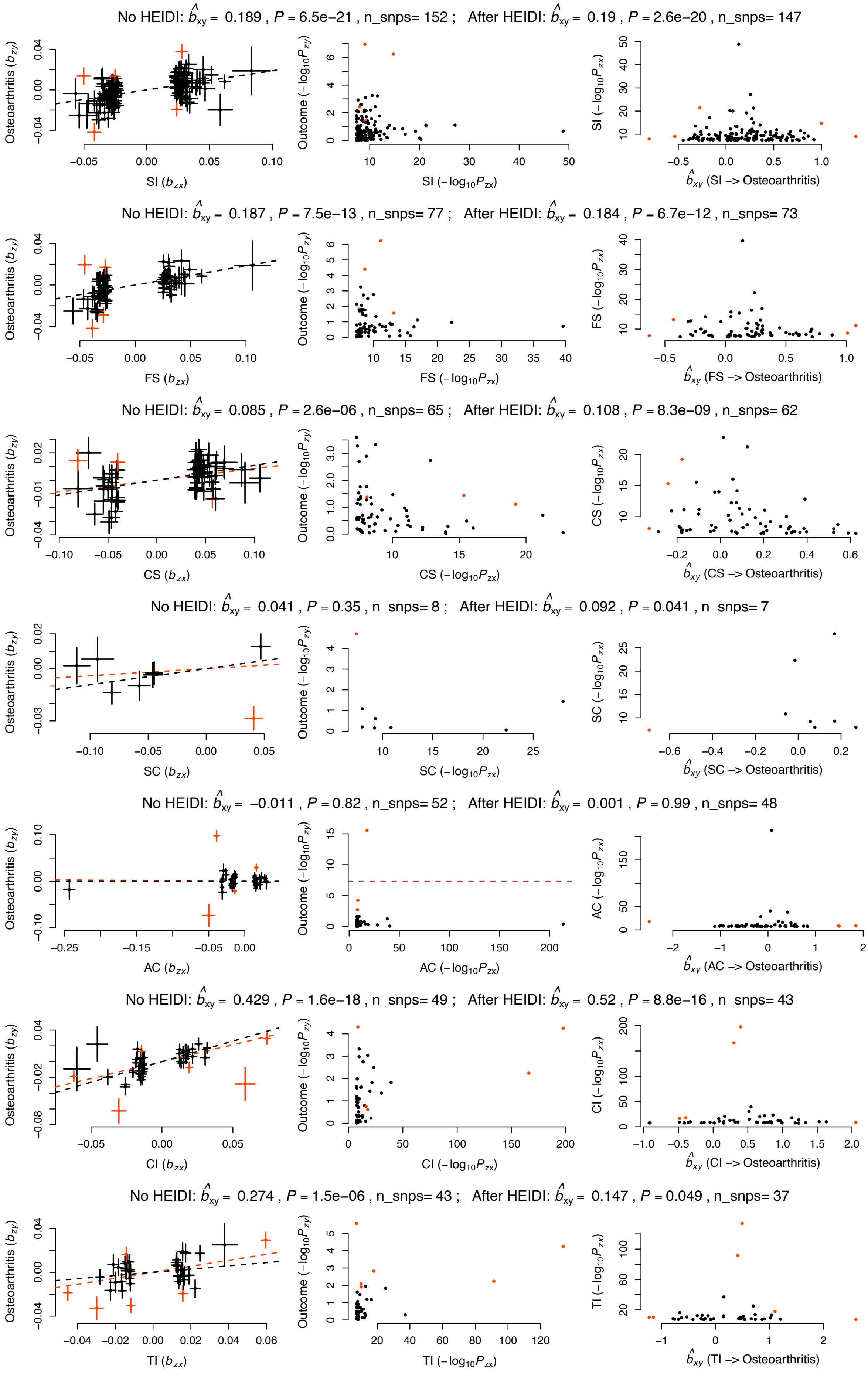

No HEIDI: $\hat{b}_{xy}$ = 0.189 , P = 6.5e−21 , n_snps= 152 ; After HEIDI: $\hat{b}_{xy}$ = 0.19 , P = 2.6e−20 , n_snps= 147
Osteoarthritis (b_{zy})
SI (b_{zx})
Outcome ($-\log_{10}P_{zy}$)
SI ($-\log_{10}P_{zx}$)
$\hat{b}_{xy}$ (SI −> Osteoarthritis)
No HEIDI: $\hat{b}_{xy}$ = 0.187 , P = 7.5e−13 , n_snps= 77 ; After HEIDI: $\hat{b}_{xy}$ = 0.184 , P = 6.7e−12 , n_snps= 73
Osteoarthritis (b_{zy})
FS (b_{zx})
Outcome ($-\log_{10}P_{zy}$)
FS ($-\log_{10}P_{zx}$)
$\hat{b}_{xy}$ (FS −> Osteoarthritis)
No HEIDI: $\hat{b}_{xy}$ = 0.085 , P = 2.6e−06 , n_snps= 65 ; After HEIDI: $\hat{b}_{xy}$ = 0.108 , P = 8.3e−09 , n_snps= 62
Osteoarthritis (b_{zy})
CS (b_{zx})
Outcome ($-\log_{10}P_{zy}$)
CS ($-\log_{10}P_{zx}$)
$\hat{b}_{xy}$ (CS −> Osteoarthritis)
No HEIDI: $\hat{b}_{xy}$ = 0.041 , P = 0.35 , n_snps= 8 ; After HEIDI: $\hat{b}_{xy}$ = 0.092 , P = 0.041 , n_snps= 7
Osteoarthritis (b_{zy})
SC (b_{zx})
Outcome ($-\log_{10}P_{zy}$)
SC ($-\log_{10}P_{zx}$)
$\hat{b}_{xy}$ (SC −> Osteoarthritis)
No HEIDI: $\hat{b}_{xy}$ = −0.011 , P = 0.82 , n_snps= 52 ; After HEIDI: $\hat{b}_{xy}$ = 0.001 , P = 0.99 , n_snps= 48
Osteoarthritis (b_{zy})
AC (b_{zx})
Outcome ($-\log_{10}P_{zy}$)
AC ($-\log_{10}P_{zx}$)
$\hat{b}_{xy}$ (AC −> Osteoarthritis)
No HEIDI: $\hat{b}_{xy}$ = 0.429 , P = 1.6e−18 , n_snps= 49 ; After HEIDI: $\hat{b}_{xy}$ = 0.52 , P = 8.8e−16 , n_snps= 43
Osteoarthritis (b_{zy})
CI (b_{zx})
Outcome ($-\log_{10}P_{zy}$)
CI ($-\log_{10}P_{zx}$)
$\hat{b}_{xy}$ (CI −> Osteoarthritis)
No HEIDI: $\hat{b}_{xy}$ = 0.274 , P = 1.5e−06 , n_snps= 43 ; After HEIDI: $\hat{b}_{xy}$ = 0.147 , P = 0.049 , n_snps= 37
Osteoarthritis (b_{zy})
TI (b_{zx})
Outcome ($-\log_{10}P_{zy}$)
TI ($-\log_{10}P_{zx}$)
$\hat{b}_{xy}$ (TI −> Osteoarthritis)

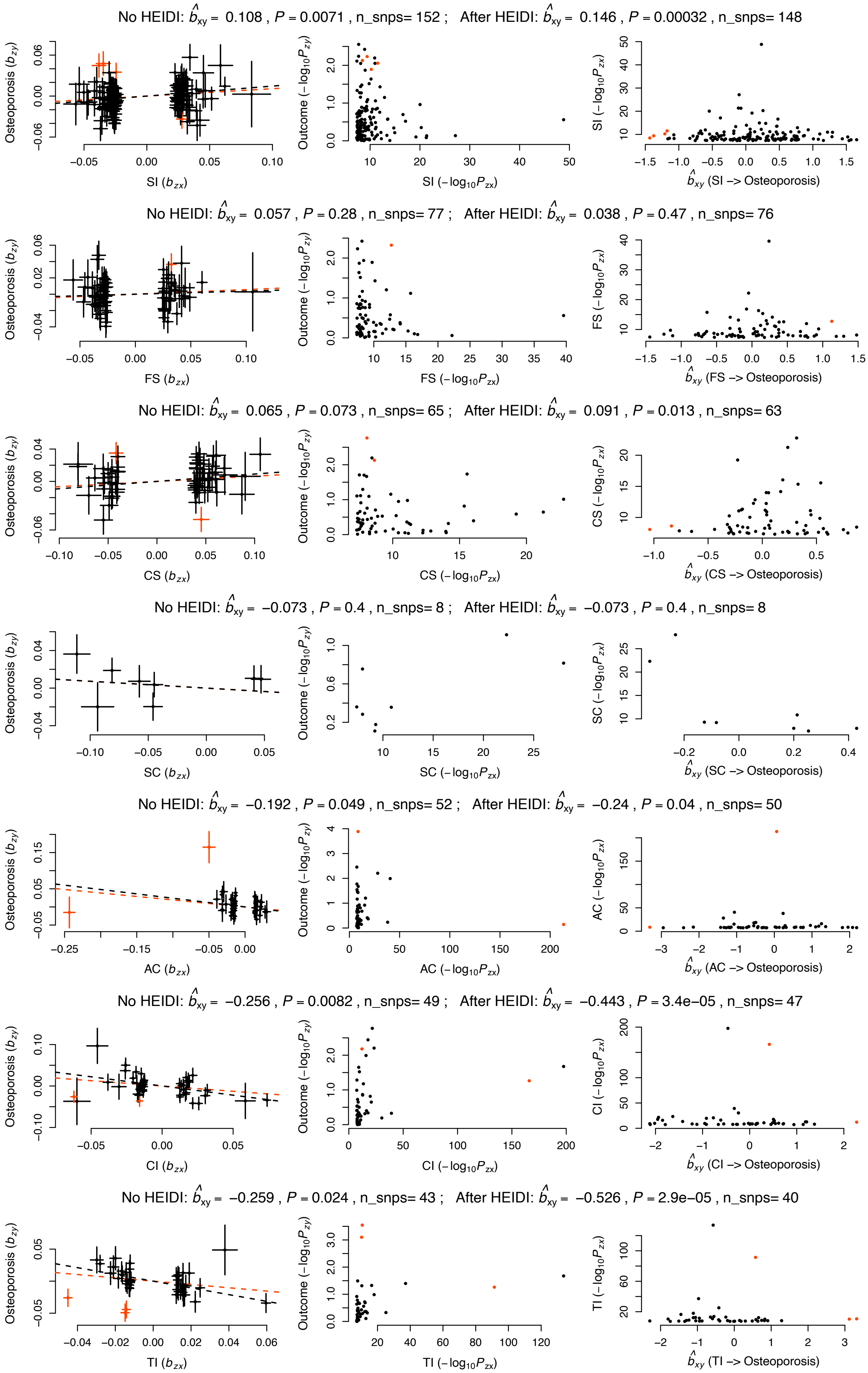
No HEIDI: b̂xy = 0.108 , P = 0.0071 , n_snps= 152 ; After HEIDI: b̂xy = 0.146 , P = 0.00032 , n_snps= 148
Osteoporosis (bzy)
SI (bzx)
Outcome (−log10Pzy)
SI (−log10Pzx)
b̂xy (SI −> Osteoporosis)
No HEIDI: b̂xy = 0.057 , P = 0.28 , n_snps= 77 ; After HEIDI: b̂xy = 0.038 , P = 0.47 , n_snps= 76
FS (bzx)
FS (−log10Pzx)
b̂xy (FS −> Osteoporosis)
No HEIDI: b̂xy = 0.065 , P = 0.073 , n_snps= 65 ; After HEIDI: b̂xy = 0.091 , P = 0.013 , n_snps= 63
CS (bzx)
CS (−log10Pzx)
b̂xy (CS −> Osteoporosis)
No HEIDI: b̂xy = −0.073 , P = 0.4 , n_snps= 8 ; After HEIDI: b̂xy = −0.073 , P = 0.4 , n_snps= 8
SC (bzx)
SC (−log10Pzx)
b̂xy (SC −> Osteoporosis)
No HEIDI: b̂xy = −0.192 , P = 0.049 , n_snps= 52 ; After HEIDI: b̂xy = −0.24 , P = 0.04 , n_snps= 50
AC (bzx)
AC (−log10Pzx)
b̂xy (AC −> Osteoporosis)
No HEIDI: b̂xy = −0.256 , P = 0.0082 , n_snps= 49 ; After HEIDI: b̂xy = −0.443 , P = 3.4e−05 , n_snps= 47
CI (bzx)
CI (−log10Pzx)
b̂xy (CI −> Osteoporosis)
No HEIDI: b̂xy = −0.259 , P = 0.024 , n_snps= 43 ; After HEIDI: b̂xy = −0.526 , P = 2.9e−05 , n_snps= 40
TI (bzx)
TI (−log10Pzx)
b̂xy (TI −> Osteoporosis)

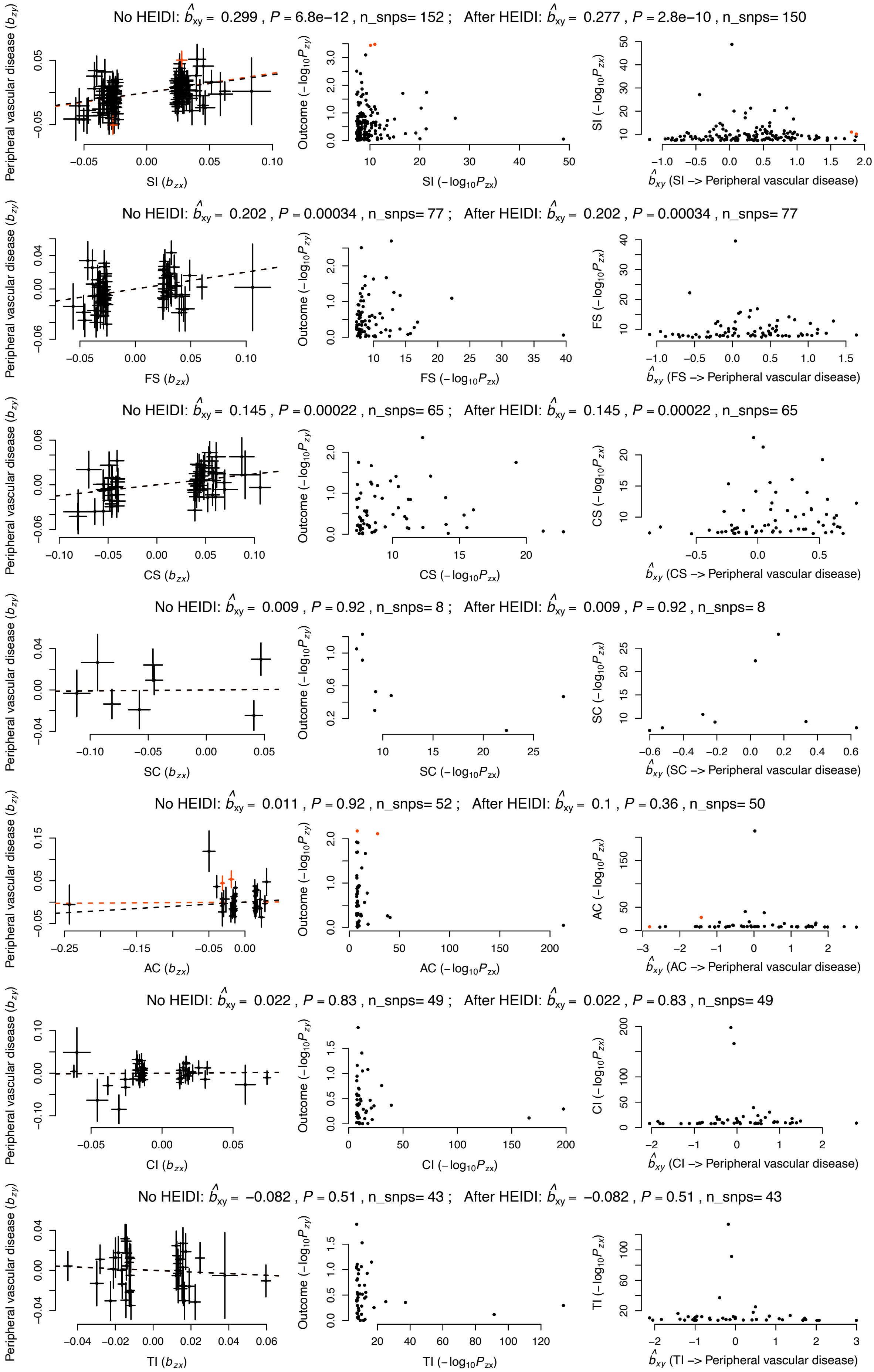
No HEIDI: $\hat{b}_{xy}$ = 0.299 , P = 6.8e−12 , n_snps= 152 ; After HEIDI: $\hat{b}_{xy}$ = 0.277 , P = 2.8e−10 , n_snps= 150
Peripheral vascular disease (b_{zy})
SI (b_{zx})
Outcome ($-\log_{10}P_{zy}$)
SI ($-\log_{10}P_{zx}$)
$\hat{b}_{xy}$ (SI –> Peripheral vascular disease)
No HEIDI: $\hat{b}_{xy}$ = 0.202 , P = 0.00034 , n_snps= 77 ; After HEIDI: $\hat{b}_{xy}$ = 0.202 , P = 0.00034 , n_snps= 77
Peripheral vascular disease (b_{zy})
FS (b_{zx})
Outcome ($-\log_{10}P_{zy}$)
FS ($-\log_{10}P_{zx}$)
$\hat{b}_{xy}$ (FS –> Peripheral vascular disease)
No HEIDI: $\hat{b}_{xy}$ = 0.145 , P = 0.00022 , n_snps= 65 ; After HEIDI: $\hat{b}_{xy}$ = 0.145 , P = 0.00022 , n_snps= 65
Peripheral vascular disease (b_{zy})
CS (b_{zx})
Outcome ($-\log_{10}P_{zy}$)
CS ($-\log_{10}P_{zx}$)
$\hat{b}_{xy}$ (CS –> Peripheral vascular disease)
No HEIDI: $\hat{b}_{xy}$ = 0.009 , P = 0.92 , n_snps= 8 ; After HEIDI: $\hat{b}_{xy}$ = 0.009 , P = 0.92 , n_snps= 8
Peripheral vascular disease (b_{zy})
SC (b_{zx})
Outcome ($-\log_{10}P_{zy}$)
SC ($-\log_{10}P_{zx}$)
$\hat{b}_{xy}$ (SC –> Peripheral vascular disease)
No HEIDI: $\hat{b}_{xy}$ = 0.011 , P = 0.92 , n_snps= 52 ; After HEIDI: $\hat{b}_{xy}$ = 0.1 , P = 0.36 , n_snps= 50
Peripheral vascular disease (b_{zy})
AC (b_{zx})
Outcome ($-\log_{10}P_{zy}$)
AC ($-\log_{10}P_{zx}$)
$\hat{b}_{xy}$ (AC –> Peripheral vascular disease)
No HEIDI: $\hat{b}_{xy}$ = 0.022 , P = 0.83 , n_snps= 49 ; After HEIDI: $\hat{b}_{xy}$ = 0.022 , P = 0.83 , n_snps= 49
Peripheral vascular disease (b_{zy})
CI (b_{zx})
Outcome ($-\log_{10}P_{zy}$)
CI ($-\log_{10}P_{zx}$)
$\hat{b}_{xy}$ (CI –> Peripheral vascular disease)
No HEIDI: $\hat{b}_{xy}$ = −0.082 , P = 0.51 , n_snps= 43 ; After HEIDI: $\hat{b}_{xy}$ = −0.082 , P = 0.51 , n_snps= 43
Peripheral vascular disease (b_{zy})
TI (b_{zx})
Outcome ($-\log_{10}P_{zy}$)
TI ($-\log_{10}P_{zx}$)
$\hat{b}_{xy}$ (TI –> Peripheral vascular disease)

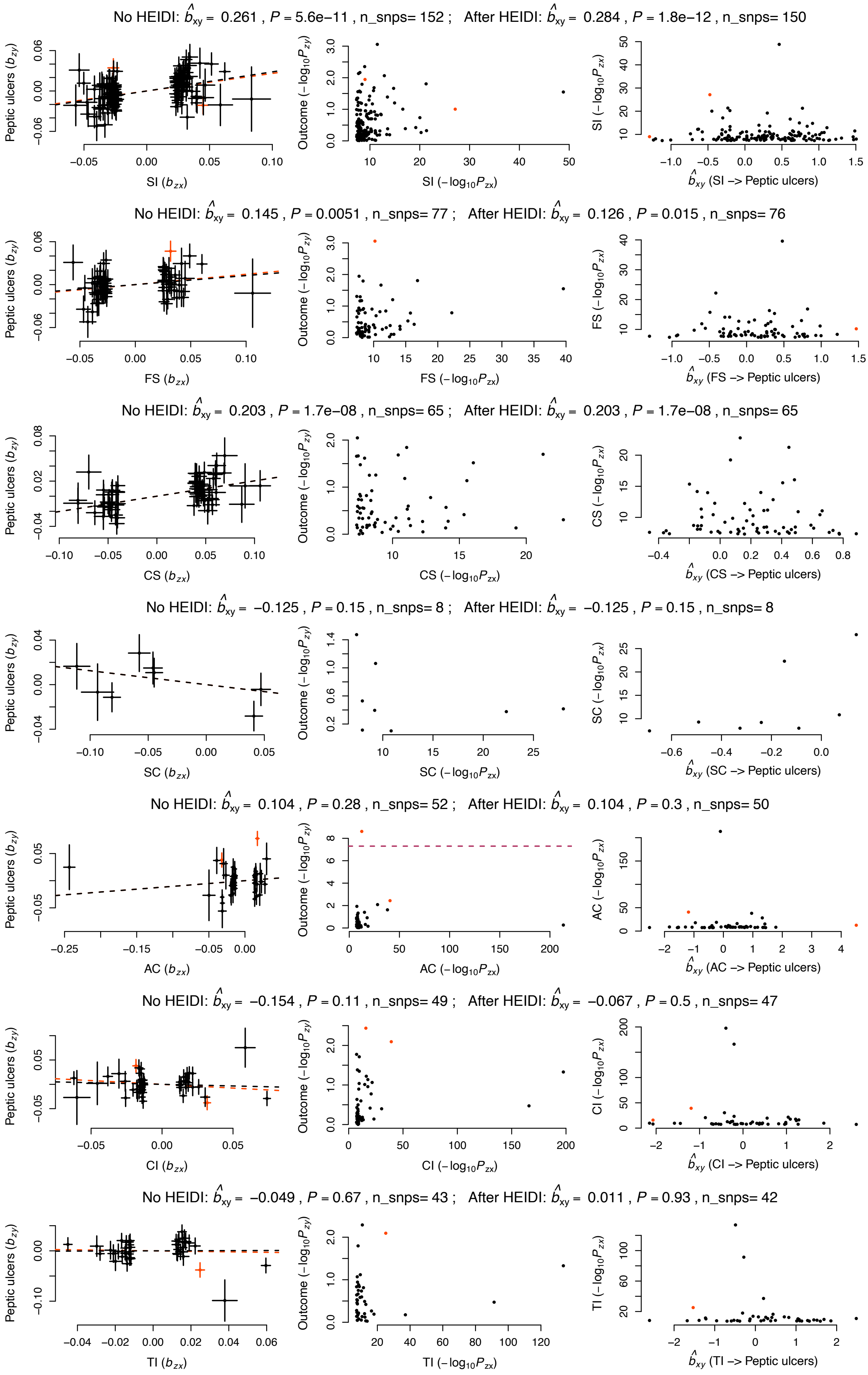
No HEIDI: b̂xy = 0.261 , P = 5.6e−11 , n_snps= 152 ; After HEIDI: b̂xy = 0.284 , P = 1.8e−12 , n_snps= 150
Peptic ulcers (bzy)
SI (bzx)
Outcome (−log10Pzy)
SI (−log10Pzx)
b̂xy (SI −> Peptic ulcers)
No HEIDI: b̂xy = 0.145 , P = 0.0051 , n_snps= 77 ; After HEIDI: b̂xy = 0.126 , P = 0.015 , n_snps= 76
FS (bzx)
FS (−log10Pzx)
b̂xy (FS −> Peptic ulcers)
No HEIDI: b̂xy = 0.203 , P = 1.7e−08 , n_snps= 65 ; After HEIDI: b̂xy = 0.203 , P = 1.7e−08 , n_snps= 65
CS (bzx)
CS (−log10Pzx)
b̂xy (CS −> Peptic ulcers)
No HEIDI: b̂xy = −0.125 , P = 0.15 , n_snps= 8 ; After HEIDI: b̂xy = −0.125 , P = 0.15 , n_snps= 8
SC (bzx)
SC (−log10Pzx)
b̂xy (SC −> Peptic ulcers)
No HEIDI: b̂xy = 0.104 , P = 0.28 , n_snps= 52 ; After HEIDI: b̂xy = 0.104 , P = 0.3 , n_snps= 50
AC (bzx)
AC (−log10Pzx)
b̂xy (AC −> Peptic ulcers)
No HEIDI: b̂xy = −0.154 , P = 0.11 , n_snps= 49 ; After HEIDI: b̂xy = −0.067 , P = 0.5 , n_snps= 47
CI (bzx)
CI (−log10Pzx)
b̂xy (CI −> Peptic ulcers)
No HEIDI: b̂xy = −0.049 , P = 0.67 , n_snps= 43 ; After HEIDI: b̂xy = 0.011 , P = 0.93 , n_snps= 42
TI (bzx)
TI (−log10Pzx)
b̂xy (TI −> Peptic ulcers)

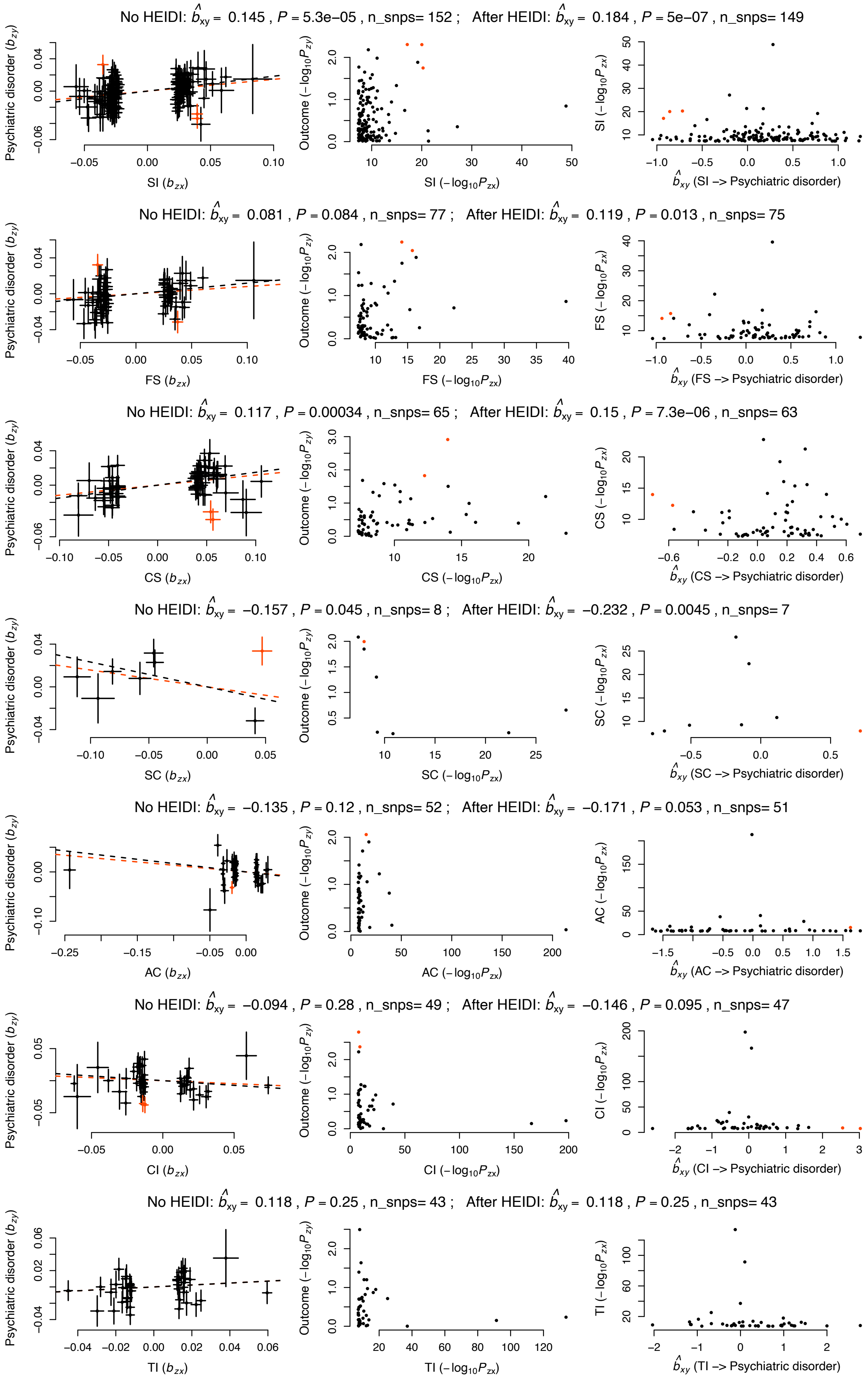

No HEIDI: $\hat{b}_{xy}$ = 0.145 , P = 5.3e−05 , n_snps= 152 ; After HEIDI: $\hat{b}_{xy}$ = 0.184 , P = 5e−07 , n_snps= 149
Psychiatric disorder (b_{zy})
SI (b_{zx})
Outcome ($-\log_{10}P_{zy}$)
SI ($-\log_{10}P_{zx}$)
$\hat{b}_{xy}$ (SI −> Psychiatric disorder)
No HEIDI: $\hat{b}_{xy}$ = 0.081 , P = 0.084 , n_snps= 77 ; After HEIDI: $\hat{b}_{xy}$ = 0.119 , P = 0.013 , n_snps= 75
FS (b_{zx})
FS ($-\log_{10}P_{zx}$)
$\hat{b}_{xy}$ (FS −> Psychiatric disorder)
No HEIDI: $\hat{b}_{xy}$ = 0.117 , P = 0.00034 , n_snps= 65 ; After HEIDI: $\hat{b}_{xy}$ = 0.15 , P = 7.3e−06 , n_snps= 63
CS (b_{zx})
CS ($-\log_{10}P_{zx}$)
$\hat{b}_{xy}$ (CS −> Psychiatric disorder)
No HEIDI: $\hat{b}_{xy}$ = −0.157 , P = 0.045 , n_snps= 8 ; After HEIDI: $\hat{b}_{xy}$ = −0.232 , P = 0.0045 , n_snps= 7
SC (b_{zx})
SC ($-\log_{10}P_{zx}$)
$\hat{b}_{xy}$ (SC −> Psychiatric disorder)
No HEIDI: $\hat{b}_{xy}$ = −0.135 , P = 0.12 , n_snps= 52 ; After HEIDI: $\hat{b}_{xy}$ = −0.171 , P = 0.053 , n_snps= 51
AC (b_{zx})
AC ($-\log_{10}P_{zx}$)
$\hat{b}_{xy}$ (AC −> Psychiatric disorder)
No HEIDI: $\hat{b}_{xy}$ = −0.094 , P = 0.28 , n_snps= 49 ; After HEIDI: $\hat{b}_{xy}$ = −0.146 , P = 0.095 , n_snps= 47
CI (b_{zx})
CI ($-\log_{10}P_{zx}$)
$\hat{b}_{xy}$ (CI −> Psychiatric disorder)
No HEIDI: $\hat{b}_{xy}$ = 0.118 , P = 0.25 , n_snps= 43 ; After HEIDI: $\hat{b}_{xy}$ = 0.118 , P = 0.25 , n_snps= 43
TI (b_{zx})
TI ($-\log_{10}P_{zx}$)
$\hat{b}_{xy}$ (TI −> Psychiatric disorder)

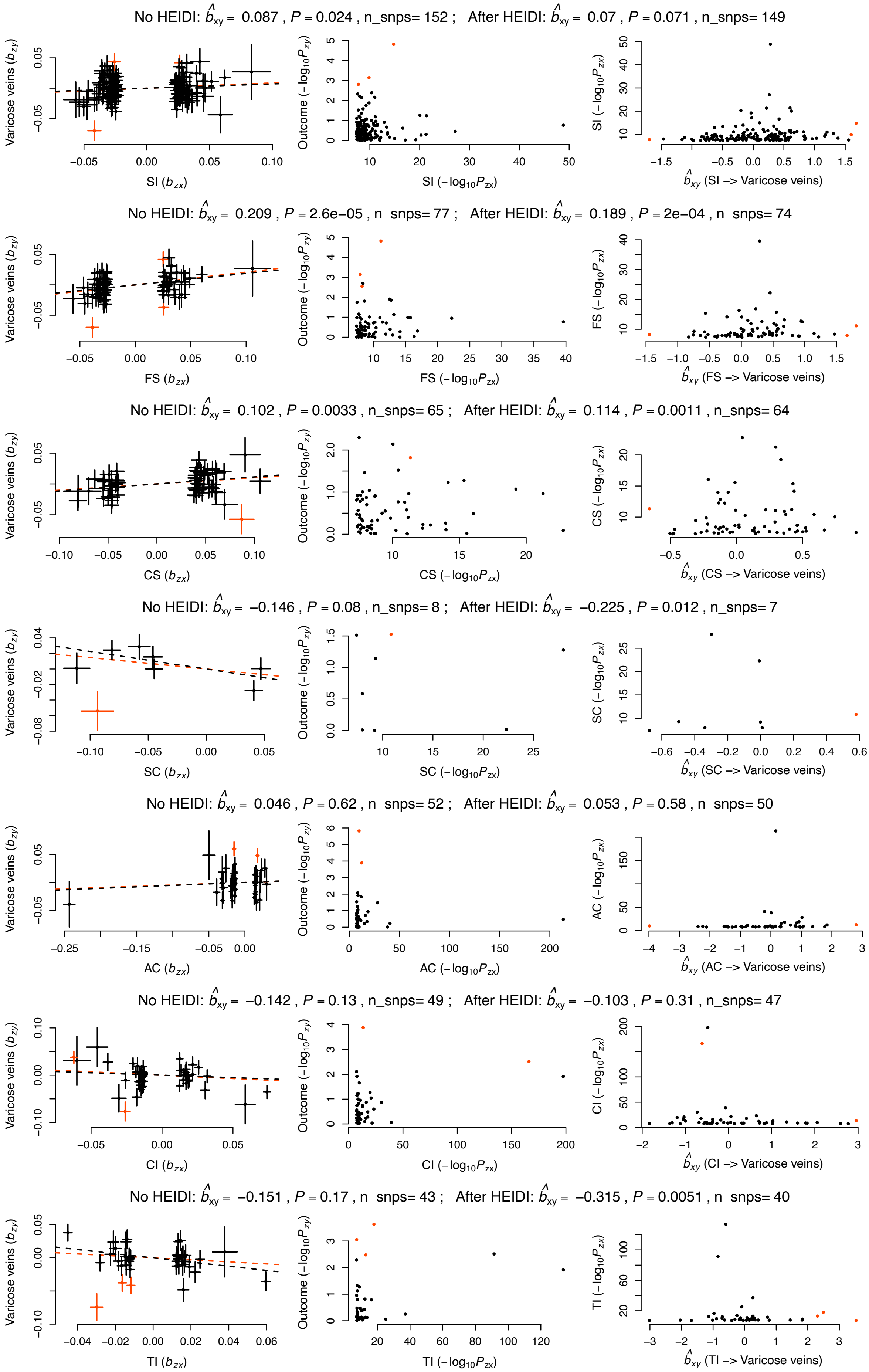

No HEIDI: b̂xy = 0.087 , P = 0.024 , n_snps= 152 ; After HEIDI: b̂xy = 0.07 , P = 0.071 , n_snps= 149
Varicose veins (bzy)
SI (bzx)
Outcome (−log10Pzy)
SI (−log10Pzx)
b̂xy (SI −> Varicose veins)
No HEIDI: b̂xy = 0.209 , P = 2.6e−05 , n_snps= 77 ; After HEIDI: b̂xy = 0.189 , P = 2e−04 , n_snps= 74
Varicose veins (bzy)
FS (bzx)
Outcome (−log10Pzy)
FS (−log10Pzx)
b̂xy (FS −> Varicose veins)
No HEIDI: b̂xy = 0.102 , P = 0.0033 , n_snps= 65 ; After HEIDI: b̂xy = 0.114 , P = 0.0011 , n_snps= 64
Varicose veins (bzy)
CS (bzx)
Outcome (−log10Pzy)
CS (−log10Pzx)
b̂xy (CS −> Varicose veins)
No HEIDI: b̂xy = −0.146 , P = 0.08 , n_snps= 8 ; After HEIDI: b̂xy = −0.225 , P = 0.012 , n_snps= 7
Varicose veins (bzy)
SC (bzx)
Outcome (−log10Pzy)
SC (−log10Pzx)
b̂xy (SC −> Varicose veins)
No HEIDI: b̂xy = 0.046 , P = 0.62 , n_snps= 52 ; After HEIDI: b̂xy = 0.053 , P = 0.58 , n_snps= 50
Varicose veins (bzy)
AC (bzx)
Outcome (−log10Pzy)
AC (−log10Pzx)
b̂xy (AC −> Varicose veins)
No HEIDI: b̂xy = −0.142 , P = 0.13 , n_snps= 49 ; After HEIDI: b̂xy = −0.103 , P = 0.31 , n_snps= 47
Varicose veins (bzy)
CI (bzx)
Outcome (−log10Pzy)
CI (−log10Pzx)
b̂xy (CI −> Varicose veins)
No HEIDI: b̂xy = −0.151 , P = 0.17 , n_snps= 43 ; After HEIDI: b̂xy = −0.315 , P = 0.0051 , n_snps= 40
Varicose veins (bzy)
TI (bzx)
Outcome (−log10Pzy)
TI (−log10Pzx)
b̂xy (TI −> Varicose veins)

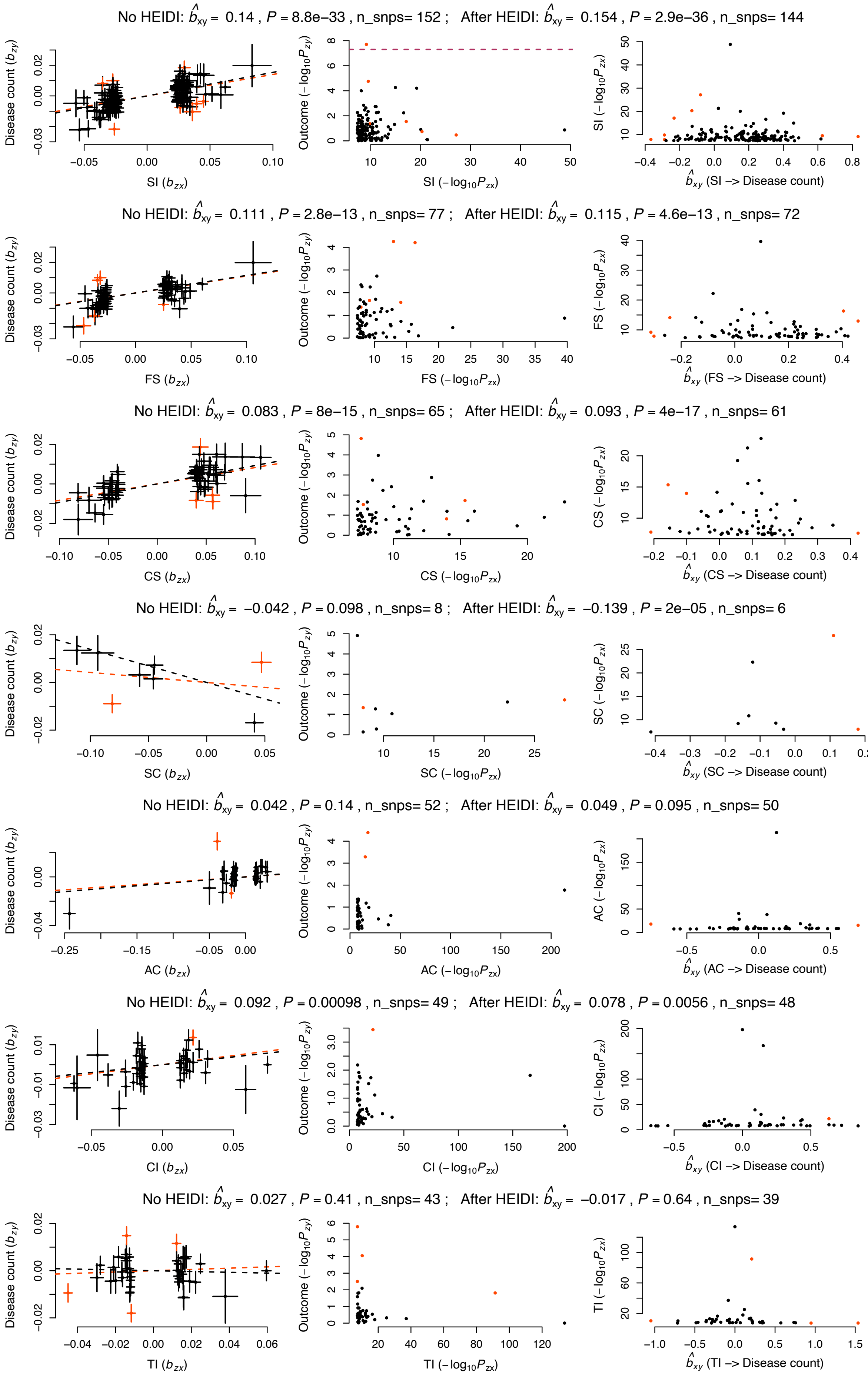
No HEIDI: $\hat{b}_{xy}$ = 0.14 , P = 8.8e−33 , n_snps= 152 ; After HEIDI: $\hat{b}_{xy}$ = 0.154 , P = 2.9e−36 , n_snps= 144
Disease count (b_{zy})
SI (b_{zx})
Outcome ($-\log_{10}P_{zy}$)
SI ($-\log_{10}P_{zx}$)
SI ($-\log_{10}P_{zx}$)
$\hat{b}_{xy}$ (SI -> Disease count)
No HEIDI: $\hat{b}_{xy}$ = 0.111 , P = 2.8e−13 , n_snps= 77 ; After HEIDI: $\hat{b}_{xy}$ = 0.115 , P = 4.6e−13 , n_snps= 72
Disease count (b_{zy})
FS (b_{zx})
Outcome ($-\log_{10}P_{zy}$)
FS ($-\log_{10}P_{zx}$)
FS ($-\log_{10}P_{zx}$)
$\hat{b}_{xy}$ (FS -> Disease count)
No HEIDI: $\hat{b}_{xy}$ = 0.083 , P = 8e−15 , n_snps= 65 ; After HEIDI: $\hat{b}_{xy}$ = 0.093 , P = 4e−17 , n_snps= 61
Disease count (b_{zy})
CS (b_{zx})
Outcome ($-\log_{10}P_{zy}$)
CS ($-\log_{10}P_{zx}$)
CS ($-\log_{10}P_{zx}$)
$\hat{b}_{xy}$ (CS -> Disease count)
No HEIDI: $\hat{b}_{xy}$ = −0.042 , P = 0.098 , n_snps= 8 ; After HEIDI: $\hat{b}_{xy}$ = −0.139 , P = 2e−05 , n_snps= 6
Disease count (b_{zy})
SC (b_{zx})
Outcome ($-\log_{10}P_{zy}$)
SC ($-\log_{10}P_{zx}$)
SC ($-\log_{10}P_{zx}$)
$\hat{b}_{xy}$ (SC -> Disease count)
No HEIDI: $\hat{b}_{xy}$ = 0.042 , P = 0.14 , n_snps= 52 ; After HEIDI: $\hat{b}_{xy}$ = 0.049 , P = 0.095 , n_snps= 50
Disease count (b_{zy})
AC (b_{zx})
Outcome ($-\log_{10}P_{zy}$)
AC ($-\log_{10}P_{zx}$)
AC ($-\log_{10}P_{zx}$)
$\hat{b}_{xy}$ (AC -> Disease count)
No HEIDI: $\hat{b}_{xy}$ = 0.092 , P = 0.00098 , n_snps= 49 ; After HEIDI: $\hat{b}_{xy}$ = 0.078 , P = 0.0056 , n_snps= 48
Disease count (b_{zy})
CI (b_{zx})
Outcome ($-\log_{10}P_{zy}$)
CI ($-\log_{10}P_{zx}$)
CI ($-\log_{10}P_{zx}$)
$\hat{b}_{xy}$ (CI -> Disease count)
No HEIDI: $\hat{b}_{xy}$ = 0.027 , P = 0.41 , n_snps= 43 ; After HEIDI: $\hat{b}_{xy}$ = −0.017 , P = 0.64 , n_snps= 39
Disease count (b_{zy})
TI (b_{zx})
Outcome ($-\log_{10}P_{zy}$)
TI ($-\log_{10}P_{zx}$)
TI ($-\log_{10}P_{zx}$)
$\hat{b}_{xy}$ (TI -> Disease count)

Supplementary Data 1. Causal effect of substance use behaviours on common diseases estimated at individual IVs. Each page indicates one outcome, respectively. The subtitle on the top of each layer shows the causal estimate before and after the HEIDI-outlier filtering. The pleiotropic IVs detected by the HEIDI-outlier test are highlighted in red. In each layer within in one page, the left column shows the SNP effect estimates for the exposure (x-axis) plotted against those for the outcome (y-axis). Each error bar indicates the *s.e.* of the SNP effect estimate. The slope of the red and black dashed line indicates $\hat{b}_{xy}$ before and after HEIDI-outlier filtering, respectively. The middle column shows the -$\log_{10}(P)$ of each IV for exposure (x-axis) against that for outcome (y-axis). The right column shows $\hat{b}_{xy}$ for each IV (x-axis) against the corresponding GWAS -$\log_{10}(P)$ for the exposure (y-axis). The error bar indicates the standard error of the $\hat{b}_{xy}$.
