# Supplementary material for: Unravelling the complex causal effects of substance use behaviours on common diseases
Source: Commun Med (Lond). 2024 Mar 12;4:43. doi: 10.1038/s43856-024-00473-3 (PMC10933313; doi:10.1038/s43856-024-00473-3)
Supplement: Supplementary file 17 — Reporting Summary [file 43856_2024_473_MOESM17_ESM.pdf]

nature portfolio

Corresponding author(s): Jian Yang

Last updated by author(s): Dec 26, 2023

Reporting Summary

Nature Portfolio wishes to improve the reproducibility of the work that we publish. This form provides structure for consistency and transparency in reporting. For further information on Nature Portfolio policies, see our Editorial Policies and the Editorial Policy Checklist.

Statistics

For all statistical analyses, confirm that the following items are present in the figure legend, table legend, main text, or Methods section.

n/a	Confirmed	
☐	☒	The exact sample size (*n*) for each experimental group/condition, given as a discrete number and unit of measurement
☐	☒	A statement on whether measurements were taken from distinct samples or whether the same sample was measured repeatedly
☐	☒	The statistical test(s) used AND whether they are one- or two-sided<br>*Only common tests should be described solely by name; describe more complex techniques in the Methods section.*
☐	☒	A description of all covariates tested
☐	☒	A description of any assumptions or corrections, such as tests of normality and adjustment for multiple comparisons
☐	☒	A full description of the statistical parameters including central tendency (e.g. means) or other basic estimates (e.g. regression coefficient) AND variation (e.g. standard deviation) or associated estimates of uncertainty (e.g. confidence intervals)
☐	☒	For null hypothesis testing, the test statistic (e.g. *F*, *t*, *r*) with confidence intervals, effect sizes, degrees of freedom and *P* value noted<br>*Give P values as exact values whenever suitable.*
☒	☐	For Bayesian analysis, information on the choice of priors and Markov chain Monte Carlo settings
☒	☐	For hierarchical and complex designs, identification of the appropriate level for tests and full reporting of outcomes
☐	☒	Estimates of effect sizes (e.g. Cohen's *d*, Pearson's *r*), indicating how they were calculated

Our web collection on statistics for biologists contains articles on many of the points above.

Software and code

Policy information about availability of computer code

Data collection	No data-collection software was used.
Data analysis	We used PLINK v1.90beta for quality control of the genotyped or imputed SNP data, BOLT-LMM v2.2 for GWAS analyses, and LDSC v1.0.0 for the estimation of SNP-based heritability and genetic correlation. We employed eleven MR methods to estimate the causal relationship between traits. These methods include GSMR2 (implemented in GCTA v1.93.0b), IVW, Robust, MR-Egger, weighted median, mode, and Con-Mix, implemented in the R package "MendelianRandomization" (v0.4.2), and MR-Lasso (bioRxiv version), MR-PRESSO (v1.0), MRMix (v0.1.0), and RAPS (v0.2), implemented in R (v4.0.5). Data visualization was conducted using RStudio v1.2.1335 with R version 4.0.2.

For manuscripts utilizing custom algorithms or software that are central to the research but not yet described in published literature, software must be made available to editors and reviewers. We strongly encourage code deposition in a community repository (e.g. GitHub). See the Nature Portfolio guidelines for submitting code & software for further information.

Data

Policy information about availability of data

All manuscripts must include a data availability statement. This statement should provide the following information, where applicable:

- Accession codes, unique identifiers, or web links for publicly available datasets
- A description of any restrictions on data availability
- For clinical datasets or third party data, please ensure that the statement adheres to our policy

This study was conducted using data from the UK Biobank (UKB) under Application Number 12505, which is available through the UK Biobank Access Management System (https://www.ukbiobank.ac.uk/). The GWAS summary statistics from the GSCAN consortium can be downloaded online (https://genome.psych.umn.edu/index.php/GSCAN). The twelve genome-wide association study (GWAS) summary datasets for common diseases from published studies are described in Table S12. GWAS summary statistics for the seven SUB traits are available at https://yanglab.westlake.edu.cn/pub_data.html.

Human research participants

Policy information about studies involving human research participants and Sex and Gender in Research.

Reporting on sex and gender	In this study, only self-reported sex was used in the GWAS analysis, and individuals with inconsistent self-reported sex and biological sex based on genotype were pre-excluded.
Population characteristics	The human research participants from the UK Biobank ranged in age from 37 to 73, residing across the United Kingdom (UK), with approximately 54% of participants being women. The genotype data were collected using a customized Affymetrix Axiom array. The raw genotype data were imputed into the Human Reference Consortium (HRC) panel by the UK Biobank team. We included only a subset of individuals of European ancestry in the analysis (n = 456,426). The phenotypic records of common diseases in the UK Biobank were obtained from the ICD10 (International Classification of Diseases, 10th Revision) primary diagnoses, ICD10 secondary diagnoses, and self-report records.
Recruitment	This study utilizes data from the UK Biobank (project ID: 12505). The UK Biobank is a national and international health resource, with approximately 500,000 volunteer participants who have provided genotypic and phenotypic information. Participants were recruited between 2006 and 2010 and attended one of the 22 assessment centers in the UK.
Ethics oversight	The UK Biobank has ethical approval from the North West Multi-centre Research Ethics Committee (MREC), which covers the UK. Additionally, it has approval in England and Wales from the Patient Information Advisory Group (PIAG) for gaining access to information that allows for participant invitation. PIAG has since been replaced by the National Information Governance Board for Health & Social Care (NIGB). In Scotland, the UK Biobank has approval from the Community Health Index Advisory Group (CHIAG). This study has been approved by both the University of Queensland Human Research Ethics Committee (approval number: 2011001173) and the Westlake University Ethics Committee (approval number: 20200722YJ001).

Note that full information on the approval of the study protocol must also be provided in the manuscript.

Field-specific reporting

Please select the one below that is the best fit for your research. If you are not sure, read the appropriate sections before making your selection.

☒ Life sciences ☐ Behavioural & social sciences ☐ Ecological, evolutionary & environmental sciences

For a reference copy of the document with all sections, see nature.com/documents/nr-reporting-summary-flat.pdf

Life sciences study design

All studies must disclose on these points even when the disclosure is negative.

Sample size	The sample sizes were determined based on the availability of GWAS datasets. For published GWAS, the sample sizes ranged from 16,731 to 547,261. For in-house GWAS using the UK Biobank data, the sample sizes ranged from 208,988 to 454,648 for substance use behaviors and 454,108 to 455,607 for the 18 common diseases.
Data exclusions	We excluded individuals not of European ancestry from the UK Biobank. We removed SNPs with a minor allele count of less than 5, a Hardy-Weinberg equilibrium test P-value of less than 1E-6, a missing genotype rate of more than 5%, or an imputation info score of less than 0.3.
Replication	The main MR analysis was replicated using data from published studies for both substance use behaviors and health outcomes. The causal effects estimated using the published disease GWAS data were highly correlated with those estimated using the UK Biobank disease data, despite slight differences in the phenotypic definitions of the diseases between studies.
Randomization	NA. We did not use any study design that required randonmization.
Blinding	NA. We did not use any study design that required blinding.

Reporting for specific materials, systems and methods

We require information from authors about some types of materials, experimental systems and methods used in many studies. Here, indicate whether each material, system or method listed is relevant to your study. If you are not sure if a list item applies to your research, read the appropriate section before selecting a response.

Materials & experimental systems

n/a	Involved in the study
☒	☐ Antibodies
☒	☐ Eukaryotic cell lines
☒	☐ Palaeontology and archaeology
☒	☐ Animals and other organisms
☒	☐ Clinical data
☒	☐ Dual use research of concern

Methods

n/a	Involved in the study
☒	☐ ChIP-seq
☒	☐ Flow cytometry
☒	☐ MRI-based neuroimaging
